# Supplementary material for: Germline Polymorphisms Associated with Overall Survival in Lung Adenocarcinoma: Genome-Wide Analysis
Source: Cancers (Basel). 2024 Sep 25;16(19):3264. doi: 10.3390/cancers16193264 (PMC11475969; doi:10.3390/cancers16193264)
Supplement: Supplementary file 1 [file cancers-16-03264-s001.zip › Supplementary Figure S1.pdf]

Supplementary Figure S1:

Per-sample and per-marker quality control (QC) of genotyping data. HWE, Harvey-Weinberg equilibrium; MAF, minor allele frequency

(**A**)

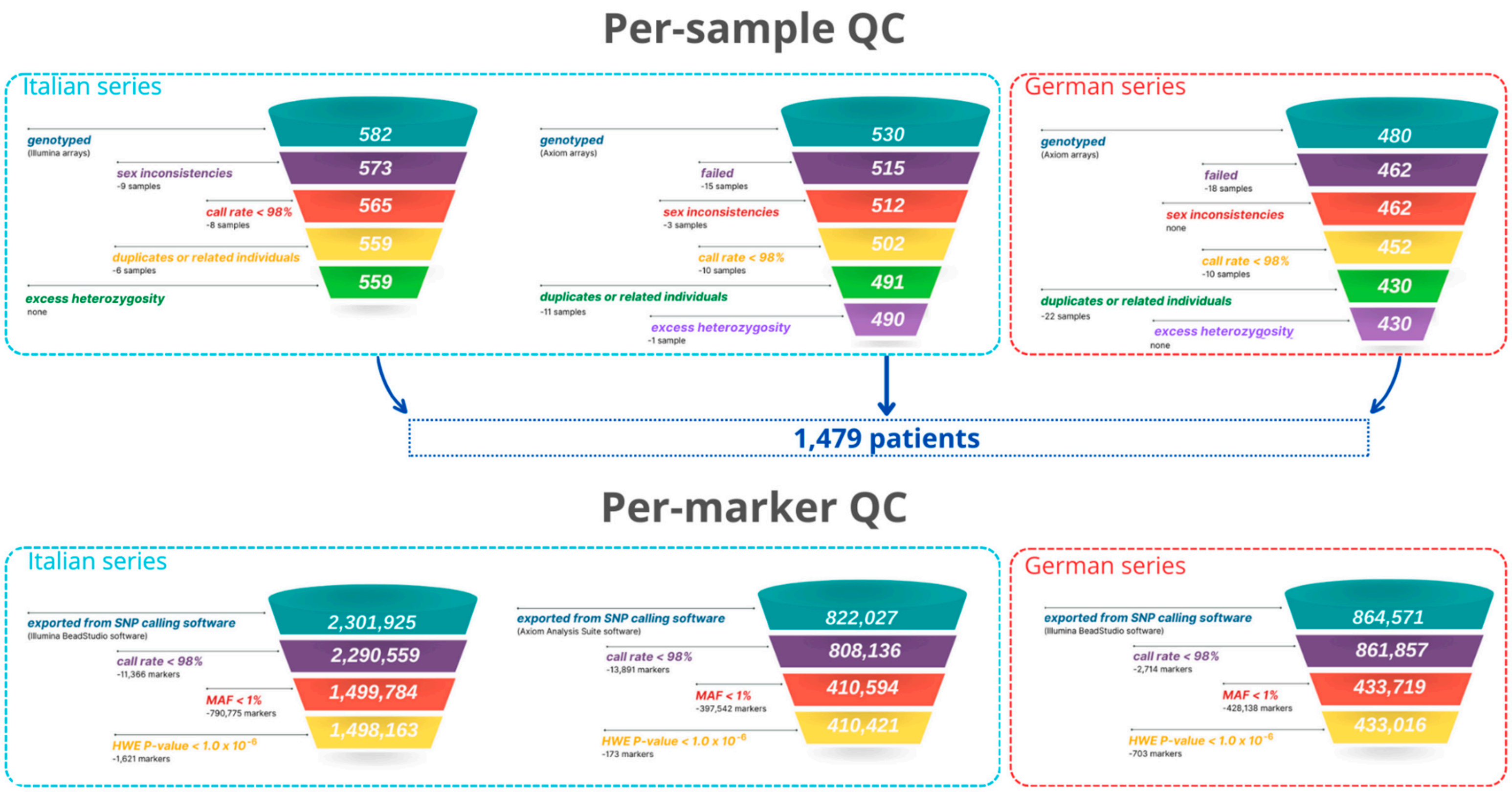

(**B**)
