# Supplementary material for: Germline Polymorphisms Associated with Overall Survival in Lung Adenocarcinoma: Genome-Wide Analysis
Source: Cancers (Basel). 2024 Sep 25;16(19):3264. doi: 10.3390/cancers16193264 (PMC11475969; doi:10.3390/cancers16193264)

Supplementary Figure S2:

Plots of the first four principal components. PC1 and PC2 (A) and PC3 and PC4 (B) of our dataset (pink dots, n=1,479) are plotted along with the same PCs of 2,504 individuals from five populations from the 1000 Genomes Project (AFR, Africans; AMR, Americans; EAS, East Asians; EUR, Europeans; SAS, South Asians).

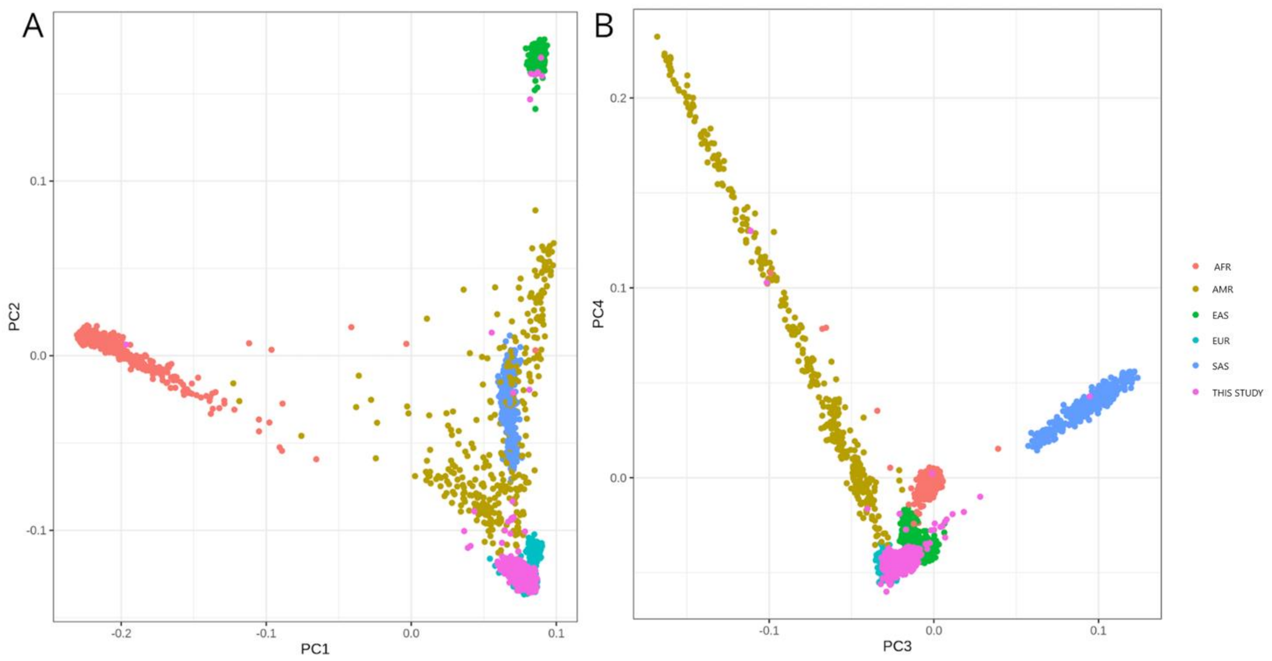

Supplement: Supplementary file 1 [file cancers-16-03264-s001.zip › Supplementary Figure S2.pdf]
