# Supplementary material for: Germline Polymorphisms Associated with Overall Survival in Lung Adenocarcinoma: Genome-Wide Analysis
Source: Cancers (Basel). 2024 Sep 25;16(19):3264. doi: 10.3390/cancers16193264 (PMC11475969; doi:10.3390/cancers16193264)
Supplement: Supplementary file 1 [file cancers-16-03264-s001.zip › Supplementary Figure S3.pdf]

Supplementary Figure S3:

Kaplan–Meier survival curves (truncated at 60 months) for lung adenocarcinoma patients according to tumor expression levels of *NT5DC2*, *TKT*, *UQCC5*, and *NAGK* genes.

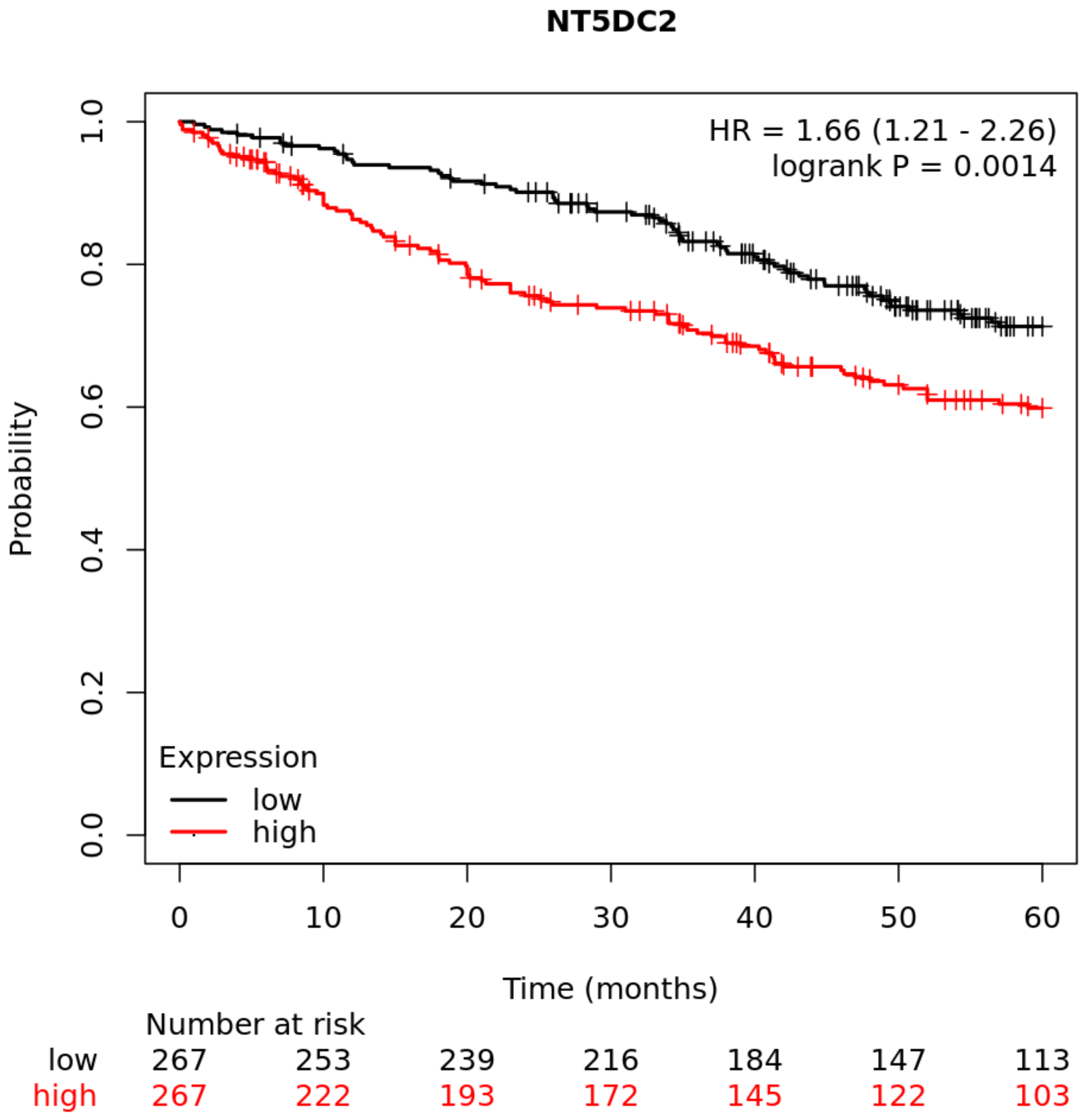

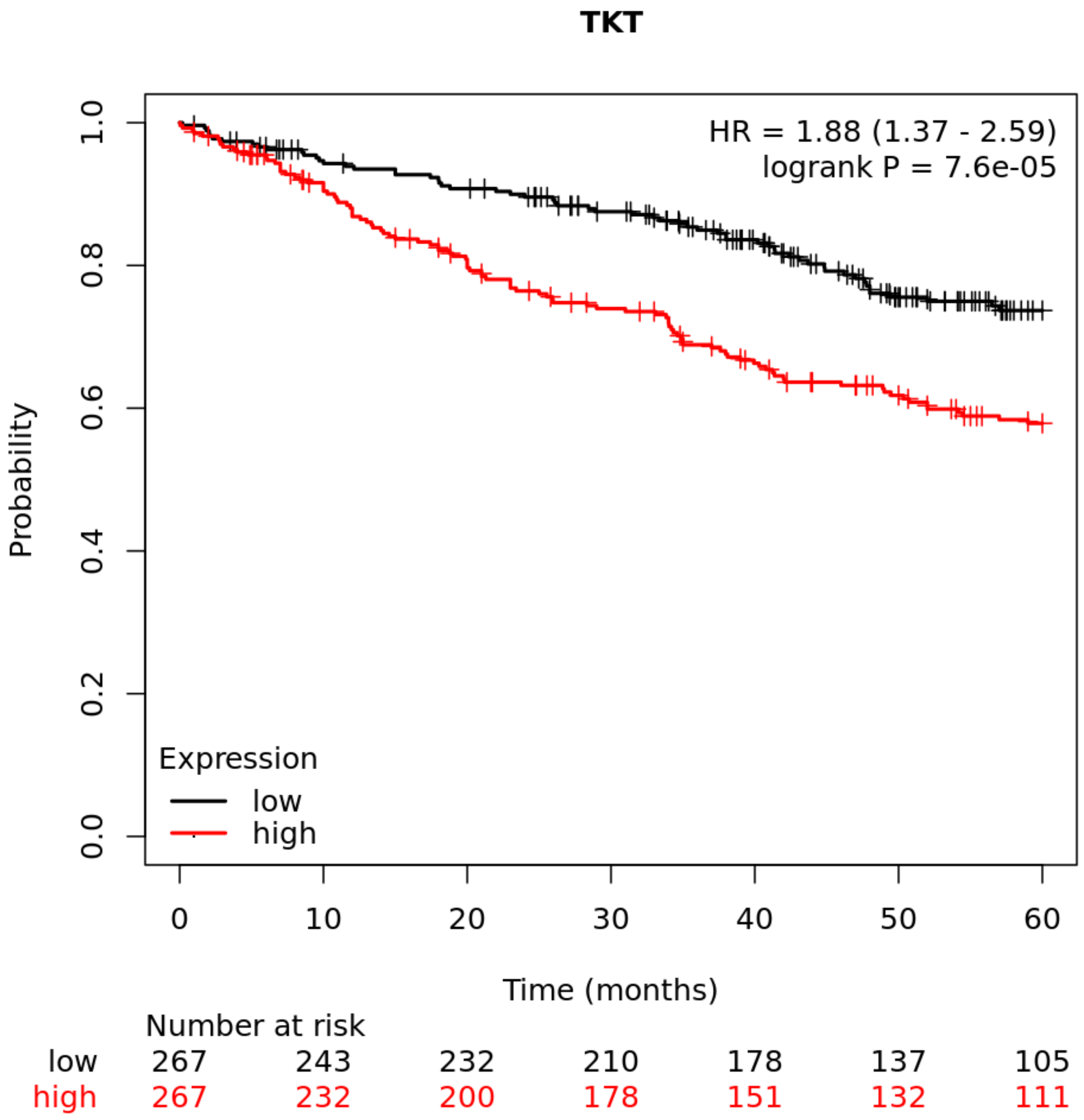

TKT
HR = 1.88 (1.37 - 2.59)
logrank P = 7.6e-05
Probability
0.0
0.2
0.4
0.6
0.8
1.0
Expression
low
high
0
10
20
30
40
50
60
Time (months)
Number at risk
low 267 243 232 210 178 137 105
high 267 232 200 178 151 132 111

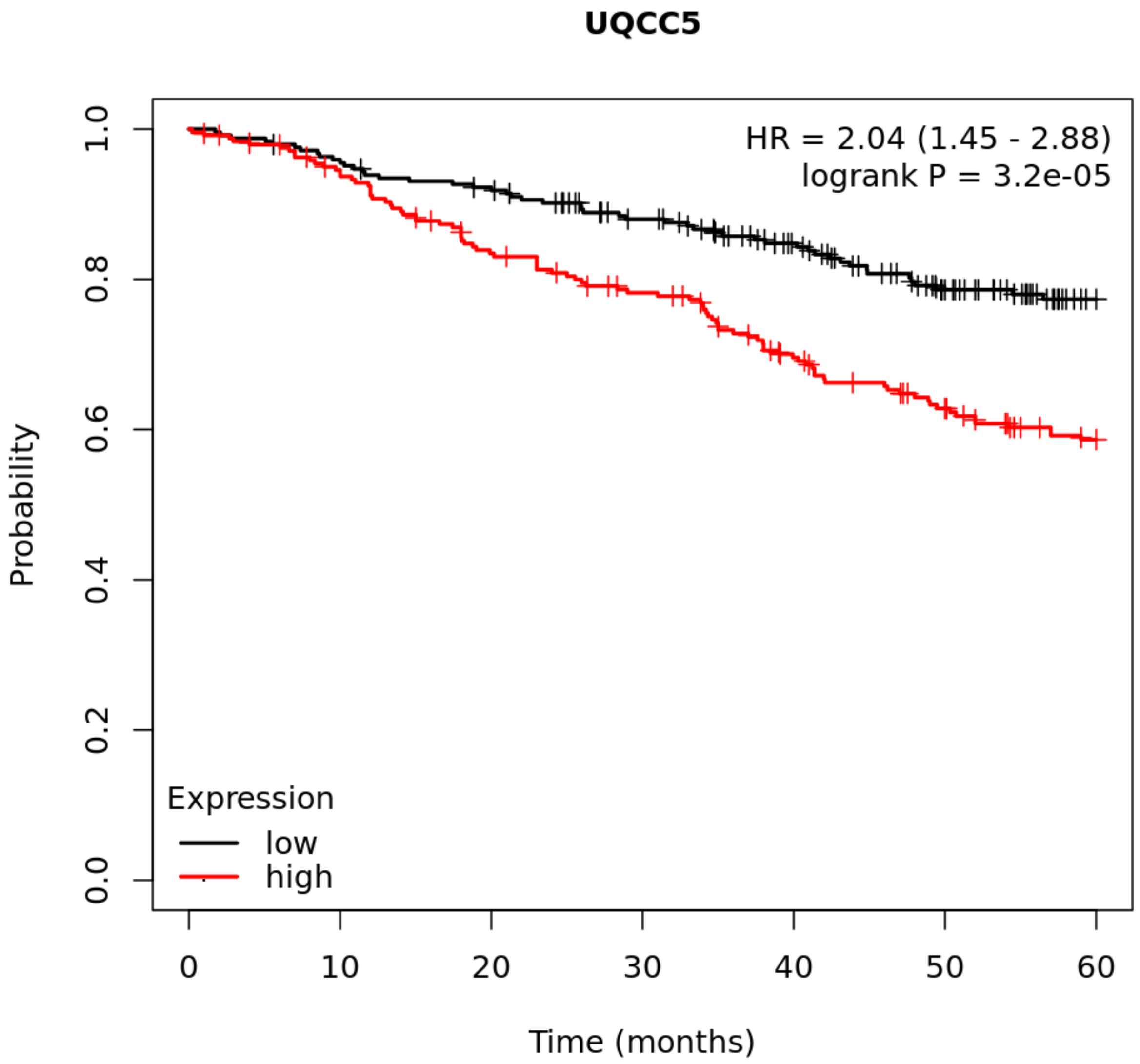

Number at risk

low	246	235	224	200	173	140	109
high	243	223	193	176	148	127	106

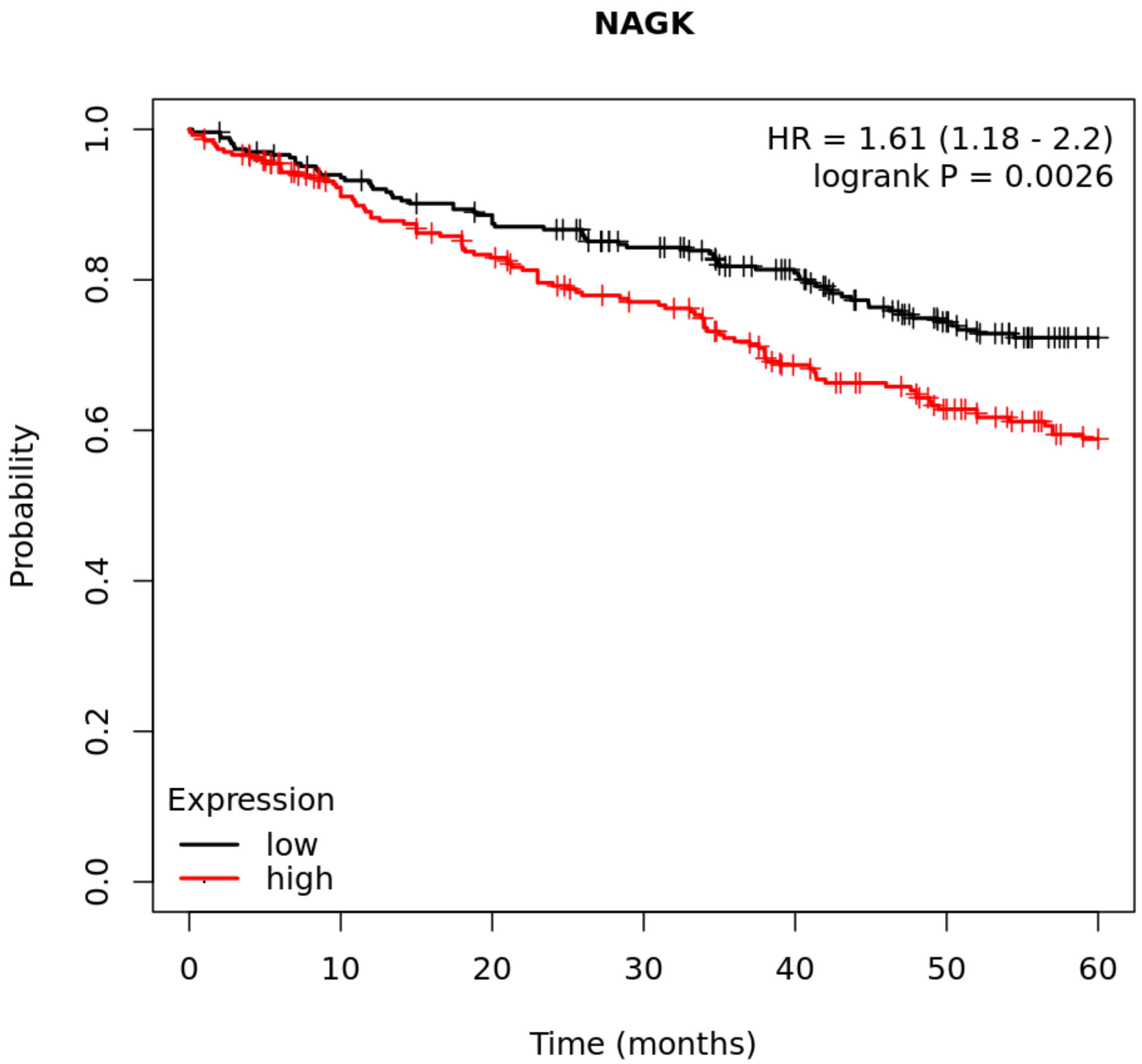

Number at risk

low	267	247	230	209	183	147	120
high	267	228	202	179	146	122	96
