# Supplementary material for: Germline Polymorphisms Associated with Overall Survival in Lung Adenocarcinoma: Genome-Wide Analysis
Source: Cancers (Basel). 2024 Sep 25;16(19):3264. doi: 10.3390/cancers16193264 (PMC11475969; doi:10.3390/cancers16193264)
Supplement: Supplementary file 1 [file cancers-16-03264-s001.zip › SupplementaryTable S1.pdf]

Supplementary Table S1. Genetic variants associated with lung adenocarcinoma survival (60 months after surgery) at $P < 1.0 \times 10^{-5}$, sorted by *P*-value. Genomic positions refer to GRCh38 realease.

SNP ID	Chromosome	Genomic position (bp)	Reference allele	Alternative allele	MAF	HR	*P*-value	FDR	Gene	Gene start	Gene end	SNP location
rs74464684	3	53,081,611	C	T	0.017	2.77	4.96E-09	0.03	ENSG00000272305	52,969,119	53,099,453	intronic
rs13000315	2	70,945,905	T	A	0.023	2.45	1.39E-08	0.03	VAX2	70,900,576	70,965,373	intronic
rs71414848	2	70,946,048	T	C	0.023	2.45	1.39E-08	0.03	ATP6V1B1	70,935,900	70,965,431	intronic
rs76553845	3	53,024,096	A	G	0.016	2.73	2.02E-08	0.04	SFMBT1	52,903,572	53,046,750	intronic
rs151212827	3	52,957,703	G	A	0.017	2.62	2.38E-08	0.04	SFMBT1	52,903,572	53,046,750	intronic
rs190923216	5	81,295,429	T	A	0.016	2.86	4.08E-08	0.05	CKMT2	233,320	81,266,398	intronic
rs77782057	3	52,853,366	G	C	0.017	2.59	5.25E-08	0.06	STIMATE-MUSTN1	52,833,121	52,897,562	intronic
rs529405630	2	70,943,399	G	A	0.022	2.37	9.79E-08	0.08	VAX2	70,900,576	70,965,373	non-coding transcript exon
rs562153838	2	70,943,398	G	C	0.022	2.37	9.79E-08	0.08	ATP6V1B1	70,935,900	70,965,431	non-coding transcript exon
rs74870948	4	24,970,377	G	A	0.027	2.25	2.03E-07	0.13	CCDC149	24,807,996	24,980,148	intronic
rs34456496	2	70,921,463	C	T	0.022	2.44	2.31E-07	0.13	VAX2	70,900,576	70,965,373	intronic
rs34566971	2	70,918,144	T	C	0.022	2.44	2.31E-07	0.13	VAX2	70,900,576	70,965,373	intronic
rs117717456	13	84,998,984	T	C	0.023	2.38	3.32E-07	0.13	-	-	-	intergenic
rs34888497	2	70,940,022	G	A	0.022	2.31	3.36E-07	0.13	VAX2	70,900,576	70,965,373	3'-UTR region
rs13022751	2	70,938,233	C	T	0.024	2.23	4.08E-07	0.13	VAX2	70,900,576	70,965,373	intronic
rs186956810	3	53,033,317	C	T	0.015	2.59	4.32E-07	0.13	SFMBT1	52,903,572	53,046,750	intronic
rs72960281	3	52,768,855	C	T	0.020	2.35	4.48E-07	0.13	NEK4	52,708,444	52,770,940	intronic
rs113071416	3	52,764,132	A	G	0.020	2.35	4.48E-07	0.13	NEK4	52,708,444	52,770,940	intronic
rs7619643	3	52,754,103	G	A	0.020	2.35	4.48E-07	0.13	NEK4	52,708,444	52,770,940	intronic
rs6445536	3	52,751,167	G	A	0.020	2.35	4.48E-07	0.13	NEK4	52,708,444	52,770,940	intronic
rs151032151	3	52,741,024	G	A	0.020	2.35	4.48E-07	0.13	NEK4	52,708,444	52,770,940	intronic
rs74429279	3	52,732,241	C	G	0.020	2.35	4.48E-07	0.13	NEK4	52,708,444	52,770,940	intronic
rs6769720	3	52,730,334	T	G	0.020	2.35	4.48E-07	0.13	NEK4	52,708,444	52,770,940	intronic
rs72960240	3	52,725,117	A	C	0.020	2.35	4.48E-07	0.13	NEK4	52,708,444	52,770,940	intronic
rs56837675	3	52,716,277	C	T	0.020	2.35	4.48E-07	0.13	NEK4	52,708,444	52,770,940	intronic
rs139910077	3	2,164,331	C	T	0.018	2.65	5.09E-07	0.13	CNTN4	2,098,813	2,745,687	intronic
rs74033708	16	86,497,152	C	A	0.072	1.72	6.27E-07	0.13	FENDRR	86,474,529	86,508,860	intronic
rs2193160	12	5,531,947	T	C	0.372	0.72	6.86E-07	0.13	ANO2	5,531,869	5,563,337	non-coding transcript exon
rs34529435	8	125,504,002	G	A	0.039	1.96	7.03E-07	0.13	TRIB1AL	125,466,939	125,541,373	intronic
rs10164686	2	70,940,001	G	A	0.022	2.23	7.14E-07	0.13	ATP6V1B1	70,935,900	70,965,431	intronic
rs17322079	8	125,507,429	C	T	0.024	2.21	7.34E-07	0.13	TRIB1AL	125,466,939	125,541,373	intronic
rs117400654	8	125,504,966	A	G	0.024	2.21	7.34E-07	0.13	TRIB1AL	125,466,939	125,541,373	intronic
rs117421262	8	125,504,391	A	G	0.024	2.21	7.34E-07	0.13	TRIB1AL	125,466,939	125,541,373	intronic
rs76252265	3	52,846,426	G	A	0.015	2.51	7.37E-07	0.13	STIMATE	52,836,733	52,897,548	intronic
rs7314761	12	5,535,399	C	T	0.370	0.72	7.39E-07	0.13	ANO2	5,531,869	5,563,337	intronic
rs143897445	12	125,990,705	G	C	0.010	2.82	7.41E-07	0.13	LINC02826	125,983,708	126,043,480	intronic
rs116578433	6	13,566,151	A	G	0.021	2.43	7.50E-07	0.13	-	-	-	intergenic
rs9547043	13	85,019,845	C	A	0.023	2.34	7.82E-07	0.13	-	-	-	intergenic
rs545672486	9	97,448,699	G	A	0.018	2.36	8.55E-07	0.13	TDRD7	97,412,096	97,496,125	intronic

rs575271524	9	97,448,698	T	A	0.018	2.36	8.55E-07	0.13	TDRD7	97,412,096	97,496,125	intronic
rs6489632	12	5,530,688	G	A	0.370	0.72	9.13E-07	0.13	-	-	-	intergenic
rs34195894	2	70,936,224	G	C	0.021	2.38	9.67E-07	0.13	VAX2	70,900,576	70,965,373	intronic
rs34965400	2	70,936,147	G	A	0.021	2.38	9.67E-07	0.13	VAX2	70,900,576	70,965,373	intronic
rs35768563	2	70,936,090	C	T	0.021	2.38	9.67E-07	0.13	VAX2	70,900,576	70,965,373	intronic
rs71414846	2	70,935,632	C	T	0.021	2.38	9.67E-07	0.13	VAX2	70,900,576	70,965,373	intronic
rs35096861	2	70,935,278	C	T	0.021	2.38	9.67E-07	0.13	VAX2	70,900,576	70,965,373	intronic
rs138689441	2	70,932,225	G	A	0.021	2.38	9.67E-07	0.13	VAX2	70,900,576	70,965,373	intronic
rs35791459	2	70,930,960	T	C	0.021	2.38	9.67E-07	0.13	VAX2	70,900,576	70,965,373	intronic
rs35921308	2	70,930,710	A	T	0.021	2.38	9.67E-07	0.13	VAX2	70,900,576	70,965,373	intronic
rs34315744	2	70,930,495	C	A	0.021	2.38	9.67E-07	0.13	VAX2	70,900,576	70,965,373	intronic
rs76599771	8	125,501,476	G	A	0.024	2.21	9.91E-07	0.13	TRIB1AL	125,466,939	125,541,373	intronic
rs74582920	3	105,935,398	C	T	0.013	2.76	1.07E-06	0.13	-	-	-	intergenic
rs140892559	3	105,896,971	A	T	0.013	2.75	1.10E-06	0.13	-	-	-	intergenic
rs7314197	12	5,538,528	C	T	0.369	0.72	1.13E-06	0.13	ANO2	5,531,869	5,563,337	intronic
rs12315349	12	5,536,179	T	C	0.369	0.72	1.13E-06	0.13	ANO2	5,531,869	5,563,337	intronic
rs72960285	3	52,773,800	T	G	0.019	2.30	1.21E-06	0.13	-	-	-	intergenic
rs185761183	3	52,773,429	T	C	0.019	2.30	1.21E-06	0.13	-	-	-	intergenic
rs191101411	3	52,768,817	C	G	0.019	2.30	1.21E-06	0.13	NEK4	52,708,444	52,770,940	intronic
rs115801090	3	52,762,646	G	A	0.019	2.30	1.21E-06	0.13	NEK4	52,708,444	52,770,940	intronic
rs114707129	3	52,750,191	A	G	0.019	2.30	1.21E-06	0.13	NEK4	52,708,444	52,770,940	intronic
rs190297652	3	52,749,370	G	A	0.019	2.30	1.21E-06	0.13	NEK4	52,708,444	52,770,940	intronic
rs181612199	3	52,745,959	T	C	0.019	2.30	1.21E-06	0.13	NEK4	52,708,444	52,770,940	intronic
rs75321479	3	52,744,194	G	A	0.019	2.30	1.21E-06	0.13	NEK4	52,708,444	52,770,940	intronic
rs78014137	3	52,742,762	T	C	0.019	2.30	1.21E-06	0.13	NEK4	52,708,444	52,770,940	intronic
rs114687381	3	52,731,933	G	A	0.019	2.30	1.21E-06	0.13	NEK4	52,708,444	52,770,940	intronic
rs116493126	3	52,716,309	T	C	0.019	2.30	1.21E-06	0.13	NEK4	52,708,444	52,770,940	intronic
rs11063739	12	5,533,389	A	G	0.371	0.72	1.23E-06	0.13	ANO2	5,531,869	5,563,337	intronic
rs11063738	12	5,533,221	A	G	0.371	0.72	1.23E-06	0.13	ANO2	5,531,869	5,563,337	intronic
rs879105	12	5,532,191	A	C	0.371	0.72	1.23E-06	0.13	ANO2	5,531,869	5,563,337	intronic
rs10495213	1	223,683,355	G	A	0.099	1.57	1.24E-06	0.13	-	-	-	intergenic
rs62576163	9	81,623,046	T	A	0.014	2.70	1.24E-06	0.13	TLE1	81,583,683	81,689,547	intronic
rs72657503	8	125,509,948	A	C	0.025	2.16	1.48E-06	0.15	TRIB1AL	125,466,939	125,541,373	intronic
rs56354394	2	70,933,937	G	A	0.103	1.55	1.51E-06	0.15	VAX2	70,900,576	70,965,373	intronic
rs7398813	12	5,544,700	T	A	0.370	0.73	1.57E-06	0.16	ANO2	5,531,869	5,563,337	intronic
rs7398812	12	5,544,484	T	A	0.370	0.73	1.69E-06	0.16	ANO2	5,531,869	5,563,337	intronic
rs72667827	8	91,415,287	T	A	0.013	2.66	2.15E-06	0.19	-	-	-	intronic
rs530692238	15	91,470,825	C	G	0.120	1.48	2.27E-06	0.19	CRAT37	91,408,708	91,605,873	intronic
rs72657504	8	125,509,988	G	A	0.025	2.13	2.39E-06	0.19	TRIB1AL	125,466,939	125,541,373	intronic
rs56361051	1	223,658,737	C	T	0.095	1.56	2.42E-06	0.19	CAPN8	223,541,616	223,665,701	intronic
rs72820909	10	93,492,768	T	G	0.029	2.13	2.43E-06	0.19	-	-	-	intergenic
rs6467310	7	130,628,875	C	T	0.047	1.81	2.44E-06	0.19	COPG2	130,506,238	130,668,748	intronic
rs113095049	4	10,722,798	C	T	0.013	2.72	2.59E-06	0.19	-	-	-	regulatory region

rs10002387	4	10,720,648	T	C	0.013	2.72	2.59E-06	0.19	-	-	-	non-coding transcript exon
rs10001979	4	10,720,244	T	C	0.013	2.72	2.59E-06	0.19	-	-	-	non-coding transcript exon
rs76769559	4	10,719,546	A	G	0.013	2.72	2.59E-06	0.19	-	-	-	non-coding transcript exon
rs75211389	15	66,164,538	A	G	0.075	1.64	2.68E-06	0.19	MEGF11	65,895,079	66,253,747	intronic
rs35944830	5	163,525,554	T	G	0.017	2.42	2.68E-06	0.19	-	-	-	intronic
-	16	86,471,937	C	T	0.030	2.01	2.73E-06	0.19	indel			-
rs71597999	5	163,462,384	C	A	0.017	2.41	3.15E-06	0.19	HMMR	163,460,203	163,471,463	intronic
rs76759198	3	52,563,010	C	T	0.018	2.24	3.29E-06	0.19	UQCC5 - PBMR1			intronic
rs80332599	3	52,556,552	T	C	0.018	2.24	3.29E-06	0.19	UQCC5 - PBMR1			intronic
rs73837804	3	52,539,996	G	C	0.018	2.24	3.29E-06	0.19	UQCC5			intronic
rs7899499	10	89,828,244	C	T	0.132	1.47	3.30E-06	0.19	-	-	-	intronic
rs77698041	12	125,992,539	C	T	0.012	2.60	3.33E-06	0.19	LINC02826			intronic
rs140728965	3	52,709,131	C	T	0.019	2.24	3.34E-06	0.19	SPCS1	52,706,106	52,711,148	intronic
rs79705974	3	52,702,426	G	A	0.019	2.24	3.34E-06	0.19	GLT8D1	52,694,486	52,705,791	intronic
rs188074306	3	52,677,576	C	T	0.019	2.24	3.34E-06	0.19	PBMR1	52,545,352	52,679,714	intronic
rs73839126	3	52,672,109	C	T	0.019	2.24	3.34E-06	0.19	PBMR1	52,545,352	52,679,714	intronic
rs80214269	3	52,654,730	C	T	0.019	2.24	3.34E-06	0.19	PBMR1	52,545,352	52,679,714	intronic
rs60040519	3	52,641,565	T	C	0.019	2.24	3.34E-06	0.19	PBMR1	52,545,352	52,679,714	intronic
rs60830326	3	52,639,775	C	T	0.019	2.24	3.34E-06	0.19	PBMR1	52,545,352	52,679,714	intronic
rs79957220	3	52,637,839	C	T	0.019	2.24	3.34E-06	0.19	PBMR1	52,545,352	52,679,714	intronic
rs78735727	3	52,606,100	G	A	0.019	2.24	3.34E-06	0.19	PBMR1	52,545,352	52,679,714	intronic
rs77711437	3	52,590,493	T	G	0.019	2.24	3.34E-06	0.19	PBMR1	52,545,352	52,679,714	intronic
rs114577020	3	52,572,154	T	C	0.019	2.24	3.34E-06	0.19	PBMR1	52,545,352	52,679,714	intronic
rs186146073	3	52,569,345	G	C	0.019	2.24	3.34E-06	0.19	PBMR1	52,545,352	52,679,714	intronic
rs74686130	3	52,566,122	C	T	0.019	2.24	3.34E-06	0.19	PBMR1	52,545,352	52,679,714	intronic
rs12265484	10	89,824,818	G	A	0.134	1.47	3.46E-06	0.19	-	-	-	intergenic
rs4933514	10	89,824,476	T	C	0.134	1.47	3.46E-06	0.19	-	-	-	intergenic
rs4933161	10	89,824,422	T	G	0.134	1.47	3.46E-06	0.19	-	-	-	intergenic
rs4933160	10	89,824,354	T	C	0.134	1.47	3.46E-06	0.19	-	-	-	intergenic
rs35494197	10	89,823,973	T	C	0.134	1.47	3.46E-06	0.19	-	-	-	intergenic
rs17127746	10	89,823,936	C	T	0.134	1.47	3.46E-06	0.19	-	-	-	intergenic
rs6467311	7	130,655,148	A	C	0.047	1.79	3.52E-06	0.19	COPG2	130,506,238	130,668,748	intronic
rs13237881	7	130,649,969	G	T	0.047	1.79	3.52E-06	0.19	COPG2	130,506,238	130,668,748	intronic
rs10954274	7	130,649,535	C	A	0.047	1.79	3.52E-06	0.19	COPG2	130,506,238	130,668,748	intronic
rs10762653	10	75,078,296	A	G	0.136	1.48	3.58E-06	0.19	COPG2	130,506,238	130,668,748	intronic
rs1987234	3	52,564,343	T	C	0.020	2.19	3.71E-06	0.19	PBMR1	52,545,352	52,679,714	intronic
rs58725214	3	52,559,198	T	C	0.020	2.19	3.71E-06	0.19	PBMR1	52,545,352	52,679,714	intronic
rs58100002	3	52,555,824	T	C	0.020	2.19	3.71E-06	0.19	PBMR1	52,545,352	52,679,714	intronic
rs57560655	3	52,543,318	G	A	0.020	2.19	3.71E-06	0.19	PBMR1	52,545,352	52,679,714	intronic
rs57319306	3	52,541,235	G	C	0.020	2.19	3.71E-06	0.19	UQCC5	52,536,605	52,579,237	intronic
rs56259931	3	52,540,349	T	C	0.020	2.19	3.71E-06	0.19	UQCC5	52,536,605	52,579,237	intronic
rs60823713	3	52,539,085	G	A	0.020	2.19	3.71E-06	0.19	UQCC5	52,536,605	52,579,237	intronic
rs60415551	3	52,538,632	T	A	0.020	2.19	3.71E-06	0.19	UQCC5	52,536,605	52,579,237	intronic

rs7895863	10	89,827,672	G	A	0.134	1.47	3.73E-06	0.19	-	-	-	intronic
rs7895851	10	89,827,644	G	A	0.134	1.47	3.73E-06	0.19	-	-	-	intronic
rs7083051	10	89,826,284	T	C	0.134	1.47	3.73E-06	0.19	-	-	-	intronic
rs7918018	10	89,825,521	C	G	0.134	1.47	3.73E-06	0.19	-	-	-	intronic
rs61220667	3	52,710,420	T	G	0.021	2.19	3.77E-06	0.19	SPCS1	52,706,106	52,711,148	3'-UTR
rs56156188	3	52,696,683	T	G	0.021	2.19	3.77E-06	0.19	GLT8D1	52,694,486	52,705,791	intronic
rs57008939	3	52,677,354	C	T	0.021	2.19	3.77E-06	0.19	PBMR1	52,545,352	52,679,714	intronic
rs72950432	3	52,662,336	T	C	0.021	2.19	3.77E-06	0.19	PBMR1	52,545,352	52,679,714	intronic
rs1442802969	3	52,661,045	A	C	0.021	2.19	3.77E-06	0.19	PBMR1	52,545,352	52,679,714	intronic
rs57757646	3	52,602,792	C	T	0.021	2.19	3.77E-06	0.19	PBMR1	52,545,352	52,679,714	intronic
rs6780005	3	52,602,599	C	G	0.021	2.19	3.77E-06	0.19	PBMR1	52,545,352	52,679,714	intronic
rs72965177	3	52,601,048	T	C	0.021	2.19	3.77E-06	0.19	PBMR1	52,545,352	52,679,714	intronic
rs12107484	3	52,598,077	T	C	0.021	2.19	3.77E-06	0.19	PBMR1	52,545,352	52,679,714	intronic
rs7647992	3	52,597,328	G	T	0.021	2.19	3.77E-06	0.19	PBMR1	52,545,352	52,679,714	intronic
rs72947589	3	52,585,530	T	A	0.021	2.19	3.77E-06	0.19	PBMR1	52,545,352	52,679,714	intronic
rs72947580	3	52,581,467	G	A	0.021	2.19	3.77E-06	0.19	PBMR1	52,545,352	52,679,714	intronic
rs7648016	3	52,580,394	T	C	0.021	2.19	3.77E-06	0.19	PBMR1	52,545,352	52,679,714	intronic
rs10510759	3	52,573,272	A	G	0.021	2.19	3.77E-06	0.19	PBMR1	52,545,352	52,679,714	intronic
rs75713246	3	52,571,964	G	A	0.021	2.19	3.77E-06	0.19	PBMR1	52,545,352	52,679,714	intronic
rs76974457	3	52,571,795	G	A	0.021	2.19	3.77E-06	0.19	PBMR1	52,545,352	52,679,714	intronic
rs143870235	5	163,545,530	G	A	0.017	2.39	3.83E-06	0.19	-	-	-	intergenic
rs72690723	8	141,780,347	G	A	0.487	1.33	3.85E-06	0.19	-	-	-	intergenic
rs41309256	15	26,940,210	C	T	0.032	1.93	3.90E-06	0.19	GABRAS	26,866,911	26,948,752	intronic
rs11185116	1	107,441,853	A	T	0.471	1.33	4.07E-06	0.2	NTNG1	107,140,007	107,481,853	intronic
rs6787028	3	34,837,931	C	T	0.332	1.36	4.10E-06	0.2	-	-	-	intergenic
rs34710884	17	45,186,544	G	A	0.034	1.89	4.20E-06	0.2	-	-	-	intergenic
rs12411671	10	20,961,387	T	C	0.058	1.71	4.22E-06	0.2	NEBL	20,780,050	21,293,011	intronic
rs12240362	10	89,827,140	C	T	0.134	1.47	4.33E-06	0.2	-	-	-	intergenic
rs6583662	10	89,826,359	G	A	0.134	1.47	4.33E-06	0.2	-	-	-	intergenic
rs2109194	12	3,482,308	C	T	0.247	0.70	4.38E-06	0.21	PRMT8	3,381,349	3,593,182	intronic
s182451717	3	52,753,087	A	G	0.022	2.16	4.53E-06	0.21	NEK4	52,708,444	52,770,940	intronic
rs192710451	3	52,753,086	G	A	0.022	2.16	4.53E-06	0.21	NEK4	52,708,444	52,770,940	intronic
rs118173502	19	9,039,503	G	A	0.016	2.42	4.53E-06	0.21	-	-	-	intergenic
-	7	130,527,721	A	C	0.046	1.79	4.55E-06	0.21	COPG2	130,506,238	130,668,748	-
-	7	130,527,712	T	C	0.046	1.79	4.55E-06	0.21	COPG2	130,506,238	130,668,748	-
-	7	130,527,710	C	T	0.046	1.79	4.55E-06	0.21	COPG2	130,506,238	130,668,748	-
rs117236985	16	54,103,811	G	A	0.018	2.42	4.65E-06	0.21	FTO	53,704,156	54,121,941	intronic
rs73156695	21	25,521,879	C	T	0.033	1.96	4.70E-06	0.21	-	-	-	intergenic
rs1243710012	12	3,482,423	A	G	0.248	0.70	4.81E-06	0.21	PRMT8	3,381,349	3,593,182	intronic
rs147580090	3	181,601,471	A	T	0.018	2.29	4.83E-06	0.21	SOX2	180,989,770	181,742,884	intronic
rs77090810	4	71,097,809	C	T	0.029	1.99	4.96E-06	0.21	SLC4A4	71,062,667	71,568,595	intronic
rs58055098	10	89,829,386	G	C	0.134	1.47	5.04E-06	0.21	-	-	-	intergenic
rs58564212	3	52,767,623	C	A	0.022	2.15	5.14E-06	0.21	NEK4	52,708,444	52,770,940	intronic

rs72960274	3	52,755,927	C	T	0.022	2.15	5.14E-06	0.21	NEK4	52,708,444	52,770,940	intronic
rs112824891	3	52,740,997	G	A	0.022	2.15	5.14E-06	0.21	NEK4	52,708,444	52,770,940	intronic
rs56276589	3	52,738,182	C	T	0.022	2.15	5.14E-06	0.21	NEK4	52,708,444	52,770,940	intronic
rs59638016	3	52,737,856	C	T	0.022	2.15	5.14E-06	0.21	NEK4	52,708,444	52,770,940	intronic
rs764636	3	52,732,681	G	A	0.022	2.15	5.14E-06	0.21	NEK4	52,708,444	52,770,940	intronic
rs113528713	3	52,727,078	G	A	0.022	2.15	5.14E-06	0.21	NEK4	52,708,444	52,770,940	intronic
rs72960237	3	52,723,637	G	A	0.022	2.15	5.14E-06	0.21	NEK4	52,708,444	52,770,940	intronic
rs57946083	3	52,721,570	A	G	0.022	2.15	5.14E-06	0.21	NEK4	52,708,444	52,770,940	intronic
rs139012364	10	44,779,527	A	G	0.017	2.20	5.14E-06	0.21	-	-	-	non-coding transcript exon
rs1373336	1	107,435,198	T	C	0.473	1.32	5.37E-06	0.22	NTNG1	107,140,007	107,481,853	intronic
rs1362062582	5	153,629,265	G	C	0.019	2.29	5.46E-06	0.22	GRIA1	153,489,615	153,706,172	intronic
rs7519783	1	200,715,929	A	G	0.256	1.37	5.59E-06	0.23	-	-	-	non-coding transcript exon
rs79717079	5	165,110,423	C	T	0.018	2.37	5.61E-06	0.23	LINC03000	164,296,956	165,171,619	intronic
rs7895530	10	89,827,592	C	T	0.174	1.41	5.73E-06	0.23	-	-	-	non-coding transcript exon
rs899419	4	56,180,940	C	T	0.027	2.01	5.80E-06	0.23	CRACD	56,049,098	56,330,609	intronic
rs11586853	1	107,432,741	C	T	0.471	1.32	6.07E-06	0.24	NTNG1	107,140,007	107,481,853	intronic
rs148578765	15	57,254,048	A	G	0.018	2.28	6.08E-06	0.24	TCF12	56,918,623	57,288,514	intronic
rs75194031	16	86,472,746	C	A	0.0.30	1.95	6.19E-06	0.24	-	-	-	intergenic
rs117696568	13	54,695,298	G	A	0.018	2.40	6.23E-06	0.24	-	-	-	intergenic
rs12068183	1	107,399,667	A	T	0.471	1.31	6.32E-06	0.24	NTNG1	107,140,007	107,481,853	intronic
rs117451992	8	37,170,414	C	A	0.142	2.60	6.35E-06	0.24	-	-	-	intergenic
rs60625973	8	4,047,798	G	C	0.016	2.47	6.40E-06	0.25	CSMD1	2,935,361	4,994,914	intronic
rs117225132	10	26,244,381	G	A	0.150	2.40	6.47E-06	0.25	GAD2	26,216,665	26,304,558	intronic
rs6793265	3	4,693,849	C	T	0.126	1.46	6.67E-06	0.25	ITPR1	4,493,345	4,847,506	intronic
rs112414294	21	38,984,927	G	A	0.027	1.94	6.68E-06	0.25	-	-	-	transcription factor binding site
rs77237825	7	117,435,203	A	G	0.021	2.18	6.73E-06	0.25	CFTR	117,287,120	117,548,675	intronic
rs73714651	7	117,434,834	A	G	0.021	2.18	6.73E-06	0.25	CFTR	117,287,120	117,548,675	intronic
rs6083264	20	23,793,665	T	C	0.036	1.86	7.30E-06	0.27	-	-	-	non-coding transcript exon
rs114221565	5	88,145,768	T	G	0.022	2.07	7.51E-06	0.28	-	-	-	regulatory region
rs143183500	5	88,143,692	T	A	0.022	2.07	7.51E-06	0.28	ENSG00000287862	87,863,703	88,144,455	intronic
rs4776284	15	66,156,559	C	A	0.103	1.51	7.53E-06	0.28	MEGF11	65,895,079	66,253,747	intronic
rs36094120	15	91,470,160	C	T	0.127	1.45	7.63E-06	0.28	CRAT37	91,408,708	91,605,873	intronic
rs78355921	1	159,972,062	G	C	0.030	1.98	7.74E-06	0.28	LINC01133	159,958,035	159,979,090	intronic
rs78059582	3	52,659,200	T	C	0.017	2.27	7.86E-06	0.28	PBRM1	52,545,352	52,679,714	intronic
rs79658570	3	52,574,513	A	G	0.017	2.27	7.86E-06	0.28	UQCC5	52,536,605	52,579,237	intronic
rs61827378	1	175,046,714	T	C	0.275	1.35	7.96E-06	0.28	-	-	-	intergenic
rs79972825	3	35,184,579	C	T	0.086	1.56	8.10E-06	0.29	-	-	-	intergenic
rs17014199	1	107,392,511	G	A	0.468	1.31	8.20E-06	0.29	NTNG1	107,140,007	107,481,853	intronic
rs72819289	17	2,922,669	G	A	0.093	1.52	8.33E-06	0.29	RAP1GAP2	2,796,438	3,037,741	intronic
rs7516036	1	200,727,825	G	A	0.250	1.36	8.40E-06	0.29	-	-	-	intergenic
rs12831259	12	72,990,904	C	A	0.025	2.06	8.43E-06	0.29	-	-	-	intronic
rs6867085	5	107,851,606	A	C	0.465	0.76	8.49E-06	0.29	-	-	-	intergenic
rs146900739	16	84,211,291	G	C	0.019	2.22	8.50E-06	0.29	-	-	-	intergenic

rs140214968	16	86,480,802	G	A	0.029	1.94	8.50E-06	0.29	FENDRR	86,474,529	86,508,860	intronic
rs10881464	1	107,391,784	T	G	0.470	1.31	8.54E-06	0.29	NTNG1	107,140,007	107,481,853	intronic
rs145146361	10	25,921,277	C	T	0.016	2.34	8.55E-06	0.29	-	-	-	intergenic
rs117482124	15	57,093,760	C	T	0.018	2.28	8.60E-06	0.29	TCF12	56,918,623	57,288,514	intronic
rs6876467	5	4,124,123	C	G	0.437	1.32	8.63E-06	0.29	-	-	-	intergenic
rs10745796	12	97,412,397	A	G	0.057	1.65	9.00E-06	0.3	-	-	-	intergenic
rs4141777	13	25,919,174	A	G	0.395	0.75	9.03E-06	0.3	ATP8A2	25,371,974	26,025,851	intronic
rs4589733	2	38,763,253	A	G	0.029	1.94	9.09E-06	0.3	GEMIN6	38,751,534	38,781,965	intronic
rs10753880	1	200,731,246	T	A	0.249	1.36	9.41E-06	0.31	-	-	-	intergenic
rs9324566	8	141,784,984	C	T	0.466	1.32	9.69E-06	0.32	-	-	-	intergenic
rs7872368	9	4,297,351	G	A	0.299	1.34	9.77E-06	0.32	GLIS3	3,824,127	4,300,068	intronic
rs11706956	3	186,355,693	T	C	0.495	1.33	9.85E-06	0.32	DGKG	186,147,201	186,362,234	intronic
rs36168950	7	130,574,654	A	C	0.078	1.57	9.99E-06	0.32	COPG2	130,506,238	130,668,748	intronic

MAF, minor allele frequency; HR, hazard ratio; FDR, false discovery rate
