# Supplementary material for: Germline Polymorphisms Associated with Overall Survival in Lung Adenocarcinoma: Genome-Wide Analysis
Source: Cancers (Basel). 2024 Sep 25;16(19):3264. doi: 10.3390/cancers16193264 (PMC11475969; doi:10.3390/cancers16193264)
Supplement: Supplementary file 1 [file cancers-16-03264-s001.zip › SupplementaryTable S2.pdf]

Supplementary Table S2. eQTLs, in Gtex database, among the 224 variants associated with survival at *P* -value < 1.0 x 10^{-5}

variant ID	SNP ID	reference allele	alternative allele	gene	gene symbol*	distance from transcription start site	slope	slope SE	*P* -value	tissue
chr1_159972062_G_C_b38	rs78355921	G	C	ENSG00000224259.6	LINC01133	10844	-0.647	0.114	2.69E-08	Esophagus_Mucosa
chr1_159972062_G_C_b38	rs78355921	G	C	ENSG00000224259.6	LINC01133	10844	-0.389	0.093	3.41E-05	Cells_Cultured_fibroblast
chr1_159972062_G_C_b38	rs78355921	G	C	ENSG00000224259.6	LINC01133	10844	-0.303	0.076	8.31E-05	Adipose_Visceral_Omentum
chr1_159972062_G_C_b38	rs78355921	G	C	ENSG00000224259.6	LINC01133	10844	-0.340	0.065	2.43E-07	Adipose_Subcutaneous
chr1_159972062_G_C_b38	rs78355921	G	C	ENSG00000273933.1	NA	-2002	0.567	0.130	1.54E-05	Skin_Sun_Exposed_Lower_leg
chr1_175046714_T_C_b38	rs61827378	T	C	ENSG00000120332.15	TNN	-21144	0.304	0.066	4.90E-06	Esophagus_Mucosa
chr1_175046714_T_C_b38	rs61827378	T	C	ENSG00000152061.23	RABGAP1L	887304	0.093	0.025	2.57E-04	Heart_Atrial_Appendage
chr1_175046714_T_C_b38	rs61827378	T	C	ENSG00000120332.15	TNN	-21144	0.350	0.078	1.11E-05	Testis
chr1_175046714_T_C_b38	rs61827378	T	C	ENSG00000152061.23	RABGAP1L	887304	0.100	0.025	5.50E-05	Heart_Left_Ventricle
chr1_175046714_T_C_b38	rs61827378	T	C	ENSG00000152061.23	RABGAP1L	887304	0.098	0.026	1.84E-04	Whole_Blood
chr1_175046714_T_C_b38	rs61827378	T	C	ENSG00000120332.15	TNN	-21144	-0.305	0.061	1.29E-06	Liver
chr1_200715929_G_A_b38	rs7519783	G	A	ENSG00000260088.1	DDX59-AS1	46422	-0.263	0.061	2.18E-05	Nerve_Tibial
chr1_200715929_G_A_b38	rs7519783	G	A	ENSG00000118197.13	DDX59	45960	0.161	0.041	9.00E-05	Thyroid
chr1_200715929_G_A_b38	rs7519783	G	A	ENSG00000260088.1	DDX59-AS1	46422	-0.221	0.056	9.47E-05	Artery_Tibial
chr1_200715929_G_A_b38	rs7519783	G	A	ENSG00000118197.13	DDX59	45960	0.269	0.063	3.71E-05	Brain_Frontal_Cortex
chr1_200715929_G_A_b38	rs7519783	G	A	ENSG00000260088.1	DDX59-AS1	46422	-0.400	0.088	8.76E-06	Pituitary
chr1_200715929_G_A_b38	rs7519783	G	A	ENSG00000118200.14	CAMSAP2	-23629	-0.146	0.034	2.75E-05	Whole_Blood
chr1_200715929_G_A_b38	rs7519783	G	A	ENSG00000118197.13	DDX59	45960	0.251	0.051	1.17E-06	Heart_Atrial_Appendage
chr1_200715929_G_A_b38	rs7519783	G	A	ENSG00000260088.1	DDX59-AS1	46422	-0.336	0.054	9.42E-10	Thyroid
chr1_200715929_G_A_b38	rs7519783	G	A	ENSG00000260088.1	DDX59-AS1	46422	-0.244	0.064	1.49E-04	Skin_Not_Sun_Exposed_Suprapubic
chr10_26244381_G_A_b38	rs117225132	G	A	ENSG00000136758.18	YME1L1	-910442	0.275	0.065	2.89E-05	Heart_Left_Ventricle
chr10_89824354_T_C_b38	rs4933160	T	C	ENSG00000232229.5	LINC00865	-5156	1.120	0.055	1.88E-58	Colon_Transverse
chr10_89824354_T_C_b38	rs4933160	T	C	ENSG00000152782.16	PANK1	178782	-0.154	0.036	2.22E-05	Colon_Transverse
chr10_89824354_T_C_b38	rs4933160	T	C	ENSG00000232229.5	LINC00865	-5156	0.810	0.071	2.44E-21	Small_Intestine_Terminal_Ileum
chr10_89824354_T_C_b38	rs4933160	T	C	ENSG00000232229.5	LINC00865	-5156	0.677	0.112	1.76E-08	Vagina
chr10_89824354_T_C_b38	rs4933160	T	C	ENSG00000232229.5	LINC00865	-5156	0.986	0.043	1.77E-78	Nerve_Tibial
chr10_89824354_T_C_b38	rs4933160	T	C	ENSG00000232229.5	LINC00865	-5156	0.861	0.073	1.61E-24	Spleen
chr10_89824354_T_C_b38	rs4933160	T	C	ENSG00000152778.8	IFIT5	409768	-0.192	0.049	9.41E-05	Esophagus_Muscularis
chr10_89824354_T_C_b38	rs4933160	T	C	ENSG00000232229.5	LINC00865	-5156	0.573	0.048	3.76E-27	Breast
chr10_89824354_T_C_b38	rs4933160	T	C	ENSG00000232229.5	LINC00865	-5156	0.644	0.055	3.52E-26	Stomach
chr10_89824354_T_C_b38	rs4933160	T	C	ENSG00000138182.14	KIF20B	122744	-0.143	0.032	1.20E-05	Testis
chr10_89824354_T_C_b38	rs4933160	T	C	ENSG00000138182.14	KIF20B	122744	-0.224	0.039	2.60E-08	Nerve_Tibial
chr10_89824354_T_C_b38	rs4933160	T	C	ENSG00000232229.5	LINC00865	-5156	0.871	0.047	4.85E-50	Esophagus_Gastroesophageal_junction
chr10_89824354_T_C_b38	rs4933160	T	C	ENSG00000232229.5	LINC00865	-5156	0.880	0.080	5.02E-19	Brain_Nucleus_Spinal_cord_cervical_c-1
chr10_89824354_T_C_b38	rs4933160	T	C	ENSG00000232229.5	LINC00865	-5156	1.156	0.044	9.32E-90	Esophagus_Muscularis
chr10_89824354_T_C_b38	rs4933160	T	C	ENSG00000232229.5	LINC00865	-5156	0.711	0.055	1.38E-34	Muscle_Skeletal
chr10_89824354_T_C_b38	rs4933160	T	C	ENSG00000232229.5	LINC00865	-5156	0.906	0.041	6.61E-76	Adipose_Subcutaneous
chr10_89824354_T_C_b38	rs4933160	T	C	ENSG00000232229.5	LINC00865	-5156	1.103	0.160	1.02E-08	Kidney
chr10_89824354_T_C_b38	rs4933160	T	C	ENSG00000232229.5	LINC00865	-5156	1.034	0.068	2.95E-35	Pituitary
chr10_89824354_T_C_b38	rs4933160	T	C	ENSG00000232229.5	LINC00865	-5156	1.192	0.046	3.88E-96	Artery_Tibial
chr10_89824354_T_C_b38	rs4933160	T	C	ENSG00000232229.5	LINC00865	-5156	0.960	0.051	2.75E-54	Artery_Aorta
chr10_89824354_T_C_b38	rs4933160	T	C	ENSG00000232229.5	LINC00865	-5156	0.189	0.039	1.60E-06	Cells_Cultured_fibroblast
chr10_89824354_T_C_b38	rs4933160	T	C	ENSG00000232229.5	LINC00865	-5156	0.905	0.093	1.41E-15	Brain_Nucleus_Substantia_nigra
chr10_89824354_T_C_b38	rs4933160	T	C	ENSG00000232229.5	LINC00865	-5156	0.766	0.049	3.81E-44	Esophagus_Mucosa
chr10_89824354_T_C_b38	rs4933160	T	C	ENSG00000232229.5	LINC00865	-5156	0.515	0.088	4.24E-08	Salivary_Gland
chr10_89824354_T_C_b38	rs4933160	T	C	ENSG00000232229.5	LINC00865	-5156	1.064	0.086	1.18E-23	Brain_Nucleus_Putamen_basal_ganglia
chr10_89824354_T_C_b38	rs4933160	T	C	ENSG00000232229.5	LINC00865	-5156	0.659	0.077	2.43E-15	Adrenal_Gland
chr10_89824354_T_C_b38	rs4933160	T	C	ENSG00000232229.5	LINC00865	-5156	1.197	0.074	2.88E-35	Brain_Caudate_basal_ganglia
chr10_89824354_T_C_b38	rs4933160	T	C	ENSG00000232229.5	LINC00865	-5156	0.912	0.067	1.10E-26	Ovary
chr10_89824354_T_C_b38	rs4933160	T	C	ENSG00000232229.5	LINC00865	-5156	0.824	0.129	2.32E-09	Brain_Cerebellar_Hemisphere

chr10_89824354_T_C_b38	rs4933160	T	C	ENSG00000232229.5	LINC00865	-5156	1.155	0.078	3.40E-27	Uterus
chr10_89824354_T_C_b38	rs4933160	T	C	ENSG00000232229.5	LINC00865	-5156	0.994	0.079	2.57E-26	Artery_Coronary
chr10_89824354_T_C_b38	rs4933160	T	C	ENSG00000232229.5	LINC00865	-5156	0.880	0.037	2.14E-83	Thyroid
chr10_89824354_T_C_b38	rs4933160	T	C	ENSG00000232229.5	LINC00865	-5156	1.267	0.068	1.49E-52	Heart_Atrial_Appendage
chr10_89824354_T_C_b38	rs4933160	T	C	ENSG00000232229.5	LINC00865	-5156	1.002	0.068	2.46E-29	Brain_Hippocampus
chr10_89824354_T_C_b38	rs4933160	T	C	ENSG00000232229.5	LINC00865	-5156	1.154	0.125	3.26E-15	Brain_Amygdala
chr10_89824354_T_C_b38	rs4933160	T	C	ENSG00000235100.3	NA	124683	-0.661	0.155	3.81E-05	Brain_Anterior_cingulate_cortex
chr10_89824354_T_C_b38	rs4933160	T	C	ENSG00000232229.5	LINC00865	-5156	1.176	0.080	4.62E-32	Brain_Cortex
chr10_89824354_T_C_b38	rs4933160	T	C	ENSG00000232229.5	LINC00865	-5156	1.202	0.110	2.14E-21	Brain_Cerebellum
chr10_89824354_T_C_b38	rs4933160	T	C	ENSG00000232229.5	LINC00865	-5156	0.714	0.048	2.22E-42	Skin_Sun_Exposed_Lower_leg
chr10_89824354_T_C_b38	rs4933160	T	C	ENSG00000232229.5	LINC00865	-5156	1.187	0.068	1.12E-35	Brain_Hypothalamus
chr10_89824354_T_C_b38	rs4933160	T	C	ENSG00000232229.5	LINC00865	-5156	0.727	0.090	2.41E-14	Pancreas
chr10_89824354_T_C_b38	rs4933160	T	C	ENSG00000232229.5	LINC00865	-5156	1.085	0.070	6.01E-32	Brain_Frontal_Cortex
chr10_89824354_T_C_b38	rs4933160	T	C	ENSG00000235100.3	NA	124683	-0.487	0.095	5.62E-07	Testis
chr10_89824354_T_C_b38	rs4933160	T	C	ENSG00000232229.5	LINC00865	-5156	1.056	0.069	3.29E-33	Brain_Nucleus_accumbens_basal_ganglia
chr10_89824354_T_C_b38	rs4933160	T	C	ENSG00000232229.5	LINC00865	-5156	0.845	0.082	4.78E-20	Prostate
chr10_89824354_T_C_b38	rs4933160	T	C	ENSG00000232229.5	LINC00865	-5156	0.808	0.038	2.20E-70	Lung
chr10_89824354_T_C_b38	rs4933160	T	C	ENSG00000232229.5	LINC00865	-5156	1.151	0.083	8.87E-27	Brain_Anterior_cingulate_cortex
chr10_89824354_T_C_b38	rs4933160	T	C	ENSG00000232229.5	LINC00865	-5156	0.922	0.073	6.05E-30	Heart_Left_Ventricle
chr10_89824354_T_C_b38	rs4933160	T	C	ENSG00000232229.5	LINC00865	-5156	0.315	0.045	2.12E-11	Testis
chr10_89824354_T_C_b38	rs4933160	T	C	ENSG00000232229.5	LINC00865	-5156	1.075	0.052	9.47E-57	Colon_Sigmoid
chr10_89824354_T_C_b38	rs4933160	T	C	ENSG00000232229.5	LINC00865	-5156	0.577	0.092	3.46E-09	Liver
chr10_89824354_T_C_b38	rs4933160	T	C	ENSG00000152782.16	PANK1	178782	0.180	0.046	1.02E-04	Cells_Cultured_fibroblast
chr10_89824354_T_C_b38	rs4933160	T	C	ENSG00000232229.5	LINC00865	-5156	0.782	0.046	2.99E-49	Adipose_Visceral_Omentum
chr10_89824354_T_C_b38	rs4933160	T	C	ENSG00000232229.5	LINC00865	-5156	0.589	0.051	4.23E-27	Skin_Not_Sun_Exposed_Suprapubic
chr10_89824354_T_C_b38	rs4933160	T	C	ENSG00000232229.5	LINC00865	-5156	0.425	0.054	1.24E-14	Whole_Blood
chr10_89824422_T_G_b38	rs4933161	T	G	ENSG00000232229.5	LINC00865	-5088	1.151	0.083	8.87E-27	Brain_Anterior_cingulate_cortex
chr10_89824422_T_G_b38	rs4933161	T	G	ENSG00000232229.5	LINC00865	-5088	0.824	0.129	2.32E-09	Brain_Cerebellar_Hemisphere
chr10_89824422_T_G_b38	rs4933161	T	G	ENSG00000232229.5	LINC00865	-5088	0.644	0.055	3.52E-26	Stomach
chr10_89824422_T_G_b38	rs4933161	T	G	ENSG00000232229.5	LINC00865	-5088	1.103	0.160	1.02E-08	Kidney
chr10_89824422_T_G_b38	rs4933161	T	G	ENSG00000152782.16	PANK1	178850	-0.154	0.036	2.22E-05	Colon_Transverse
chr10_89824422_T_G_b38	rs4933161	T	G	ENSG00000232229.5	LINC00865	-5088	0.766	0.049	3.81E-44	Esophagus_Mucosa
chr10_89824422_T_G_b38	rs4933161	T	G	ENSG00000232229.5	LINC00865	-5088	0.189	0.039	1.60E-06	Cells_Cultured_fibroblast
chr10_89824422_T_G_b38	rs4933161	T	G	ENSG00000232229.5	LINC00865	-5088	0.871	0.047	4.85E-50	Esophagus_Gastroesophageal_junction
chr10_89824422_T_G_b38	rs4933161	T	G	ENSG00000232229.5	LINC00865	-5088	1.197	0.074	2.88E-35	Brain_Caudate_basal_ganglia
chr10_89824422_T_G_b38	rs4933161	T	G	ENSG00000232229.5	LINC00865	-5088	0.880	0.080	5.02E-19	Brain_Nucleus_Spinal_cord_cervical_c-1
chr10_89824422_T_G_b38	rs4933161	T	G	ENSG00000232229.5	LINC00865	-5088	1.056	0.069	3.29E-33	Brain_Nucleus_accumbens_basal_ganglia
chr10_89824422_T_G_b38	rs4933161	T	G	ENSG00000232229.5	LINC00865	-5088	0.861	0.073	1.61E-24	Spleen
chr10_89824422_T_G_b38	rs4933161	T	G	ENSG00000232229.5	LINC00865	-5088	1.176	0.080	4.62E-32	Brain_Cortex
chr10_89824422_T_G_b38	rs4933161	T	G	ENSG00000152778.8	IFIT5	409836	-0.192	0.049	9.41E-05	Esophagus_Muscularis
chr10_89824422_T_G_b38	rs4933161	T	G	ENSG00000232229.5	LINC00865	-5088	1.002	0.068	2.46E-29	Brain_Hippocampus
chr10_89824422_T_G_b38	rs4933161	T	G	ENSG00000232229.5	LINC00865	-5088	0.880	0.037	2.14E-83	Thyroid
chr10_89824422_T_G_b38	rs4933161	T	G	ENSG00000232229.5	LINC00865	-5088	0.994	0.079	2.57E-26	Artery_Coronary
chr10_89824422_T_G_b38	rs4933161	T	G	ENSG00000232229.5	LINC00865	-5088	0.912	0.067	1.10E-26	Ovary
chr10_89824422_T_G_b38	rs4933161	T	G	ENSG00000232229.5	LINC00865	-5088	0.425	0.054	1.24E-14	Whole_Blood
chr10_89824422_T_G_b38	rs4933161	T	G	ENSG00000235100.3	NA	124751	-0.487	0.095	5.62E-07	Testis
chr10_89824422_T_G_b38	rs4933161	T	G	ENSG00000232229.5	LINC00865	-5088	0.808	0.038	2.20E-70	Lung
chr10_89824422_T_G_b38	rs4933161	T	G	ENSG00000232229.5	LINC00865	-5088	1.120	0.055	1.88E-58	Colon_Transverse
chr10_89824422_T_G_b38	rs4933161	T	G	ENSG00000232229.5	LINC00865	-5088	1.064	0.086	1.18E-23	Brain_Nucleus_Putamen_basal_ganglia
chr10_89824422_T_G_b38	rs4933161	T	G	ENSG00000232229.5	LINC00865	-5088	0.986	0.043	1.77E-78	Nerve_Tibial
chr10_89824422_T_G_b38	rs4933161	T	G	ENSG00000232229.5	LINC00865	-5088	0.922	0.073	6.05E-30	Heart_Left_Ventricle
chr10_89824422_T_G_b38	rs4933161	T	G	ENSG00000232229.5	LINC00865	-5088	1.202	0.110	2.14E-21	Brain_Cerebellum
chr10_89824422_T_G_b38	rs4933161	T	G	ENSG00000232229.5	LINC00865	-5088	0.589	0.051	4.23E-27	Skin_Not_Sun_Exposed_Suprapubic

chr10_89824422_T_G_b38	rs4933161	T	G	ENSG00000138182.14	KIF20B	122812	-0.224	0.039	2.60E-08	Nerve_Tibial
chr10_89824422_T_G_b38	rs4933161	T	G	ENSG00000232229.5	LINC00865	-5088	0.711	0.055	1.38E-34	Muscle_Skeletal
chr10_89824422_T_G_b38	rs4933161	T	G	ENSG00000232229.5	LINC00865	-5088	0.515	0.088	4.24E-08	Salivary_Gland
chr10_89824422_T_G_b38	rs4933161	T	G	ENSG00000232229.5	LINC00865	-5088	0.810	0.071	2.44E-21	Small_Intestine_Terminal_Ileum
chr10_89824422_T_G_b38	rs4933161	T	G	ENSG00000232229.5	LINC00865	-5088	0.573	0.048	3.76E-27	Breast
chr10_89824422_T_G_b38	rs4933161	T	G	ENSG00000232229.5	LINC00865	-5088	1.075	0.052	9.47E-57	Colon_Sigmoid
chr10_89824422_T_G_b38	rs4933161	T	G	ENSG00000232229.5	LINC00865	-5088	0.905	0.093	1.41E-15	Brain_Nucleus_Substantia_nigra
chr10_89824422_T_G_b38	rs4933161	T	G	ENSG00000232229.5	LINC00865	-5088	1.192	0.046	3.88E-96	Artery_Tibial
chr10_89824422_T_G_b38	rs4933161	T	G	ENSG00000232229.5	LINC00865	-5088	1.267	0.068	1.49E-52	Heart_Atrial_Appendage
chr10_89824422_T_G_b38	rs4933161	T	G	ENSG00000232229.5	LINC00865	-5088	0.659	0.077	2.43E-15	Adrenal_Gland
chr10_89824422_T_G_b38	rs4933161	T	G	ENSG00000138182.14	KIF20B	122812	-0.143	0.032	1.20E-05	Testis
chr10_89824422_T_G_b38	rs4933161	T	G	ENSG00000232229.5	LINC00865	-5088	1.156	0.044	9.32E-90	Esophagus_Muscularis
chr10_89824422_T_G_b38	rs4933161	T	G	ENSG00000232229.5	LINC00865	-5088	0.677	0.112	1.76E-08	Vagina
chr10_89824422_T_G_b38	rs4933161	T	G	ENSG00000232229.5	LINC00865	-5088	1.085	0.070	6.01E-32	Brain_Frontal_Cortex
chr10_89824422_T_G_b38	rs4933161	T	G	ENSG00000232229.5	LINC00865	-5088	1.187	0.068	1.12E-35	Brain_Hypothalamus
chr10_89824422_T_G_b38	rs4933161	T	G	ENSG00000232229.5	LINC00865	-5088	1.154	0.125	3.26E-15	Brain_Amygdala
chr10_89824422_T_G_b38	rs4933161	T	G	ENSG00000232229.5	LINC00865	-5088	0.906	0.041	6.61E-76	Adipose_Subcutaneous
chr10_89824422_T_G_b38	rs4933161	T	G	ENSG00000232229.5	LINC00865	-5088	1.034	0.068	2.95E-35	Pituitary
chr10_89824422_T_G_b38	rs4933161	T	G	ENSG00000232229.5	LINC00865	-5088	0.315	0.045	2.12E-11	Testis
chr10_89824422_T_G_b38	rs4933161	T	G	ENSG00000232229.5	LINC00865	-5088	0.782	0.046	2.99E-49	Adipose_Visceral_Omentum
chr10_89824422_T_G_b38	rs4933161	T	G	ENSG00000232229.5	LINC00865	-5088	1.155	0.078	3.40E-27	Uterus
chr10_89824422_T_G_b38	rs4933161	T	G	ENSG00000232229.5	LINC00865	-5088	0.960	0.051	2.75E-54	Artery_Aorta
chr10_89824422_T_G_b38	rs4933161	T	G	ENSG00000232229.5	LINC00865	-5088	0.727	0.090	2.41E-14	Pancreas
chr10_89824422_T_G_b38	rs4933161	T	G	ENSG00000232229.5	LINC00865	-5088	0.845	0.082	4.78E-20	Prostate
chr10_89824422_T_G_b38	rs4933161	T	G	ENSG00000232229.5	LINC00865	-5088	0.714	0.048	2.22E-42	Skin_Sun_Exposed_Lower_leg
chr10_89824422_T_G_b38	rs4933161	T	G	ENSG00000235100.3	NA	124751	-0.661	0.155	3.81E-05	Brain_Anterior_cingulate_cortex
chr10_89824422_T_G_b38	rs4933161	T	G	ENSG00000152782.16	PANK1	178850	0.180	0.046	1.02E-04	Cells_Cultured_fibroblast
chr10_89824422_T_G_b38	rs4933161	T	G	ENSG00000232229.5	LINC00865	-5088	0.577	0.092	3.46E-09	Liver
chr10_89824476_T_C_b38	rs4933514	T	C	ENSG00000232229.5	LINC00865	-5034	0.572	0.048	1.19E-27	Breast
chr10_89824476_T_C_b38	rs4933514	T	C	ENSG00000232229.5	LINC00865	-5034	0.824	0.129	2.32E-09	Brain_Cerebellar_Hemisphere
chr10_89824476_T_C_b38	rs4933514	T	C	ENSG00000232229.5	LINC00865	-5034	0.979	0.043	2.04E-78	Nerve_Tibial
chr10_89824476_T_C_b38	rs4933514	T	C	ENSG00000232229.5	LINC00865	-5034	0.863	0.047	6.30E-50	Esophagus_Gastroesophageal_junction
chr10_89824476_T_C_b38	rs4933514	T	C	ENSG00000232229.5	LINC00865	-5034	0.904	0.065	3.09E-27	Ovary
chr10_89824476_T_C_b38	rs4933514	T	C	ENSG00000232229.5	LINC00865	-5034	1.120	0.054	3.44E-59	Colon_Transverse
chr10_89824476_T_C_b38	rs4933514	T	C	ENSG00000232229.5	LINC00865	-5034	0.880	0.080	5.02E-19	Brain_Nucleus_Spinal_cord_cervical_c-1
chr10_89824476_T_C_b38	rs4933514	T	C	ENSG00000232229.5	LINC00865	-5034	0.644	0.055	3.52E-26	Stomach
chr10_89824476_T_C_b38	rs4933514	T	C	ENSG00000232229.5	LINC00865	-5034	0.861	0.073	1.61E-24	Spleen
chr10_89824476_T_C_b38	rs4933514	T	C	ENSG00000232229.5	LINC00865	-5034	0.189	0.039	1.60E-06	Cells_Cultured_fibroblast
chr10_89824476_T_C_b38	rs4933514	T	C	ENSG00000232229.5	LINC00865	-5034	0.702	0.048	3.96E-41	Skin_Sun_Exposed_Lower_leg
chr10_89824476_T_C_b38	rs4933514	T	C	ENSG00000232229.5	LINC00865	-5034	1.176	0.080	4.62E-32	Brain_Cortex
chr10_89824476_T_C_b38	rs4933514	T	C	ENSG00000232229.5	LINC00865	-5034	0.425	0.053	9.48E-15	Whole_Blood
chr10_89824476_T_C_b38	rs4933514	T	C	ENSG00000232229.5	LINC00865	-5034	0.766	0.049	3.81E-44	Esophagus_Mucosa
chr10_89824476_T_C_b38	rs4933514	T	C	ENSG00000152778.8	IFIT5	409890	-0.192	0.049	9.41E-05	Esophagus_Muscularis
chr10_89824476_T_C_b38	rs4933514	T	C	ENSG00000232229.5	LINC00865	-5034	0.509	0.086	3.44E-08	Salivary_Gland
chr10_89824476_T_C_b38	rs4933514	T	C	ENSG00000232229.5	LINC00865	-5034	1.056	0.069	3.29E-33	Brain_Nucleus_accumbens_basal_ganglia
chr10_89824476_T_C_b38	rs4933514	T	C	ENSG00000232229.5	LINC00865	-5034	0.592	0.051	1.60E-27	Skin_Not_Sun_Exposed_Suprapubic
chr10_89824476_T_C_b38	rs4933514	T	C	ENSG00000232229.5	LINC00865	-5034	1.103	0.160	1.02E-08	Kidney
chr10_89824476_T_C_b38	rs4933514	T	C	ENSG00000232229.5	LINC00865	-5034	0.808	0.038	2.20E-70	Lung
chr10_89824476_T_C_b38	rs4933514	T	C	ENSG00000232229.5	LINC00865	-5034	1.034	0.068	2.95E-35	Pituitary
chr10_89824476_T_C_b38	rs4933514	T	C	ENSG00000232229.5	LINC00865	-5034	0.845	0.082	4.78E-20	Prostate
chr10_89824476_T_C_b38	rs4933514	T	C	ENSG00000232229.5	LINC00865	-5034	1.064	0.086	1.18E-23	Brain_Nucleus_Putamen_basal_ganglia
chr10_89824476_T_C_b38	rs4933514	T	C	ENSG00000232229.5	LINC00865	-5034	0.677	0.112	1.76E-08	Vagina
chr10_89824476_T_C_b38	rs4933514	T	C	ENSG00000235100.3	NA	124805	-0.661	0.155	3.81E-05	Brain_Anterior_cingulate_cortex

chr10_89824476_T_C_b38	rs4933514	T	C	ENSG00000232229.5	LINC00865	-5034	1.197	0.074	2.88E-35	Brain_Caudate_basal_ganglia
chr10_89824476_T_C_b38	rs4933514	T	C	ENSG00000232229.5	LINC00865	-5034	1.154	0.125	3.26E-15	Brain_Amygdala
chr10_89824476_T_C_b38	rs4933514	T	C	ENSG00000232229.5	LINC00865	-5034	0.879	0.037	2.81E-84	Thyroid
chr10_89824476_T_C_b38	rs4933514	T	C	ENSG00000232229.5	LINC00865	-5034	1.243	0.067	7.40E-52	Heart_Atrial_Appendage
chr10_89824476_T_C_b38	rs4933514	T	C	ENSG00000232229.5	LINC00865	-5034	1.002	0.068	2.46E-29	Brain_Hippocampus
chr10_89824476_T_C_b38	rs4933514	T	C	ENSG00000232229.5	LINC00865	-5034	0.706	0.054	3.04E-34	Muscle_Skeletal
chr10_89824476_T_C_b38	rs4933514	T	C	ENSG00000232229.5	LINC00865	-5034	0.994	0.079	2.57E-26	Artery_Coronary
chr10_89824476_T_C_b38	rs4933514	T	C	ENSG00000232229.5	LINC00865	-5034	0.905	0.093	1.41E-15	Brain_Nucleus_Substantia_nigra
chr10_89824476_T_C_b38	rs4933514	T	C	ENSG00000152782.16	PANK1	178904	-0.150	0.036	3.25E-05	Colon_Transverse
chr10_89824476_T_C_b38	rs4933514	T	C	ENSG00000232229.5	LINC00865	-5034	0.577	0.092	3.46E-09	Liver
chr10_89824476_T_C_b38	rs4933514	T	C	ENSG00000232229.5	LINC00865	-5034	0.810	0.071	2.44E-21	Small_Intestine_Terminal_Ileum
chr10_89824476_T_C_b38	rs4933514	T	C	ENSG00000232229.5	LINC00865	-5034	1.191	0.045	1.16E-97	Artery_Tibial
chr10_89824476_T_C_b38	rs4933514	T	C	ENSG00000232229.5	LINC00865	-5034	0.901	0.041	1.98E-75	Adipose_Subcutaneous
chr10_89824476_T_C_b38	rs4933514	T	C	ENSG00000232229.5	LINC00865	-5034	0.312	0.045	2.79E-11	Testis
chr10_89824476_T_C_b38	rs4933514	T	C	ENSG00000232229.5	LINC00865	-5034	1.151	0.083	8.87E-27	Brain_Anterior_cingulate_cortex
chr10_89824476_T_C_b38	rs4933514	T	C	ENSG00000235100.3	LINC00865	124805	-0.485	0.095	5.80E-07	Testis
chr10_89824476_T_C_b38	rs4933514	T	C	ENSG00000232229.5	LINC00865	-5034	0.659	0.077	2.43E-15	Adrenal_Gland
chr10_89824476_T_C_b38	rs4933514	T	C	ENSG00000232229.5	LINC00865	-5034	0.950	0.050	5.50E-54	Artery_Aorta
chr10_89824476_T_C_b38	rs4933514	T	C	ENSG00000232229.5	LINC00865	-5034	0.776	0.046	7.23E-49	Adipose_Visceral_Omentum
chr10_89824476_T_C_b38	rs4933514	T	C	ENSG00000138182.14	KIF20B	122866	-0.225	0.039	1.68E-08	Nerve_Tibial
chr10_89824476_T_C_b38	rs4933514	T	C	ENSG00000232229.5	LINC00865	-5034	1.153	0.075	1.00E-28	Uterus
chr10_89824476_T_C_b38	rs4933514	T	C	ENSG00000232229.5	LINC00865	-5034	1.085	0.070	6.01E-32	Brain_Frontal_Cortex
chr10_89824476_T_C_b38	rs4933514	T	C	ENSG00000232229.5	LINC00865	-5034	1.187	0.068	1.12E-35	Brain_Hypothalamus
chr10_89824476_T_C_b38	rs4933514	T	C	ENSG00000232229.5	LINC00865	-5034	0.918	0.073	5.84E-30	Heart_Left_Ventricle
chr10_89824476_T_C_b38	rs4933514	T	C	ENSG00000232229.5	LINC00865	-5034	1.156	0.044	9.32E-90	Esophagus_Muscularis
chr10_89824476_T_C_b38	rs4933514	T	C	ENSG00000232229.5	LINC00865	-5034	1.075	0.052	9.47E-57	Colon_Sigmoid
chr10_89824476_T_C_b38	rs4933514	T	C	ENSG00000152782.16	PANK1	178904	0.180	0.046	1.02E-04	Cells_Cultured_fibroblast
chr10_89824476_T_C_b38	rs4933514	T	C	ENSG00000232229.5	LINC00865	-5034	1.202	0.110	2.14E-21	Brain_Cerebellum
chr10_89824476_T_C_b38	rs4933514	T	C	ENSG00000138182.14	KIF20B	122866	-0.135	0.032	3.75E-05	Testis
chr10_89824476_T_C_b38	rs4933514	T	C	ENSG00000232229.5	LINC00865	-5034	0.727	0.090	2.41E-14	Pancreas
chr10_89824818_G_A_b38	rs12265484	G	A	ENSG00000232229.5	LINC00865	-4692	1.191	0.045	1.16E-97	Artery_Tibial
chr10_89824818_G_A_b38	rs12265484	G	A	ENSG00000232229.5	LINC00865	-4692	1.120	0.054	3.44E-59	Colon_Transverse
chr10_89824818_G_A_b38	rs12265484	G	A	ENSG00000232229.5	LINC00865	-4692	0.810	0.071	2.44E-21	Small_Intestine_Terminal_Ileum
chr10_89824818_G_A_b38	rs12265484	G	A	ENSG00000232229.5	LINC00865	-4692	0.189	0.039	1.60E-06	Cells_Cultured_fibroblast
chr10_89824818_G_A_b38	rs12265484	G	A	ENSG00000232229.5	LINC00865	-4692	0.644	0.055	3.52E-26	Stomach
chr10_89824818_G_A_b38	rs12265484	G	A	ENSG00000232229.5	LINC00865	-4692	0.879	0.037	2.81E-84	Thyroid
chr10_89824818_G_A_b38	rs12265484	G	A	ENSG00000232229.5	LINC00865	-4692	0.979	0.043	2.04E-78	Nerve_Tibial
chr10_89824818_G_A_b38	rs12265484	G	A	ENSG00000232229.5	LINC00865	-4692	0.880	0.080	5.02E-19	Brain_Nucleus_Spinal_cord_cervical_c-1
chr10_89824818_G_A_b38	rs12265484	G	A	ENSG00000232229.5	LINC00865	-4692	1.103	0.160	1.02E-08	Kidney
chr10_89824818_G_A_b38	rs12265484	G	A	ENSG00000232229.5	LINC00865	-4692	0.572	0.048	1.19E-27	Breast
chr10_89824818_G_A_b38	rs12265484	G	A	ENSG00000232229.5	LINC00865	-4692	0.905	0.093	1.41E-15	Brain_Nucleus_Substantia_nigra
chr10_89824818_G_A_b38	rs12265484	G	A	ENSG00000232229.5	LINC00865	-4692	0.425	0.053	9.48E-15	Whole_Blood
chr10_89824818_G_A_b38	rs12265484	G	A	ENSG00000232229.5	LINC00865	-4692	0.659	0.077	2.43E-15	Adrenal_Gland
chr10_89824818_G_A_b38	rs12265484	G	A	ENSG00000232229.5	LINC00865	-4692	1.064	0.086	1.18E-23	Brain_Nucleus_Putamen_basal_ganglia
chr10_89824818_G_A_b38	rs12265484	G	A	ENSG00000232229.5	LINC00865	-4692	1.176	0.080	4.62E-32	Brain_Cortex
chr10_89824818_G_A_b38	rs12265484	G	A	ENSG00000232229.5	LINC00865	-4692	1.151	0.083	8.87E-27	Brain_Anterior_cingulate_cortex
chr10_89824818_G_A_b38	rs12265484	G	A	ENSG00000152778.8	IFIT5	410232	-0.192	0.049	9.41E-05	Esophagus_Muscularis
chr10_89824818_G_A_b38	rs12265484	G	A	ENSG00000232229.5	LINC00865	-4692	1.002	0.068	2.46E-29	Brain_Hippocampus
chr10_89824818_G_A_b38	rs12265484	G	A	ENSG00000232229.5	LINC00865	-4692	1.243	0.067	7.40E-52	Heart_Atrial_Appendage
chr10_89824818_G_A_b38	rs12265484	G	A	ENSG00000232229.5	LINC00865	-4692	0.845	0.082	4.78E-20	Prostate
chr10_89824818_G_A_b38	rs12265484	G	A	ENSG00000232229.5	LINC00865	-4692	0.312	0.045	2.79E-11	Testis
chr10_89824818_G_A_b38	rs12265484	G	A	ENSG00000152782.16	PANK1	179246	-0.150	0.036	3.25E-05	Colon_Transverse
chr10_89824818_G_A_b38	rs12265484	G	A	ENSG00000232229.5	LINC00865	-4692	0.918	0.073	5.84E-30	Heart_Left_Ventricle

chr10_89824818_G_A_b38	rs12265484	G	A	ENSG00000232229.5	LINC00865	-4692	1.197	0.074	2.88E-35	Brain_Caudate_basal_ganglia
chr10_89824818_G_A_b38	rs12265484	G	A	ENSG00000232229.5	LINC00865	-4692	1.056	0.069	3.29E-33	Brain_Nucleus_accumbens_basal_ganglia
chr10_89824818_G_A_b38	rs12265484	G	A	ENSG00000232229.5	LINC00865	-4692	1.187	0.068	1.12E-35	Brain_Hypothalamus
chr10_89824818_G_A_b38	rs12265484	G	A	ENSG00000232229.5	LINC00865	-4692	0.677	0.112	1.76E-08	Vagina
chr10_89824818_G_A_b38	rs12265484	G	A	ENSG00000232229.5	LINC00865	-4692	1.153	0.075	1.00E-28	Uterus
chr10_89824818_G_A_b38	rs12265484	G	A	ENSG00000232229.5	LINC00865	-4692	0.950	0.050	5.50E-54	Artery_Aorta
chr10_89824818_G_A_b38	rs12265484	G	A	ENSG00000232229.5	LINC00865	-4692	0.577	0.092	3.46E-09	Liver
chr10_89824818_G_A_b38	rs12265484	G	A	ENSG00000232229.5	LINC00865	-4692	1.154	0.125	3.26E-15	Brain_Amygdala
chr10_89824818_G_A_b38	rs12265484	G	A	ENSG00000152782.16	PANK1	179246	0.180	0.046	1.02E-04	Cells_Cultured_fibroblast
chr10_89824818_G_A_b38	rs12265484	G	A	ENSG00000235100.3	NA	125147	-0.661	0.155	3.81E-05	Brain_Anterior_cingulate_cortex
chr10_89824818_G_A_b38	rs12265484	G	A	ENSG00000232229.5	LINC00865	-4692	0.592	0.051	1.60E-27	Skin_Not_Sun_Exposed_Suprapubic
chr10_89824818_G_A_b38	rs12265484	G	A	ENSG00000232229.5	LINC00865	-4692	0.509	0.086	3.44E-08	Salivary_Gland
chr10_89824818_G_A_b38	rs12265484	G	A	ENSG00000232229.5	LINC00865	-4692	1.034	0.068	2.95E-35	Pituitary
chr10_89824818_G_A_b38	rs12265484	G	A	ENSG00000232229.5	LINC00865	-4692	0.904	0.065	3.09E-27	Ovary
chr10_89824818_G_A_b38	rs12265484	G	A	ENSG00000232229.5	LINC00865	-4692	0.824	0.129	2.32E-09	Brain_Cerebellar_Hemisphere
chr10_89824818_G_A_b38	rs12265484	G	A	ENSG00000232229.5	LINC00865	-4692	1.075	0.052	9.47E-57	Colon_Sigmoid
chr10_89824818_G_A_b38	rs12265484	G	A	ENSG00000232229.5	LINC00865	-4692	0.727	0.090	2.41E-14	Pancreas
chr10_89824818_G_A_b38	rs12265484	G	A	ENSG00000232229.5	LINC00865	-4692	1.085	0.070	6.01E-32	Brain_Frontal_Cortex
chr10_89824818_G_A_b38	rs12265484	G	A	ENSG00000232229.5	LINC00865	-4692	0.776	0.046	7.23E-49	Adipose_Visceral_Omentum
chr10_89824818_G_A_b38	rs12265484	G	A	ENSG00000232229.5	LINC00865	-4692	0.706	0.054	3.04E-34	Muscle_Skeletal
chr10_89824818_G_A_b38	rs12265484	G	A	ENSG00000232229.5	LINC00865	-4692	0.861	0.073	1.61E-24	Spleen
chr10_89824818_G_A_b38	rs12265484	G	A	ENSG00000138182.14	KIF20B	123208	-0.135	0.032	3.75E-05	Testis
chr10_89824818_G_A_b38	rs12265484	G	A	ENSG00000232229.5	LINC00865	-4692	0.901	0.041	1.98E-75	Adipose_Subcutaneous
chr10_89824818_G_A_b38	rs12265484	G	A	ENSG00000232229.5	LINC00865	-4692	0.766	0.049	3.81E-44	Esophagus_Mucosa
chr10_89824818_G_A_b38	rs12265484	G	A	ENSG00000235100.3	LINC00865	125147	-0.485	0.095	5.80E-07	Testis
chr10_89824818_G_A_b38	rs12265484	G	A	ENSG00000138182.14	KIF20B	123208	-0.225	0.039	1.68E-08	Nerve_Tibial
chr10_89824818_G_A_b38	rs12265484	G	A	ENSG00000232229.5	LINC00865	-4692	0.863	0.047	6.30E-50	Esophagus_Gastroesophageal_junction
chr10_89824818_G_A_b38	rs12265484	G	A	ENSG00000232229.5	LINC00865	-4692	0.702	0.048	3.96E-41	Skin_Sun_Exposed_Lower_leg
chr10_89824818_G_A_b38	rs12265484	G	A	ENSG00000232229.5	LINC00865	-4692	0.994	0.079	2.57E-26	Artery_Coronary
chr10_89824818_G_A_b38	rs12265484	G	A	ENSG00000232229.5	LINC00865	-4692	1.202	0.110	2.14E-21	Brain_Cerebellum
chr10_89824818_G_A_b38	rs12265484	G	A	ENSG00000232229.5	LINC00865	-4692	1.156	0.044	9.32E-90	Esophagus_Muscularis
chr10_89824818_G_A_b38	rs12265484	G	A	ENSG00000232229.5	LINC00865	-4692	0.808	0.038	2.20E-70	Lung
chr10_89826284_T_C_b38	rs7083051	T	C	ENSG00000232229.5	LINC00865	-3226	1.191	0.045	1.16E-97	Artery_Tibial
chr10_89826284_T_C_b38	rs7083051	T	C	ENSG00000232229.5	LINC00865	-3226	0.572	0.048	1.19E-27	Breast
chr10_89826284_T_C_b38	rs7083051	T	C	ENSG00000232229.5	LINC00865	-3226	1.120	0.054	3.44E-59	Colon_Transverse
chr10_89826284_T_C_b38	rs7083051	T	C	ENSG00000138182.14	KIF20B	124674	-0.135	0.032	3.75E-05	Testis
chr10_89826284_T_C_b38	rs7083051	T	C	ENSG00000232229.5	LINC00865	-3226	1.034	0.068	2.95E-35	Pituitary
chr10_89826284_T_C_b38	rs7083051	T	C	ENSG00000232229.5	LINC00865	-3226	1.176	0.080	4.62E-32	Brain_Cortex
chr10_89826284_T_C_b38	rs7083051	T	C	ENSG00000232229.5	LINC00865	-3226	1.085	0.070	6.01E-32	Brain_Frontal_Cortex
chr10_89826284_T_C_b38	rs7083051	T	C	ENSG00000232229.5	LINC00865	-3226	1.154	0.125	3.26E-15	Brain_Amygdala
chr10_89826284_T_C_b38	rs7083051	T	C	ENSG00000138182.14	KIF20B	124674	-0.225	0.039	1.68E-08	Nerve_Tibial
chr10_89826284_T_C_b38	rs7083051	T	C	ENSG00000232229.5	LINC00865	-3226	0.879	0.037	2.81E-84	Thyroid
chr10_89826284_T_C_b38	rs7083051	T	C	ENSG00000232229.5	LINC00865	-3226	1.243	0.067	7.40E-52	Heart_Atrial_Appendage
chr10_89826284_T_C_b38	rs7083051	T	C	ENSG00000152782.16	PANK1	180712	-0.150	0.036	3.25E-05	Colon_Transverse
chr10_89826284_T_C_b38	rs7083051	T	C	ENSG00000152778.8	IFIT5	411698	-0.192	0.049	9.41E-05	Esophagus_Muscularis
chr10_89826284_T_C_b38	rs7083051	T	C	ENSG00000232229.5	LINC00865	-3226	0.824	0.129	2.32E-09	Brain_Cerebellar_Hemisphere
chr10_89826284_T_C_b38	rs7083051	T	C	ENSG00000232229.5	LINC00865	-3226	1.187	0.068	1.12E-35	Brain_Hypothalamus
chr10_89826284_T_C_b38	rs7083051	T	C	ENSG00000232229.5	LINC00865	-3226	0.861	0.073	1.61E-24	Spleen
chr10_89826284_T_C_b38	rs7083051	T	C	ENSG00000232229.5	LINC00865	-3226	0.509	0.086	3.44E-08	Salivary_Gland
chr10_89826284_T_C_b38	rs7083051	T	C	ENSG00000232229.5	LINC00865	-3226	0.592	0.051	1.60E-27	Skin_Not_Sun_Exposed_Suprapubic
chr10_89826284_T_C_b38	rs7083051	T	C	ENSG00000232229.5	LINC00865	-3226	1.202	0.110	2.14E-21	Brain_Cerebellum
chr10_89826284_T_C_b38	rs7083051	T	C	ENSG00000232229.5	LINC00865	-3226	1.056	0.069	3.29E-33	Brain_Nucleus_accumbens_basal_ganglia
chr10_89826284_T_C_b38	rs7083051	T	C	ENSG00000232229.5	LINC00865	-3226	0.776	0.046	7.23E-49	Adipose_Visceral_Omentum

chr10_89826284_T_C_b38	rs7083051	T	C	ENSG00000232229.5	LINC00865	-3226	0.577	0.092	3.46E-09	Liver
chr10_89826284_T_C_b38	rs7083051	T	C	ENSG00000232229.5	LINC00865	-3226	0.810	0.071	2.44E-21	Small_Intestine_Terminal_Ileum
chr10_89826284_T_C_b38	rs7083051	T	C	ENSG00000232229.5	LINC00865	-3226	0.845	0.082	4.78E-20	Prostate
chr10_89826284_T_C_b38	rs7083051	T	C	ENSG00000232229.5	LINC00865	-3226	0.863	0.047	6.30E-50	Esophagus_Gastroesophageal_junction
chr10_89826284_T_C_b38	rs7083051	T	C	ENSG00000232229.5	LINC00865	-3226	0.905	0.093	1.41E-15	Brain_Nucleus_Substantia_nigra
chr10_89826284_T_C_b38	rs7083051	T	C	ENSG00000232229.5	LINC00865	-3226	0.659	0.077	2.43E-15	Adrenal_Gland
chr10_89826284_T_C_b38	rs7083051	T	C	ENSG00000232229.5	LINC00865	-3226	1.103	0.160	1.02E-08	Kidney
chr10_89826284_T_C_b38	rs7083051	T	C	ENSG00000232229.5	LINC00865	-3226	1.075	0.052	9.47E-57	Colon_Sigmoid
chr10_89826284_T_C_b38	rs7083051	T	C	ENSG00000232229.5	LINC00865	-3226	0.904	0.065	3.09E-27	Ovary
chr10_89826284_T_C_b38	rs7083051	T	C	ENSG00000232229.5	LINC00865	-3226	0.727	0.090	2.41E-14	Pancreas
chr10_89826284_T_C_b38	rs7083051	T	C	ENSG00000232229.5	LINC00865	-3226	1.151	0.083	8.87E-27	Brain_Anterior_cingulate_cortex
chr10_89826284_T_C_b38	rs7083051	T	C	ENSG00000232229.5	LINC00865	-3226	0.918	0.073	5.84E-30	Heart_Left_Ventricle
chr10_89826284_T_C_b38	rs7083051	T	C	ENSG00000232229.5	LINC00865	-3226	1.002	0.068	2.46E-29	Brain_Hippocampus
chr10_89826284_T_C_b38	rs7083051	T	C	ENSG00000232229.5	LINC00865	-3226	1.064	0.086	1.18E-23	Brain_Nucleus_Putamen_basal_ganglia
chr10_89826284_T_C_b38	rs7083051	T	C	ENSG00000232229.5	LINC00865	-3226	1.197	0.074	2.88E-35	Brain_Caudate_basal_ganglia
chr10_89826284_T_C_b38	rs7083051	T	C	ENSG00000232229.5	LINC00865	-3226	0.901	0.041	1.98E-75	Adipose_Subcutaneous
chr10_89826284_T_C_b38	rs7083051	T	C	ENSG00000232229.5	LINC00865	-3226	0.880	0.080	5.02E-19	Brain_Nucleus_Spinal_cord_cervical_c-1
chr10_89826284_T_C_b38	rs7083051	T	C	ENSG00000232229.5	LINC00865	-3226	0.189	0.039	1.60E-06	Cells_Cultured_fibroblast
chr10_89826284_T_C_b38	rs7083051	T	C	ENSG00000232229.5	LINC00865	-3226	0.950	0.050	5.50E-54	Artery_Aorta
chr10_89826284_T_C_b38	rs7083051	T	C	ENSG00000232229.5	LINC00865	-3226	0.706	0.054	3.04E-34	Muscle_Skeletal
chr10_89826284_T_C_b38	rs7083051	T	C	ENSG00000232229.5	LINC00865	-3226	0.644	0.055	3.52E-26	Stomach
chr10_89826284_T_C_b38	rs7083051	T	C	ENSG00000232229.5	LINC00865	-3226	0.702	0.048	3.96E-41	Skin_Sun_Exposed_Lower_leg
chr10_89826284_T_C_b38	rs7083051	T	C	ENSG00000235100.3	NA	126613	-0.485	0.095	5.80E-07	Testis
chr10_89826284_T_C_b38	rs7083051	T	C	ENSG00000152782.16	PANK1	180712	0.180	0.046	1.02E-04	Cells_Cultured_fibroblast
chr10_89826284_T_C_b38	rs7083051	T	C	ENSG00000232229.5	LINC00865	-3226	0.979	0.043	2.04E-78	Nerve_Tibial
chr10_89826284_T_C_b38	rs7083051	T	C	ENSG00000232229.5	LINC00865	-3226	0.994	0.079	2.57E-26	Artery_Coronary
chr10_89826284_T_C_b38	rs7083051	T	C	ENSG00000232229.5	LINC00865	-3226	0.766	0.049	3.81E-44	Esophagus_Mucosa
chr10_89826284_T_C_b38	rs7083051	T	C	ENSG00000232229.5	LINC00865	-3226	0.312	0.045	2.79E-11	Testis
chr10_89826284_T_C_b38	rs7083051	T	C	ENSG00000232229.5	LINC00865	-3226	1.156	0.044	9.32E-90	Esophagus_Muscularis
chr10_89826284_T_C_b38	rs7083051	T	C	ENSG00000235100.3	NA	126613	-0.661	0.155	3.81E-05	Brain_Anterior_cingulate_cortex
chr10_89826284_T_C_b38	rs7083051	T	C	ENSG00000232229.5	LINC00865	-3226	0.425	0.053	9.48E-15	Whole_Blood
chr10_89826284_T_C_b38	rs7083051	T	C	ENSG00000232229.5	LINC00865	-3226	0.677	0.112	1.76E-08	Vagina
chr10_89826284_T_C_b38	rs7083051	T	C	ENSG00000232229.5	LINC00865	-3226	0.808	0.038	2.20E-70	Lung
chr10_89826284_T_C_b38	rs7083051	T	C	ENSG00000232229.5	LINC00865	-3226	1.153	0.075	1.00E-28	Uterus
chr10_89826359_G_A_b38	rs6583662	G	A	ENSG00000232229.5	LINC00865	-3151	1.014	0.071	4.63E-32	Pituitary
chr10_89826359_G_A_b38	rs6583662	G	A	ENSG00000138182.14	KIF20B	124749	-0.229	0.040	2.50E-08	Nerve_Tibial
chr10_89826359_G_A_b38	rs6583662	G	A	ENSG00000232229.5	LINC00865	-3151	0.572	0.095	1.17E-08	Liver
chr10_89826359_G_A_b38	rs6583662	G	A	ENSG00000232229.5	LINC00865	-3151	0.972	0.162	2.38E-07	Kidney
chr10_89826359_G_A_b38	rs6583662	G	A	ENSG00000232229.5	LINC00865	-3151	0.851	0.048	1.05E-47	Esophagus_Gastroesophageal_junction
chr10_89826359_G_A_b38	rs6583662	G	A	ENSG00000232229.5	LINC00865	-3151	1.023	0.084	4.44E-25	Artery_Coronary
chr10_89826359_G_A_b38	rs6583662	G	A	ENSG00000232229.5	LINC00865	-3151	0.558	0.052	4.35E-24	Skin_Not_Sun_Exposed_Suprapubic
chr10_89826359_G_A_b38	rs6583662	G	A	ENSG00000232229.5	LINC00865	-3151	0.828	0.074	9.45E-23	Spleen
chr10_89826359_G_A_b38	rs6583662	G	A	ENSG00000232229.5	LINC00865	-3151	0.744	0.051	6.53E-39	Esophagus_Mucosa
chr10_89826359_G_A_b38	rs6583662	G	A	ENSG00000235100.3	NA	126688	-0.562	0.098	2.33E-08	Testis
chr10_89826359_G_A_b38	rs6583662	G	A	ENSG00000232229.5	LINC00865	-3151	1.233	0.069	4.67E-49	Heart_Atrial_Appendage
chr10_89826359_G_A_b38	rs6583662	G	A	ENSG00000232229.5	LINC00865	-3151	1.084	0.098	1.46E-20	Brain_Nucleus_Putamen_basal_ganglia
chr10_89826359_G_A_b38	rs6583662	G	A	ENSG00000232229.5	LINC00865	-3151	1.114	0.056	3.58E-57	Colon_Transverse
chr10_89826359_G_A_b38	rs6583662	G	A	ENSG00000232229.5	LINC00865	-3151	1.098	0.078	7.04E-30	Brain_Nucleus_accumbens_basal_ganglia
chr10_89826359_G_A_b38	rs6583662	G	A	ENSG00000232229.5	LINC00865	-3151	1.132	0.097	1.83E-23	Brain_Cortex
chr10_89826359_G_A_b38	rs6583662	G	A	ENSG00000232229.5	LINC00865	-3151	1.186	0.048	2.80E-90	Artery_Tibial
chr10_89826359_G_A_b38	rs6583662	G	A	ENSG00000232229.5	LINC00865	-3151	0.884	0.074	3.03E-27	Heart_Left_Ventricle
chr10_89826359_G_A_b38	rs6583662	G	A	ENSG00000232229.5	LINC00865	-3151	0.858	0.139	8.23E-09	Brain_Cerebellar_Hemisphere
chr10_89826359_G_A_b38	rs6583662	G	A	ENSG00000232229.5	LINC00865	-3151	1.075	0.079	5.49E-27	Brain_Frontal_Cortex

chr10_89826359_G_A_b38	rs6583662	G	A	ENSG00000232229.5	LINC00865	-3151	0.590	0.092	6.54E-10	Pancreas
chr10_89826359_G_A_b38	rs6583662	G	A	ENSG00000232229.5	LINC00865	-3151	1.002	0.084	2.67E-21	Uterus
chr10_89826359_G_A_b38	rs6583662	G	A	ENSG00000232229.5	LINC00865	-3151	0.872	0.085	9.50E-20	Prostate
chr10_89826359_G_A_b38	rs6583662	G	A	ENSG00000235100.3	LINC00865	126688	-0.265	0.069	1.24E-04	Skin_Sun_Exposed_Lower_leg
chr10_89826359_G_A_b38	rs6583662	G	A	ENSG00000232229.5	LINC00865	-3151	1.060	0.054	5.97E-54	Colon_Sigmoid
chr10_89826359_G_A_b38	rs6583662	G	A	ENSG00000232229.5	LINC00865	-3151	0.492	0.090	2.87E-07	Salivary_Gland
chr10_89826359_G_A_b38	rs6583662	G	A	ENSG00000152782.16	PANK1	180787	-0.155	0.036	2.09E-05	Colon_Transverse
chr10_89826359_G_A_b38	rs6583662	G	A	ENSG00000232229.5	LINC00865	-3151	0.912	0.051	6.77E-50	Artery_Aorta
chr10_89826359_G_A_b38	rs6583662	G	A	ENSG00000232229.5	LINC00865	-3151	0.158	0.040	9.86E-05	Cells_Cultured_fibroblast
chr10_89826359_G_A_b38	rs6583662	G	A	ENSG00000232229.5	LINC00865	-3151	1.069	0.070	3.97E-30	Brain_Hippocampus
chr10_89826359_G_A_b38	rs6583662	G	A	ENSG00000232229.5	LINC00865	-3151	1.154	0.047	8.05E-82	Esophagus_Muscularis
chr10_89826359_G_A_b38	rs6583662	G	A	ENSG00000232229.5	LINC00865	-3151	0.790	0.040	8.03E-63	Lung
chr10_89826359_G_A_b38	rs6583662	G	A	ENSG00000232229.5	LINC00865	-3151	0.867	0.070	7.72E-24	Small_Intestine_Terminal_Ileum
chr10_89826359_G_A_b38	rs6583662	G	A	ENSG00000232229.5	LINC00865	-3151	0.739	0.047	4.65E-43	Adipose_Visceral_Omentum
chr10_89826359_G_A_b38	rs6583662	G	A	ENSG00000232229.5	LINC00865	-3151	1.138	0.136	2.61E-13	Brain_Amygdala
chr10_89826359_G_A_b38	rs6583662	G	A	ENSG00000232229.5	LINC00865	-3151	1.233	0.073	2.06E-34	Brain_Hypothalamus
chr10_89826359_G_A_b38	rs6583662	G	A	ENSG00000232229.5	LINC00865	-3151	0.428	0.055	1.97E-14	Whole_Blood
chr10_89826359_G_A_b38	rs6583662	G	A	ENSG00000232229.5	LINC00865	-3151	0.845	0.040	1.33E-71	Thyroid
chr10_89826359_G_A_b38	rs6583662	G	A	ENSG00000232229.5	LINC00865	-3151	0.837	0.084	1.11E-16	Brain_Nucleus_Spinal_cord_cervical_c-1
chr10_89826359_G_A_b38	rs6583662	G	A	ENSG00000232229.5	LINC00865	-3151	1.260	0.082	5.30E-33	Brain_Caudate_basal_ganglia
chr10_89826359_G_A_b38	rs6583662	G	A	ENSG00000138182.14	KIF20B	124749	-0.135	0.034	7.70E-05	Testis
chr10_89826359_G_A_b38	rs6583662	G	A	ENSG00000232229.5	LINC00865	-3151	0.554	0.049	6.03E-25	Breast
chr10_89826359_G_A_b38	rs6583662	G	A	ENSG00000232229.5	LINC00865	-3151	0.678	0.050	1.51E-36	Skin_Sun_Exposed_Lower_leg
chr10_89826359_G_A_b38	rs6583662	G	A	ENSG00000232229.5	LINC00865	-3151	0.620	0.082	1.38E-12	Adrenal_Gland
chr10_89826359_G_A_b38	rs6583662	G	A	ENSG00000232229.5	LINC00865	-3151	0.785	0.069	3.14E-21	Ovary
chr10_89826359_G_A_b38	rs6583662	G	A	ENSG00000152778.8	IFIT5	411773	-0.189	0.048	1.08E-04	Artery_Tibial
chr10_89826359_G_A_b38	rs6583662	G	A	ENSG00000232229.5	LINC00865	-3151	0.333	0.047	7.97E-12	Testis
chr10_89826359_G_A_b38	rs6583662	G	A	ENSG00000152782.16	PANK1	180787	0.180	0.047	1.56E-04	Cells_Cultured_fibroblast
chr10_89826359_G_A_b38	rs6583662	G	A	ENSG00000232229.5	LINC00865	-3151	0.988	0.045	3.94E-74	Nerve_Tibial
chr10_89826359_G_A_b38	rs6583662	G	A	ENSG00000232229.5	LINC00865	-3151	1.263	0.120	2.84E-20	Brain_Cerebellum
chr10_89826359_G_A_b38	rs6583662	G	A	ENSG00000232229.5	LINC00865	-3151	0.618	0.057	3.14E-23	Stomach
chr10_89826359_G_A_b38	rs6583662	G	A	ENSG00000232229.5	LINC00865	-3151	0.683	0.056	5.90E-31	Muscle_Skeletal
chr10_89826359_G_A_b38	rs6583662	G	A	ENSG00000232229.5	LINC00865	-3151	1.132	0.091	2.60E-23	Brain_Anterior_cingulate_cortex
chr10_89826359_G_A_b38	rs6583662	G	A	ENSG00000232229.5	LINC00865	-3151	0.633	0.104	1.62E-08	Vagina
chr10_89826359_G_A_b38	rs6583662	G	A	ENSG00000232229.5	LINC00865	-3151	0.874	0.043	6.54E-68	Adipose_Subcutaneous
chr10_89826359_G_A_b38	rs6583662	G	A	ENSG00000232229.5	LINC00865	-3151	0.928	0.103	3.49E-14	Brain_Nucleus_Substantia_nigra
chr10_89827644_G_A_b38	rs7895851	G	A	ENSG00000232229.5	LINC00865	-1866	1.202	0.110	2.14E-21	Brain_Cerebellum
chr10_89827644_G_A_b38	rs7895851	G	A	ENSG00000232229.5	LINC00865	-1866	1.120	0.054	3.44E-59	Colon_Transverse
chr10_89827644_G_A_b38	rs7895851	G	A	ENSG00000232229.5	LINC00865	-1866	0.766	0.049	3.81E-44	Esophagus_Mucosa
chr10_89827644_G_A_b38	rs7895851	G	A	ENSG00000232229.5	LINC00865	-1866	0.312	0.045	2.79E-11	Testis
chr10_89827644_G_A_b38	rs7895851	G	A	ENSG00000232229.5	LINC00865	-1866	0.879	0.037	2.81E-84	Thyroid
chr10_89827644_G_A_b38	rs7895851	G	A	ENSG00000232229.5	LINC00865	-1866	0.702	0.048	3.96E-41	Skin_Sun_Exposed_Lower_leg
chr10_89827644_G_A_b38	rs7895851	G	A	ENSG00000232229.5	LINC00865	-1866	0.950	0.050	5.50E-54	Artery_Aorta
chr10_89827644_G_A_b38	rs7895851	G	A	ENSG00000232229.5	LINC00865	-1866	1.176	0.080	4.62E-32	Brain_Cortex
chr10_89827644_G_A_b38	rs7895851	G	A	ENSG00000232229.5	LINC00865	-1866	0.808	0.038	2.20E-70	Lung
chr10_89827644_G_A_b38	rs7895851	G	A	ENSG00000232229.5	LINC00865	-1866	0.425	0.053	9.48E-15	Whole_Blood
chr10_89827644_G_A_b38	rs7895851	G	A	ENSG00000232229.5	LINC00865	-1866	1.034	0.068	2.95E-35	Pituitary
chr10_89827644_G_A_b38	rs7895851	G	A	ENSG00000232229.5	LINC00865	-1866	0.994	0.079	2.57E-26	Artery_Coronary
chr10_89827644_G_A_b38	rs7895851	G	A	ENSG00000232229.5	LINC00865	-1866	1.103	0.160	1.02E-08	Kidney
chr10_89827644_G_A_b38	rs7895851	G	A	ENSG00000232229.5	LINC00865	-1866	1.056	0.069	3.29E-33	Brain_Nucleus_accumbens_basal_ganglia
chr10_89827644_G_A_b38	rs7895851	G	A	ENSG00000232229.5	LINC00865	-1866	0.904	0.065	3.09E-27	Ovary
chr10_89827644_G_A_b38	rs7895851	G	A	ENSG00000232229.5	LINC00865	-1866	0.659	0.077	2.43E-15	Adrenal_Gland
chr10_89827644_G_A_b38	rs7895851	G	A	ENSG00000232229.5	LINC00865	-1866	0.901	0.041	1.98E-75	Adipose_Subcutaneous

chr10_89827644_G_A_b38	rs7895851	G	A	ENSG00000232229.5	LINC00865	-1866	1.191	0.045	1.16E-97	Artery_Tibial
chr10_89827644_G_A_b38	rs7895851	G	A	ENSG00000232229.5	LINC00865	-1866	0.572	0.048	1.19E-27	Breast
chr10_89827644_G_A_b38	rs7895851	G	A	ENSG00000232229.5	LINC00865	-1866	1.156	0.044	9.32E-90	Esophagus_Muscularis
chr10_89827644_G_A_b38	rs7895851	G	A	ENSG00000232229.5	LINC00865	-1866	0.776	0.046	7.23E-49	Adipose_Visceral_Omentum
chr10_89827644_G_A_b38	rs7895851	G	A	ENSG00000232229.5	LINC00865	-1866	1.153	0.075	1.00E-28	Uterus
chr10_89827644_G_A_b38	rs7895851	G	A	ENSG00000232229.5	LINC00865	-1866	1.154	0.125	3.26E-15	Brain_Amygdala
chr10_89827644_G_A_b38	rs7895851	G	A	ENSG00000232229.5	LINC00865	-1866	0.727	0.090	2.41E-14	Pancreas
chr10_89827644_G_A_b38	rs7895851	G	A	ENSG00000232229.5	LINC00865	-1866	1.064	0.086	1.18E-23	Brain_Nucleus_Putamen_basal_ganglia
chr10_89827644_G_A_b38	rs7895851	G	A	ENSG00000232229.5	LINC00865	-1866	0.863	0.047	6.30E-50	Esophagus_Gastroesophageal_junction
chr10_89827644_G_A_b38	rs7895851	G	A	ENSG00000232229.5	LINC00865	-1866	0.509	0.086	3.44E-08	Salivary_Gland
chr10_89827644_G_A_b38	rs7895851	G	A	ENSG00000232229.5	LINC00865	-1866	1.197	0.074	2.88E-35	Brain_Caudate_basal_ganglia
chr10_89827644_G_A_b38	rs7895851	G	A	ENSG00000232229.5	LINC00865	-1866	0.592	0.051	1.60E-27	Skin_Not_Sun_Exposed_Suprapubic
chr10_89827644_G_A_b38	rs7895851	G	A	ENSG00000232229.5	LINC00865	-1866	0.979	0.043	2.04E-78	Nerve_Tibial
chr10_89827644_G_A_b38	rs7895851	G	A	ENSG00000152782.16	PANK1	182072	-0.150	0.036	3.25E-05	Colon_Transverse
chr10_89827644_G_A_b38	rs7895851	G	A	ENSG00000235100.3	LINC00865	127973	-0.661	0.155	3.81E-05	Brain_Anterior_cingulate_cortex
chr10_89827644_G_A_b38	rs7895851	G	A	ENSG00000232229.5	LINC00865	-1866	1.151	0.083	8.87E-27	Brain_Anterior_cingulate_cortex
chr10_89827644_G_A_b38	rs7895851	G	A	ENSG00000232229.5	LINC00865	-1866	1.075	0.052	9.47E-57	Colon_Sigmoid
chr10_89827644_G_A_b38	rs7895851	G	A	ENSG00000232229.5	LINC00865	-1866	1.187	0.068	1.12E-35	Brain_Hypothalamus
chr10_89827644_G_A_b38	rs7895851	G	A	ENSG00000232229.5	LINC00865	-1866	0.861	0.073	1.61E-24	Spleen
chr10_89827644_G_A_b38	rs7895851	G	A	ENSG00000138182.14	KIF20B	126034	-0.225	0.039	1.68E-08	Nerve_Tibial
chr10_89827644_G_A_b38	rs7895851	G	A	ENSG00000232229.5	LINC00865	-1866	0.824	0.129	2.32E-09	Brain_Cerebellar_Hemisphere
chr10_89827644_G_A_b38	rs7895851	G	A	ENSG00000152778.8	IFIT5	413058	-0.192	0.049	9.41E-05	Esophagus_Muscularis
chr10_89827644_G_A_b38	rs7895851	G	A	ENSG00000232229.5	LINC00865	-1866	0.189	0.039	1.60E-06	Cells_Cultured_fibroblast
chr10_89827644_G_A_b38	rs7895851	G	A	ENSG00000232229.5	LINC00865	-1866	0.706	0.054	3.04E-34	Muscle_Skeletal
chr10_89827644_G_A_b38	rs7895851	G	A	ENSG00000232229.5	LINC00865	-1866	0.905	0.093	1.41E-15	Brain_Nucleus_Substantia_nigra
chr10_89827644_G_A_b38	rs7895851	G	A	ENSG00000235100.3	LINC00865	127973	-0.485	0.095	5.80E-07	Testis
chr10_89827644_G_A_b38	rs7895851	G	A	ENSG00000232229.5	LINC00865	-1866	0.677	0.112	1.76E-08	Vagina
chr10_89827644_G_A_b38	rs7895851	G	A	ENSG00000232229.5	LINC00865	-1866	0.918	0.073	5.84E-30	Heart_Left_Ventricle
chr10_89827644_G_A_b38	rs7895851	G	A	ENSG00000232229.5	LINC00865	-1866	0.577	0.092	3.46E-09	Liver
chr10_89827644_G_A_b38	rs7895851	G	A	ENSG00000152782.16	PANK1	182072	0.180	0.046	1.02E-04	Cells_Cultured_fibroblast
chr10_89827644_G_A_b38	rs7895851	G	A	ENSG00000232229.5	LINC00865	-1866	0.644	0.055	3.52E-26	Stomach
chr10_89827644_G_A_b38	rs7895851	G	A	ENSG00000232229.5	LINC00865	-1866	1.085	0.070	6.01E-32	Brain_Frontal_Cortex
chr10_89827644_G_A_b38	rs7895851	G	A	ENSG00000232229.5	LINC00865	-1866	0.810	0.071	2.44E-21	Small_Intestine_Terminal_Ileum
chr10_89827644_G_A_b38	rs7895851	G	A	ENSG00000232229.5	LINC00865	-1866	1.243	0.067	7.40E-52	Heart_Atrial_Appendage
chr10_89827644_G_A_b38	rs7895851	G	A	ENSG00000232229.5	LINC00865	-1866	0.880	0.080	5.02E-19	Brain_Nucleus_Spinal_cord_cervical_c-1
chr10_89827644_G_A_b38	rs7895851	G	A	ENSG00000232229.5	LINC00865	-1866	1.002	0.068	2.46E-29	Brain_Hippocampus
chr10_89827644_G_A_b38	rs7895851	G	A	ENSG00000138182.14	KIF20B	126034	-0.135	0.032	3.75E-05	Testis
chr10_89827644_G_A_b38	rs7895851	G	A	ENSG00000232229.5	LINC00865	-1866	0.845	0.082	4.78E-20	Prostate
chr10_89827672_G_A_b38	rs7895863	G	A	ENSG00000232229.5	LINC00865	-1838	1.064	0.086	1.18E-23	Brain_Nucleus_Putamen_basal_ganglia
chr10_89827672_G_A_b38	rs7895863	G	A	ENSG00000232229.5	LINC00865	-1838	0.572	0.048	1.19E-27	Breast
chr10_89827672_G_A_b38	rs7895863	G	A	ENSG00000232229.5	LINC00865	-1838	0.509	0.086	3.44E-08	Salivary_Gland
chr10_89827672_G_A_b38	rs7895863	G	A	ENSG00000232229.5	LINC00865	-1838	0.994	0.079	2.57E-26	Artery_Coronary
chr10_89827672_G_A_b38	rs7895863	G	A	ENSG00000232229.5	LINC00865	-1838	0.659	0.077	2.43E-15	Adrenal_Gland
chr10_89827672_G_A_b38	rs7895863	G	A	ENSG00000232229.5	LINC00865	-1838	0.425	0.053	9.48E-15	Whole_Blood
chr10_89827672_G_A_b38	rs7895863	G	A	ENSG00000232229.5	LINC00865	-1838	1.176	0.080	4.62E-32	Brain_Cortex
chr10_89827672_G_A_b38	rs7895863	G	A	ENSG00000232229.5	LINC00865	-1838	1.056	0.069	3.29E-33	Brain_Nucleus_accumbens_basal_ganglia
chr10_89827672_G_A_b38	rs7895863	G	A	ENSG00000232229.5	LINC00865	-1838	1.002	0.068	2.46E-29	Brain_Hippocampus
chr10_89827672_G_A_b38	rs7895863	G	A	ENSG00000232229.5	LINC00865	-1838	1.153	0.075	1.00E-28	Uterus
chr10_89827672_G_A_b38	rs7895863	G	A	ENSG00000232229.5	LINC00865	-1838	0.861	0.073	1.61E-24	Spleen
chr10_89827672_G_A_b38	rs7895863	G	A	ENSG00000232229.5	LINC00865	-1838	0.880	0.080	5.02E-19	Brain_Nucleus_Spinal_cord_cervical_c-1
chr10_89827672_G_A_b38	rs7895863	G	A	ENSG00000232229.5	LINC00865	-1838	0.879	0.037	2.81E-84	Thyroid
chr10_89827672_G_A_b38	rs7895863	G	A	ENSG00000232229.5	LINC00865	-1838	0.312	0.045	2.79E-11	Testis
chr10_89827672_G_A_b38	rs7895863	G	A	ENSG00000232229.5	LINC00865	-1838	0.901	0.041	1.98E-75	Adipose_Subcutaneous

chr10_89827672_G_A_b38	rs7895863	G	A	ENSG00000232229.5	LINC00865	-1838	1.034	0.068	2.95E-35	Pituitary
chr10_89827672_G_A_b38	rs7895863	G	A	ENSG00000232229.5	LINC00865	-1838	0.189	0.039	1.60E-06	Cells_Cultured_fibroblast
chr10_89827672_G_A_b38	rs7895863	G	A	ENSG00000232229.5	LINC00865	-1838	1.156	0.044	9.32E-90	Esophagus_Muscularis
chr10_89827672_G_A_b38	rs7895863	G	A	ENSG00000232229.5	LINC00865	-1838	1.191	0.045	1.16E-97	Artery_Tibial
chr10_89827672_G_A_b38	rs7895863	G	A	ENSG00000232229.5	LINC00865	-1838	0.979	0.043	2.04E-78	Nerve_Tibial
chr10_89827672_G_A_b38	rs7895863	G	A	ENSG00000232229.5	LINC00865	-1838	0.808	0.038	2.20E-70	Lung
chr10_89827672_G_A_b38	rs7895863	G	A	ENSG00000232229.5	LINC00865	-1838	0.776	0.046	7.23E-49	Adipose_Visceral_Omentum
chr10_89827672_G_A_b38	rs7895863	G	A	ENSG00000232229.5	LINC00865	-1838	0.824	0.129	2.32E-09	Brain_Cerebellar_Hemisphere
chr10_89827672_G_A_b38	rs7895863	G	A	ENSG00000232229.5	LINC00865	-1838	1.120	0.054	3.44E-59	Colon_Transverse
chr10_89827672_G_A_b38	rs7895863	G	A	ENSG00000232229.5	LINC00865	-1838	0.727	0.090	2.41E-14	Pancreas
chr10_89827672_G_A_b38	rs7895863	G	A	ENSG00000232229.5	LINC00865	-1838	0.702	0.048	3.96E-41	Skin_Sun_Exposed_Lower_leg
chr10_89827672_G_A_b38	rs7895863	G	A	ENSG00000232229.5	LINC00865	-1838	0.706	0.054	3.04E-34	Muscle_Skeletal
chr10_89827672_G_A_b38	rs7895863	G	A	ENSG00000232229.5	LINC00865	-1838	0.592	0.051	1.60E-27	Skin_Not_Sun_Exposed_Suprapubic
chr10_89827672_G_A_b38	rs7895863	G	A	ENSG00000232229.5	LINC00865	-1838	0.863	0.047	6.30E-50	Esophagus_Gastroesophageal_junction
chr10_89827672_G_A_b38	rs7895863	G	A	ENSG00000138182.14	KIF20B	126062	-0.135	0.032	3.75E-05	Testis
chr10_89827672_G_A_b38	rs7895863	G	A	ENSG00000152782.16	PANK1	182100	-0.150	0.036	3.25E-05	Colon_Transverse
chr10_89827672_G_A_b38	rs7895863	G	A	ENSG00000232229.5	LINC00865	-1838	0.904	0.065	3.09E-27	Ovary
chr10_89827672_G_A_b38	rs7895863	G	A	ENSG00000232229.5	LINC00865	-1838	0.950	0.050	5.50E-54	Artery_Aorta
chr10_89827672_G_A_b38	rs7895863	G	A	ENSG00000232229.5	LINC00865	-1838	0.644	0.055	3.52E-26	Stomach
chr10_89827672_G_A_b38	rs7895863	G	A	ENSG00000232229.5	LINC00865	-1838	1.085	0.070	6.01E-32	Brain_Frontal_Cortex
chr10_89827672_G_A_b38	rs7895863	G	A	ENSG00000232229.5	LINC00865	-1838	1.154	0.125	3.26E-15	Brain_Amygdala
chr10_89827672_G_A_b38	rs7895863	G	A	ENSG00000232229.5	LINC00865	-1838	0.810	0.071	2.44E-21	Small_Intestine_Terminal_Ileum
chr10_89827672_G_A_b38	rs7895863	G	A	ENSG00000232229.5	LINC00865	-1838	0.845	0.082	4.78E-20	Prostate
chr10_89827672_G_A_b38	rs7895863	G	A	ENSG00000152778.8	IFIT5	413086	-0.192	0.049	9.41E-05	Esophagus_Muscularis
chr10_89827672_G_A_b38	rs7895863	G	A	ENSG00000232229.5	LINC00865	-1838	1.243	0.067	7.40E-52	Heart_Atrial_Appendage
chr10_89827672_G_A_b38	rs7895863	G	A	ENSG00000232229.5	LINC00865	-1838	0.905	0.093	1.41E-15	Brain_Nucleus_Substantia_nigra
chr10_89827672_G_A_b38	rs7895863	G	A	ENSG00000235100.3	LINC00865	128001	-0.485	0.095	5.80E-07	Testis
chr10_89827672_G_A_b38	rs7895863	G	A	ENSG00000235100.3	LINC00865	128001	-0.661	0.155	3.81E-05	Brain_Anterior_cingulate_cortex
chr10_89827672_G_A_b38	rs7895863	G	A	ENSG00000232229.5	LINC00865	-1838	0.766	0.049	3.81E-44	Esophagus_Mucosa
chr10_89827672_G_A_b38	rs7895863	G	A	ENSG00000232229.5	LINC00865	-1838	1.202	0.110	2.14E-21	Brain_Cerebellum
chr10_89827672_G_A_b38	rs7895863	G	A	ENSG00000232229.5	LINC00865	-1838	1.151	0.083	8.87E-27	Brain_Anterior_cingulate_cortex
chr10_89827672_G_A_b38	rs7895863	G	A	ENSG00000152782.16	PANK1	182100	0.180	0.046	1.02E-04	Cells_Cultured_fibroblast
chr10_89827672_G_A_b38	rs7895863	G	A	ENSG00000138182.14	KIF20B	126062	-0.225	0.039	1.68E-08	Nerve_Tibial
chr10_89827672_G_A_b38	rs7895863	G	A	ENSG00000232229.5	LINC00865	-1838	0.918	0.073	5.84E-30	Heart_Left_Ventricle
chr10_89827672_G_A_b38	rs7895863	G	A	ENSG00000232229.5	LINC00865	-1838	1.187	0.068	1.12E-35	Brain_Hypothalamus
chr10_89827672_G_A_b38	rs7895863	G	A	ENSG00000232229.5	LINC00865	-1838	1.075	0.052	9.47E-57	Colon_Sigmoid
chr10_89827672_G_A_b38	rs7895863	G	A	ENSG00000232229.5	LINC00865	-1838	0.677	0.112	1.76E-08	Vagina
chr10_89827672_G_A_b38	rs7895863	G	A	ENSG00000232229.5	LINC00865	-1838	1.197	0.074	2.88E-35	Brain_Caudate_basal_ganglia
chr10_89827672_G_A_b38	rs7895863	G	A	ENSG00000232229.5	LINC00865	-1838	1.103	0.160	1.02E-08	Kidney
chr10_89827672_G_A_b38	rs7895863	G	A	ENSG00000232229.5	LINC00865	-1838	0.577	0.092	3.46E-09	Liver
chr10_89829386_G_C_b38	rs58055098	G	C	ENSG00000232229.5	LINC00865	-124	1.268	0.120	1.81E-20	Brain_Cerebellum
chr10_89829386_G_C_b38	rs58055098	G	C	ENSG00000235100.3	LINC00865	129715	-0.262	0.069	1.70E-04	Skin_Sun_Exposed_Lower_leg
chr10_89829386_G_C_b38	rs58055098	G	C	ENSG00000232229.5	LINC00865	-124	0.621	0.056	1.68E-23	Stomach
chr10_89829386_G_C_b38	rs58055098	G	C	ENSG00000232229.5	LINC00865	-124	0.732	0.048	8.51E-42	Adipose_Visceral_Omentum
chr10_89829386_G_C_b38	rs58055098	G	C	ENSG00000232229.5	LINC00865	-124	0.633	0.104	1.62E-08	Vagina
chr10_89829386_G_C_b38	rs58055098	G	C	ENSG00000232229.5	LINC00865	-124	1.092	0.079	4.62E-29	Brain_Nucleus_accumbens_basal_ganglia
chr10_89829386_G_C_b38	rs58055098	G	C	ENSG00000232229.5	LINC00865	-124	0.517	0.092	1.22E-07	Salivary_Gland
chr10_89829386_G_C_b38	rs58055098	G	C	ENSG00000232229.5	LINC00865	-124	0.833	0.087	6.21E-16	Brain_Nucleus_Spinal_cord_cervical_c-1
chr10_89829386_G_C_b38	rs58055098	G	C	ENSG00000232229.5	LINC00865	-124	0.676	0.050	1.16E-35	Skin_Sun_Exposed_Lower_leg
chr10_89829386_G_C_b38	rs58055098	G	C	ENSG00000232229.5	LINC00865	-124	1.024	0.085	2.63E-23	Brain_Frontal_Cortex
chr10_89829386_G_C_b38	rs58055098	G	C	ENSG00000232229.5	LINC00865	-124	0.572	0.095	1.17E-08	Liver
chr10_89829386_G_C_b38	rs58055098	G	C	ENSG00000232229.5	LINC00865	-124	1.123	0.139	1.25E-12	Brain_Amygdala
chr10_89829386_G_C_b38	rs58055098	G	C	ENSG00000232229.5	LINC00865	-124	1.002	0.084	2.67E-21	Uterus

chr10_89829386_G_C_b38	rs58055098	G	C	ENSG00000232229.5	LINC00865	-124	1.055	0.073	3.38E-28	Brain_Hippocampus
chr10_89829386_G_C_b38	rs58055098	G	C	ENSG00000232229.5	LINC00865	-124	0.842	0.073	9.87E-22	Small_Intestine_Terminal_Ileum
chr10_89829386_G_C_b38	rs58055098	G	C	ENSG00000232229.5	LINC00865	-124	1.005	0.072	2.93E-31	Pituitary
chr10_89829386_G_C_b38	rs58055098	G	C	ENSG00000232229.5	LINC00865	-124	1.228	0.070	3.78E-48	Heart_Atrial_Appendage
chr10_89829386_G_C_b38	rs58055098	G	C	ENSG00000232229.5	LINC00865	-124	1.230	0.084	7.43E-31	Brain_Caudate_basal_ganglia
chr10_89829386_G_C_b38	rs58055098	G	C	ENSG00000235100.3	LINC00865	129715	-0.556	0.098	3.78E-08	Testis
chr10_89829386_G_C_b38	rs58055098	G	C	ENSG00000232229.5	LINC00865	-124	0.551	0.050	1.92E-24	Breast
chr10_89829386_G_C_b38	rs58055098	G	C	ENSG00000232229.5	LINC00865	-124	0.845	0.048	1.43E-46	Esophagus_Gastroesophageal_junction
chr10_89829386_G_C_b38	rs58055098	G	C	ENSG00000232229.5	LINC00865	-124	0.620	0.082	1.38E-12	Adrenal_Gland
chr10_89829386_G_C_b38	rs58055098	G	C	ENSG00000235100.3	NA	129715	-0.399	0.094	3.25E-05	Pituitary
chr10_89829386_G_C_b38	rs58055098	G	C	ENSG00000232229.5	LINC00865	-124	0.907	0.051	6.27E-49	Artery_Aorta
chr10_89829386_G_C_b38	rs58055098	G	C	ENSG00000232229.5	LINC00865	-124	0.974	0.046	1.31E-70	Nerve_Tibial
chr10_89829386_G_C_b38	rs58055098	G	C	ENSG00000232229.5	LINC00865	-124	0.865	0.044	4.35E-65	Adipose_Subcutaneous
chr10_89829386_G_C_b38	rs58055098	G	C	ENSG00000232229.5	LINC00865	-124	0.826	0.074	2.12E-22	Spleen
chr10_89829386_G_C_b38	rs58055098	G	C	ENSG00000232229.5	LINC00865	-124	0.872	0.085	9.50E-20	Prostate
chr10_89829386_G_C_b38	rs58055098	G	C	ENSG00000232229.5	LINC00865	-124	0.779	0.041	4.89E-60	Lung
chr10_89829386_G_C_b38	rs58055098	G	C	ENSG00000232229.5	LINC00865	-124	0.677	0.056	3.66E-30	Muscle_Skeletal
chr10_89829386_G_C_b38	rs58055098	G	C	ENSG00000232229.5	LINC00865	-124	0.556	0.052	8.06E-24	Skin_Not_Sun_Exposed_Suprapubic
chr10_89829386_G_C_b38	rs58055098	G	C	ENSG00000232229.5	LINC00865	-124	0.335	0.047	8.06E-12	Testis
chr10_89829386_G_C_b38	rs58055098	G	C	ENSG00000138182.14	KIF20B	127776	-0.140	0.034	4.26E-05	Testis
chr10_89829386_G_C_b38	rs58055098	G	C	ENSG00000232229.5	LINC00865	-124	0.743	0.052	1.32E-38	Esophagus_Mucosa
chr10_89829386_G_C_b38	rs58055098	G	C	ENSG00000232229.5	LINC00865	-124	0.785	0.069	3.14E-21	Ovary
chr10_89829386_G_C_b38	rs58055098	G	C	ENSG00000232229.5	LINC00865	-124	1.098	0.095	2.07E-21	Brain_Anterior_cingulate_cortex
chr10_89829386_G_C_b38	rs58055098	G	C	ENSG00000232229.5	LINC00865	-124	0.425	0.055	3.24E-14	Whole_Blood
chr10_89829386_G_C_b38	rs58055098	G	C	ENSG00000232229.5	LINC00865	-124	0.865	0.076	1.43E-25	Heart_Left_Ventricle
chr10_89829386_G_C_b38	rs58055098	G	C	ENSG00000232229.5	LINC00865	-124	1.148	0.047	6.89E-80	Esophagus_Muscularis
chr10_89829386_G_C_b38	rs58055098	G	C	ENSG00000232229.5	LINC00865	-124	1.198	0.078	8.49E-31	Brain_Hypothalamus
chr10_89829386_G_C_b38	rs58055098	G	C	ENSG00000232229.5	LINC00865	-124	1.046	0.055	7.12E-52	Colon_Sigmoid
chr10_89829386_G_C_b38	rs58055098	G	C	ENSG00000232229.5	LINC00865	-124	0.592	0.092	6.50E-10	Pancreas
chr10_89829386_G_C_b38	rs58055098	G	C	ENSG00000232229.5	LINC00865	-124	1.084	0.098	1.46E-20	Brain_Nucleus_Putamen_basal_ganglia
chr10_89829386_G_C_b38	rs58055098	G	C	ENSG00000232229.5	LINC00865	-124	1.023	0.084	4.44E-25	Artery_Coronary
chr10_89829386_G_C_b38	rs58055098	G	C	ENSG00000232229.5	LINC00865	-124	0.972	0.162	2.38E-07	Kidney
chr10_89829386_G_C_b38	rs58055098	G	C	ENSG00000152782.16	PANK1	183814	-0.161	0.036	1.24E-05	Colon_Transverse
chr10_89829386_G_C_b38	rs58055098	G	C	ENSG00000232229.5	LINC00865	-124	0.154	0.040	1.52E-04	Cells_Cultured_fibroblast
chr10_89829386_G_C_b38	rs58055098	G	C	ENSG00000152778.8	IFIT5	414800	-0.190	0.048	9.62E-05	Artery_Tibial
chr10_89829386_G_C_b38	rs58055098	G	C	ENSG00000232229.5	LINC00865	-124	1.186	0.048	4.10E-90	Artery_Tibial
chr10_89829386_G_C_b38	rs58055098	G	C	ENSG00000232229.5	LINC00865	-124	1.113	0.057	5.69E-55	Colon_Transverse
chr10_89829386_G_C_b38	rs58055098	G	C	ENSG00000232229.5	LINC00865	-124	1.108	0.100	1.14E-21	Brain_Cortex
chr10_89829386_G_C_b38	rs58055098	G	C	ENSG00000232229.5	LINC00865	-124	0.845	0.040	1.40E-70	Thyroid
chr10_89829386_G_C_b38	rs58055098	G	C	ENSG00000232229.5	LINC00865	-124	0.858	0.139	8.23E-09	Brain_Cerebellar_Hemisphere
chr10_89829386_G_C_b38	rs58055098	G	C	ENSG00000138182.14	KIF20B	127776	-0.218	0.041	1.25E-07	Nerve_Tibial
chr10_89829386_G_C_b38	rs58055098	G	C	ENSG00000232229.5	LINC00865	-124	0.928	0.103	3.49E-14	Brain_Nucleus_Substantia_nigra
chr2_38763253_G_A_b38	rs4589733	G	A	ENSG00000214694.10	ARHGEF33	-126627	0.563	0.106	1.78E-07	Thyroid
chr2_38763253_G_A_b38	rs4589733	G	A	ENSG00000214694.10	ARHGEF33	-126627	0.557	0.130	2.13E-05	Skin_Sun_Exposed_Lower_leg
chr20_23793665_T_C_b38	rs6083264	T	C	ENSG00000230387.2	LINC03125	605704	0.526	0.125	2.89E-05	Whole_Blood
chr3_52538632_T_A_b38	rs60415551	T	A	ENSG00000163938.16	GNL3	-142524	0.333	0.062	1.13E-07	Esophagus_Mucosa
chr3_52538632_T_A_b38	rs60415551	T	A	ENSG00000168268.10	NT5DC2	3578	0.338	0.060	3.41E-08	Whole_Blood
chr3_52538632_T_A_b38	rs60415551	T	A	ENSG00000168268.10	NT5DC2	3578	0.373	0.082	6.98E-06	Muscle_Skeletal
chr3_52538632_T_A_b38	rs60415551	T	A	ENSG00000010327.10	STAB1	43294	0.228	0.056	4.74E-05	Artery_Tibial
chr3_52538632_T_A_b38	rs60415551	T	A	ENSG00000168237.17	GLYCTK	251543	-0.326	0.085	1.34E-04	Thyroid
chr3_52538632_T_A_b38	rs60415551	T	A	ENSG00000168268.10	NT5DC2	3578	0.530	0.118	1.13E-05	Pancreas
chr3_52538632_T_A_b38	rs60415551	T	A	ENSG00000114841.17	DNAH1	222313	-0.568	0.125	1.31E-05	Brain_Cerebellar_Hemisphere
chr3_52538632_T_A_b38	rs60415551	T	A	ENSG00000168268.10	NT5DC2	3578	0.422	0.084	1.05E-06	Stomach

chr3_52538632_T_A_b38	rs60415551	T	A	ENSG00000163938.16	GNL3	-142524	0.350	0.047	3.80E-13	Skin_Not_Sun_Exposed_Suprapubic
chr3_52538632_T_A_b38	rs60415551	T	A	ENSG00000163938.16	GNL3	-142524	0.257	0.049	2.84E-07	Skin_Sun_Exposed_Lower_leg
chr3_52538632_T_A_b38	rs60415551	T	A	ENSG00000168268.10	NT5DC2	3578	0.560	0.120	5.35E-06	Colon_Sigmoid
chr3_52539085_G_A_b38	rs60823713	G	A	ENSG00000010327.10	STAB1	43747	0.229	0.057	7.03E-05	Artery_Tibial
chr3_52539085_G_A_b38	rs60823713	G	A	ENSG00000114841.17	DNAH1	222766	-0.568	0.125	1.31E-05	Brain_Cerebellar_Hemisphere
chr3_52539085_G_A_b38	rs60823713	G	A	ENSG00000163938.16	GNL3	-142071	0.278	0.050	4.60E-08	Skin_Sun_Exposed_Lower_leg
chr3_52539085_G_A_b38	rs60823713	G	A	ENSG00000168237.17	GLYCTK	251996	-0.326	0.085	1.34E-04	Thyroid
chr3_52539085_G_A_b38	rs60823713	G	A	ENSG00000168268.10	NT5DC2	4031	0.348	0.061	2.34E-08	Whole_Blood
chr3_52539085_G_A_b38	rs60823713	G	A	ENSG00000168268.10	NT5DC2	4031	0.422	0.084	1.05E-06	Stomach
chr3_52539085_G_A_b38	rs60823713	G	A	ENSG00000168268.10	NT5DC2	4031	0.377	0.084	8.47E-06	Muscle_Skeletal
chr3_52539085_G_A_b38	rs60823713	G	A	ENSG00000163938.16	GNL3	-142071	0.362	0.048	1.87E-13	Skin_Not_Sun_Exposed_Suprapubic
chr3_52539085_G_A_b38	rs60823713	G	A	ENSG00000168268.10	NT5DC2	4031	0.579	0.122	3.51E-06	Pancreas
chr3_52539085_G_A_b38	rs60823713	G	A	ENSG00000163938.16	GNL3	-142071	0.345	0.063	7.43E-08	Esophagus_Mucosa
chr3_52539085_G_A_b38	rs60823713	G	A	ENSG00000168268.10	NT5DC2	4031	0.560	0.120	5.35E-06	Colon_Sigmoid
chr3_52539996_G_C_b38	rs73837804	G	C	ENSG00000163930.9	BAP1	129646	-0.255	0.072	4.62E-04	Artery_Tibial
chr3_52539996_G_C_b38	rs73837804	G	C	ENSG00000163938.16	GNL3	-141160	0.414	0.074	3.34E-08	Skin_Not_Sun_Exposed_Suprapubic
chr3_52539996_G_C_b38	rs73837804	G	C	ENSG00000247596.8	TWF2	300739	-0.384	0.099	1.33E-04	Cells_Cultured_fibroblast
chr3_52539996_G_C_b38	rs73837804	G	C	ENSG00000114841.17	DNAH1	223677	-0.725	0.167	2.76E-05	Brain_Cerebellar_Hemisphere
chr3_52539996_G_C_b38	rs73837804	G	C	ENSG00000168268.10	NT5DC2	4942	0.489	0.126	1.10E-04	Muscle_Skeletal
chr3_52539996_G_C_b38	rs73837804	G	C	ENSG00000163938.16	GNL3	-141160	0.284	0.073	1.20E-04	Skin_Sun_Exposed_Lower_leg
chr3_52539996_G_C_b38	rs73837804	G	C	ENSG00000163938.16	GNL3	-141160	0.394	0.095	4.27E-05	Esophagus_Mucosa
chr3_52540349_T_C_b38	rs56259931	T	C	ENSG00000168268.10	NT5DC2	5295	0.583	0.120	2.23E-06	Pancreas
chr3_52540349_T_C_b38	rs56259931	T	C	ENSG00000168268.10	NT5DC2	5295	0.560	0.120	5.35E-06	Colon_Sigmoid
chr3_52540349_T_C_b38	rs56259931	T	C	ENSG00000168237.17	GLYCTK	253260	-0.313	0.084	2.23E-04	Thyroid
chr3_52540349_T_C_b38	rs56259931	T	C	ENSG00000114841.17	DNAH1	224030	-0.568	0.125	1.31E-05	Brain_Cerebellar_Hemisphere
chr3_52540349_T_C_b38	rs56259931	T	C	ENSG00000168268.10	NT5DC2	5295	0.351	0.061	1.42E-08	Whole_Blood
chr3_52540349_T_C_b38	rs56259931	T	C	ENSG00000163938.16	GNL3	-140807	0.362	0.048	1.87E-13	Skin_Not_Sun_Exposed_Suprapubic
chr3_52540349_T_C_b38	rs56259931	T	C	ENSG00000010327.10	STAB1	45011	0.233	0.057	4.62E-05	Artery_Tibial
chr3_52540349_T_C_b38	rs56259931	T	C	ENSG00000163938.16	GNL3	-140807	0.291	0.050	8.49E-09	Skin_Sun_Exposed_Lower_leg
chr3_52540349_T_C_b38	rs56259931	T	C	ENSG00000168268.10	NT5DC2	5295	0.369	0.084	1.22E-05	Muscle_Skeletal
chr3_52540349_T_C_b38	rs56259931	T	C	ENSG00000168268.10	NT5DC2	5295	0.419	0.084	1.12E-06	Stomach
chr3_52540349_T_C_b38	rs56259931	T	C	ENSG00000163938.16	GNL3	-140807	0.339	0.062	9.16E-08	Esophagus_Mucosa
chr3_52541235_G_C_b38	rs57319306	G	C	ENSG00000168268.10	NT5DC2	6181	0.419	0.084	1.12E-06	Stomach
chr3_52541235_G_C_b38	rs57319306	G	C	ENSG00000168268.10	NT5DC2	6181	0.351	0.061	1.42E-08	Whole_Blood
chr3_52541235_G_C_b38	rs57319306	G	C	ENSG00000168268.10	NT5DC2	6181	0.369	0.084	1.22E-05	Muscle_Skeletal
chr3_52541235_G_C_b38	rs57319306	G	C	ENSG00000163938.16	GNL3	-139921	0.362	0.048	1.87E-13	Skin_Not_Sun_Exposed_Suprapubic
chr3_52541235_G_C_b38	rs57319306	G	C	ENSG00000114841.17	DNAH1	224916	-0.568	0.125	1.31E-05	Brain_Cerebellar_Hemisphere
chr3_52541235_G_C_b38	rs57319306	G	C	ENSG00000168268.10	NT5DC2	6181	0.583	0.120	2.23E-06	Pancreas
chr3_52541235_G_C_b38	rs57319306	G	C	ENSG00000168268.10	NT5DC2	6181	0.560	0.120	5.35E-06	Colon_Sigmoid
chr3_52541235_G_C_b38	rs57319306	G	C	ENSG00000010327.10	STAB1	45897	0.233	0.057	4.62E-05	Artery_Tibial
chr3_52541235_G_C_b38	rs57319306	G	C	ENSG00000163938.16	GNL3	-139921	0.339	0.062	9.16E-08	Esophagus_Mucosa
chr3_52541235_G_C_b38	rs57319306	G	C	ENSG00000163938.16	GNL3	-139921	0.291	0.050	8.49E-09	Skin_Sun_Exposed_Lower_leg
chr3_52541235_G_C_b38	rs57319306	G	C	ENSG00000168237.17	GLYCTK	254146	-0.313	0.084	2.23E-04	Thyroid
chr3_52543318_G_A_b38	rs57560655	G	A	ENSG00000168268.10	NT5DC2	8264	0.560	0.120	5.35E-06	Colon_Sigmoid
chr3_52543318_G_A_b38	rs57560655	G	A	ENSG00000168268.10	NT5DC2	8264	0.348	0.061	2.34E-08	Whole_Blood
chr3_52543318_G_A_b38	rs57560655	G	A	ENSG00000168268.10	NT5DC2	8264	0.579	0.122	3.51E-06	Pancreas
chr3_52543318_G_A_b38	rs57560655	G	A	ENSG00000168268.10	NT5DC2	8264	0.377	0.084	8.47E-06	Muscle_Skeletal
chr3_52543318_G_A_b38	rs57560655	G	A	ENSG00000114841.17	DNAH1	226999	-0.568	0.125	1.31E-05	Brain_Cerebellar_Hemisphere
chr3_52543318_G_A_b38	rs57560655	G	A	ENSG00000163938.16	GNL3	-137838	0.362	0.048	1.87E-13	Skin_Not_Sun_Exposed_Suprapubic
chr3_52543318_G_A_b38	rs57560655	G	A	ENSG00000168237.17	GLYCTK	256229	-0.326	0.085	1.34E-04	Thyroid
chr3_52543318_G_A_b38	rs57560655	G	A	ENSG00000010327.10	STAB1	47980	0.229	0.057	7.03E-05	Artery_Tibial
chr3_52543318_G_A_b38	rs57560655	G	A	ENSG00000168268.10	NT5DC2	8264	0.422	0.084	1.05E-06	Stomach
chr3_52543318_G_A_b38	rs57560655	G	A	ENSG00000163938.16	GNL3	-137838	0.278	0.050	4.60E-08	Skin_Sun_Exposed_Lower_leg

chr3_52543318_G_A_b38	rs57560655	G	A	ENSG00000163938.16	GNL3	-137838	0.345	0.063	7.43E-08	Esophagus_Mucosa
chr3_52555824_T_C_b38	rs58100002	T	C	ENSG00000168268.10	NT5DC2	20770	0.366	0.082	9.98E-06	Muscle_Skeletal
chr3_52555824_T_C_b38	rs58100002	T	C	ENSG00000010327.10	STAB1	60486	0.232	0.055	3.10E-05	Artery_Tibial
chr3_52555824_T_C_b38	rs58100002	T	C	ENSG00000114841.17	DNAH1	239505	-0.568	0.125	1.31E-05	Brain_Cerebellar_Hemisphere
chr3_52555824_T_C_b38	rs58100002	T	C	ENSG00000163938.16	GNL3	-125332	0.328	0.061	1.36E-07	Esophagus_Mucosa
chr3_52555824_T_C_b38	rs58100002	T	C	ENSG00000168268.10	NT5DC2	20770	0.419	0.084	1.12E-06	Stomach
chr3_52555824_T_C_b38	rs58100002	T	C	ENSG00000163938.16	GNL3	-125332	0.270	0.049	5.95E-08	Skin_Sun_Exposed_Lower_leg
chr3_52555824_T_C_b38	rs58100002	T	C	ENSG00000168268.10	NT5DC2	20770	0.535	0.117	7.31E-06	Pancreas
chr3_52555824_T_C_b38	rs58100002	T	C	ENSG00000168237.17	GLYCTK	268735	-0.313	0.084	2.23E-04	Thyroid
chr3_52555824_T_C_b38	rs58100002	T	C	ENSG00000168268.10	NT5DC2	20770	0.341	0.060	2.10E-08	Whole_Blood
chr3_52555824_T_C_b38	rs58100002	T	C	ENSG00000163938.16	GNL3	-125332	0.350	0.047	3.80E-13	Skin_Not_Sun_Exposed_Suprapubic
chr3_52555824_T_C_b38	rs58100002	T	C	ENSG00000168268.10	NT5DC2	20770	0.560	0.120	5.35E-06	Colon_Sigmoid
chr3_52556552_T_C_b38	rs80332599	T	C	ENSG00000247596.8	TWF2	317295	-0.384	0.099	1.33E-04	Cells_Cultured_fibroblast
chr3_52556552_T_C_b38	rs80332599	T	C	ENSG00000114841.17	DNAH1	240233	-0.725	0.167	2.76E-05	Brain_Cerebellar_Hemisphere
chr3_52556552_T_C_b38	rs80332599	T	C	ENSG00000163938.16	GNL3	-124604	0.414	0.074	3.34E-08	Skin_Not_Sun_Exposed_Suprapubic
chr3_52556552_T_C_b38	rs80332599	T	C	ENSG00000163930.9	BAP1	146202	-0.255	0.072	4.62E-04	Artery_Tibial
chr3_52556552_T_C_b38	rs80332599	T	C	ENSG00000163938.16	GNL3	-124604	0.394	0.095	4.27E-05	Esophagus_Mucosa
chr3_52556552_T_C_b38	rs80332599	T	C	ENSG00000168268.10	NT5DC2	21498	0.489	0.126	1.10E-04	Muscle_Skeletal
chr3_52556552_T_C_b38	rs80332599	T	C	ENSG00000163938.16	GNL3	-124604	0.284	0.073	1.20E-04	Skin_Sun_Exposed_Lower_leg
chr3_52559198_T_C_b38	rs58725214	T	C	ENSG00000168268.10	NT5DC2	24144	0.341	0.060	2.10E-08	Whole_Blood
chr3_52559198_T_C_b38	rs58725214	T	C	ENSG00000168268.10	NT5DC2	24144	0.535	0.117	7.31E-06	Pancreas
chr3_52559198_T_C_b38	rs58725214	T	C	ENSG00000114841.17	DNAH1	242879	-0.568	0.125	1.31E-05	Brain_Cerebellar_Hemisphere
chr3_52559198_T_C_b38	rs58725214	T	C	ENSG00000168268.10	NT5DC2	24144	0.366	0.082	9.98E-06	Muscle_Skeletal
chr3_52559198_T_C_b38	rs58725214	T	C	ENSG00000168268.10	NT5DC2	24144	0.560	0.120	5.35E-06	Colon_Sigmoid
chr3_52559198_T_C_b38	rs58725214	T	C	ENSG00000010327.10	STAB1	63860	0.232	0.055	3.10E-05	Artery_Tibial
chr3_52559198_T_C_b38	rs58725214	T	C	ENSG00000163938.16	GNL3	-121958	0.328	0.061	1.36E-07	Esophagus_Mucosa
chr3_52559198_T_C_b38	rs58725214	T	C	ENSG00000163938.16	GNL3	-121958	0.350	0.047	3.80E-13	Skin_Not_Sun_Exposed_Suprapubic
chr3_52559198_T_C_b38	rs58725214	T	C	ENSG00000168237.17	GLYCTK	272109	-0.313	0.084	2.23E-04	Thyroid
chr3_52559198_T_C_b38	rs58725214	T	C	ENSG00000168268.10	NT5DC2	24144	0.419	0.084	1.12E-06	Stomach
chr3_52559198_T_C_b38	rs58725214	T	C	ENSG00000163938.16	GNL3	-121958	0.270	0.049	5.95E-08	Skin_Sun_Exposed_Lower_leg
chr3_52564343_T_C_b38	rs1987234	T	C	ENSG00000168237.17	GLYCTK	277254	-0.313	0.084	2.23E-04	Thyroid
chr3_52564343_T_C_b38	rs1987234	T	C	ENSG00000168268.10	NT5DC2	29289	0.419	0.084	1.12E-06	Stomach
chr3_52564343_T_C_b38	rs1987234	T	C	ENSG00000168268.10	NT5DC2	29289	0.535	0.117	7.31E-06	Pancreas
chr3_52564343_T_C_b38	rs1987234	T	C	ENSG00000168268.10	NT5DC2	29289	0.560	0.120	5.35E-06	Colon_Sigmoid
chr3_52564343_T_C_b38	rs1987234	T	C	ENSG00000168268.10	NT5DC2	29289	0.366	0.082	9.98E-06	Muscle_Skeletal
chr3_52564343_T_C_b38	rs1987234	T	C	ENSG00000114841.17	DNAH1	248024	-0.568	0.125	1.31E-05	Brain_Cerebellar_Hemisphere
chr3_52564343_T_C_b38	rs1987234	T	C	ENSG00000163938.16	GNL3	-116813	0.350	0.047	3.80E-13	Skin_Not_Sun_Exposed_Suprapubic
chr3_52564343_T_C_b38	rs1987234	T	C	ENSG00000010327.10	STAB1	69005	0.232	0.055	3.10E-05	Artery_Tibial
chr3_52564343_T_C_b38	rs1987234	T	C	ENSG00000168268.10	NT5DC2	29289	0.341	0.060	2.10E-08	Whole_Blood
chr3_52564343_T_C_b38	rs1987234	T	C	ENSG00000163938.16	GNL3	-116813	0.328	0.061	1.36E-07	Esophagus_Mucosa
chr3_52564343_T_C_b38	rs1987234	T	C	ENSG00000163938.16	GNL3	-116813	0.270	0.049	5.95E-08	Skin_Sun_Exposed_Lower_leg
chr3_52569345_G_C_b38	rs186146073	G	C	ENSG00000163930.9	BAP1	158995	-0.255	0.072	4.62E-04	Artery_Tibial
chr3_52569345_G_C_b38	rs186146073	G	C	ENSG00000163938.16	GNL3	-111811	0.284	0.073	1.20E-04	Skin_Sun_Exposed_Lower_leg
chr3_52569345_G_C_b38	rs186146073	G	C	ENSG00000114841.17	DNAH1	253026	-0.725	0.167	2.76E-05	Brain_Cerebellar_Hemisphere
chr3_52569345_G_C_b38	rs186146073	G	C	ENSG00000168268.10	NT5DC2	34291	0.489	0.126	1.10E-04	Muscle_Skeletal
chr3_52569345_G_C_b38	rs186146073	G	C	ENSG00000163938.16	GNL3	-111811	0.394	0.095	4.27E-05	Esophagus_Mucosa
chr3_52569345_G_C_b38	rs186146073	G	C	ENSG00000163938.16	GNL3	-111811	0.414	0.074	3.34E-08	Skin_Not_Sun_Exposed_Suprapubic
chr3_52569345_G_C_b38	rs186146073	G	C	ENSG00000247596.8	TWF2	330088	-0.384	0.099	1.33E-04	Cells_Cultured_fibroblast
chr3_52571795_G_A_b38	rs76974457	G	A	ENSG00000168268.10	NT5DC2	36741	0.560	0.120	5.35E-06	Colon_Sigmoid
chr3_52571795_G_A_b38	rs76974457	G	A	ENSG00000168268.10	NT5DC2	36741	0.419	0.084	1.12E-06	Stomach
chr3_52571795_G_A_b38	rs76974457	G	A	ENSG00000010327.10	STAB1	76457	0.232	0.055	3.10E-05	Artery_Tibial
chr3_52571795_G_A_b38	rs76974457	G	A	ENSG00000114841.17	DNAH1	255476	-0.568	0.125	1.31E-05	Brain_Cerebellar_Hemisphere
chr3_52571795_G_A_b38	rs76974457	G	A	ENSG00000168268.10	NT5DC2	36741	0.366	0.082	9.98E-06	Muscle_Skeletal

chr3_52571795_G_A_b38	rs76974457	G	A	ENSG00000168268.10	NT5DC2	36741	0.535	0.117	7.31E-06	Pancreas
chr3_52571795_G_A_b38	rs76974457	G	A	ENSG00000168237.17	GLYCTK	284706	-0.313	0.084	2.23E-04	Thyroid
chr3_52571795_G_A_b38	rs76974457	G	A	ENSG00000163938.16	GNL3	-109361	0.328	0.061	1.36E-07	Esophagus_Mucosa
chr3_52571795_G_A_b38	rs76974457	G	A	ENSG00000163938.16	GNL3	-109361	0.270	0.049	5.95E-08	Skin_Sun_Exposed_Lower_leg
chr3_52571795_G_A_b38	rs76974457	G	A	ENSG00000168268.10	NT5DC2	36741	0.341	0.060	2.10E-08	Whole_Blood
chr3_52571795_G_A_b38	rs76974457	G	A	ENSG00000163938.16	GNL3	-109361	0.350	0.047	3.80E-13	Skin_Not_Sun_Exposed_Suprapubic
chr3_52571964_G_A_b38	rs75713246	G	A	ENSG00000010327.10	STAB1	76626	0.232	0.055	3.10E-05	Artery_Tibial
chr3_52571964_G_A_b38	rs75713246	G	A	ENSG00000168237.17	GLYCTK	284875	-0.313	0.084	2.23E-04	Thyroid
chr3_52571964_G_A_b38	rs75713246	G	A	ENSG00000168268.10	NT5DC2	36910	0.419	0.084	1.12E-06	Stomach
chr3_52571964_G_A_b38	rs75713246	G	A	ENSG00000163938.16	GNL3	-109192	0.328	0.061	1.36E-07	Esophagus_Mucosa
chr3_52571964_G_A_b38	rs75713246	G	A	ENSG00000163938.16	GNL3	-109192	0.275	0.049	4.01E-08	Skin_Sun_Exposed_Lower_leg
chr3_52571964_G_A_b38	rs75713246	G	A	ENSG00000114841.17	DNAH1	255645	-0.568	0.125	1.31E-05	Brain_Cerebellar_Hemisphere
chr3_52571964_G_A_b38	rs75713246	G	A	ENSG00000163938.16	GNL3	-109192	0.339	0.047	3.34E-12	Skin_Not_Sun_Exposed_Suprapubic
chr3_52571964_G_A_b38	rs75713246	G	A	ENSG00000168268.10	NT5DC2	36910	0.560	0.120	5.35E-06	Colon_Sigmoid
chr3_52571964_G_A_b38	rs75713246	G	A	ENSG00000168268.10	NT5DC2	36910	0.360	0.083	1.53E-05	Muscle_Skeletal
chr3_52571964_G_A_b38	rs75713246	G	A	ENSG00000168268.10	NT5DC2	36910	0.535	0.117	7.31E-06	Pancreas
chr3_52571964_G_A_b38	rs75713246	G	A	ENSG00000168268.10	NT5DC2	36910	0.341	0.060	2.10E-08	Whole_Blood
chr3_52572154_T_C_b38	rs114577020	T	C	ENSG00000163938.16	GNL3	-109002	0.394	0.095	4.27E-05	Esophagus_Mucosa
chr3_52572154_T_C_b38	rs114577020	T	C	ENSG00000114841.17	DNAH1	255835	-0.777	0.179	2.64E-05	Brain_Cerebellar_Hemisphere
chr3_52572154_T_C_b38	rs114577020	T	C	ENSG00000163930.9	BAP1	161804	-0.262	0.075	4.75E-04	Artery_Tibial
chr3_52572154_T_C_b38	rs114577020	T	C	ENSG00000247596.8	TWF2	332897	-0.396	0.106	2.00E-04	Cells_Cultured_fibroblast
chr3_52572154_T_C_b38	rs114577020	T	C	ENSG00000163938.16	GNL3	-109002	0.294	0.075	1.04E-04	Skin_Sun_Exposed_Lower_leg
chr3_52572154_T_C_b38	rs114577020	T	C	ENSG00000168268.10	NT5DC2	37100	0.528	0.131	6.27E-05	Muscle_Skeletal
chr3_52572154_T_C_b38	rs114577020	T	C	ENSG00000163938.16	GNL3	-109002	0.425	0.077	5.21E-08	Skin_Not_Sun_Exposed_Suprapubic
chr3_52580394_T_C_b38	rs7648016	T	C	ENSG00000163938.16	GNL3	-100762	0.270	0.049	5.95E-08	Skin_Sun_Exposed_Lower_leg
chr3_52580394_T_C_b38	rs7648016	T	C	ENSG00000163938.16	GNL3	-100762	0.328	0.061	1.36E-07	Esophagus_Mucosa
chr3_52580394_T_C_b38	rs7648016	T	C	ENSG00000168268.10	NT5DC2	45340	0.560	0.120	5.35E-06	Colon_Sigmoid
chr3_52580394_T_C_b38	rs7648016	T	C	ENSG00000168237.17	GLYCTK	293305	-0.313	0.084	2.23E-04	Thyroid
chr3_52580394_T_C_b38	rs7648016	T	C	ENSG00000168268.10	NT5DC2	45340	0.419	0.084	1.12E-06	Stomach
chr3_52580394_T_C_b38	rs7648016	T	C	ENSG00000168268.10	NT5DC2	45340	0.366	0.082	9.98E-06	Muscle_Skeletal
chr3_52580394_T_C_b38	rs7648016	T	C	ENSG00000168268.10	NT5DC2	45340	0.341	0.060	2.10E-08	Whole_Blood
chr3_52580394_T_C_b38	rs7648016	T	C	ENSG00000010327.10	STAB1	85056	0.232	0.055	3.10E-05	Artery_Tibial
chr3_52580394_T_C_b38	rs7648016	T	C	ENSG00000168268.10	NT5DC2	45340	0.535	0.117	7.31E-06	Pancreas
chr3_52580394_T_C_b38	rs7648016	T	C	ENSG00000114841.17	DNAH1	264075	-0.568	0.125	1.31E-05	Brain_Cerebellar_Hemisphere
chr3_52580394_T_C_b38	rs7648016	T	C	ENSG00000163938.16	GNL3	-100762	0.350	0.047	3.80E-13	Skin_Not_Sun_Exposed_Suprapubic
chr3_52581467_G_A_b38	rs72947580	G	A	ENSG00000163938.16	GNL3	-99689	0.270	0.049	5.95E-08	Skin_Sun_Exposed_Lower_leg
chr3_52581467_G_A_b38	rs72947580	G	A	ENSG00000168268.10	NT5DC2	46413	0.419	0.084	1.12E-06	Stomach
chr3_52581467_G_A_b38	rs72947580	G	A	ENSG00000168268.10	NT5DC2	46413	0.535	0.117	7.31E-06	Pancreas
chr3_52581467_G_A_b38	rs72947580	G	A	ENSG00000114841.17	DNAH1	265148	-0.568	0.125	1.31E-05	Brain_Cerebellar_Hemisphere
chr3_52581467_G_A_b38	rs72947580	G	A	ENSG00000168268.10	NT5DC2	46413	0.560	0.120	5.35E-06	Colon_Sigmoid
chr3_52581467_G_A_b38	rs72947580	G	A	ENSG00000168268.10	NT5DC2	46413	0.341	0.060	2.10E-08	Whole_Blood
chr3_52581467_G_A_b38	rs72947580	G	A	ENSG00000168268.10	NT5DC2	46413	0.366	0.082	9.98E-06	Muscle_Skeletal
chr3_52581467_G_A_b38	rs72947580	G	A	ENSG00000163938.16	GNL3	-99689	0.328	0.061	1.36E-07	Esophagus_Mucosa
chr3_52581467_G_A_b38	rs72947580	G	A	ENSG00000163938.16	GNL3	-99689	0.350	0.047	3.80E-13	Skin_Not_Sun_Exposed_Suprapubic
chr3_52581467_G_A_b38	rs72947580	G	A	ENSG00000010327.10	STAB1	86129	0.232	0.055	3.10E-05	Artery_Tibial
chr3_52581467_G_A_b38	rs72947580	G	A	ENSG00000168237.17	GLYCTK	294378	-0.313	0.084	2.23E-04	Thyroid
chr3_52585530_T_A_b38	rs72947589	T	A	ENSG00000168268.10	NT5DC2	50476	0.341	0.060	2.10E-08	Whole_Blood
chr3_52585530_T_A_b38	rs72947589	T	A	ENSG00000168237.17	GLYCTK	298441	-0.313	0.084	2.23E-04	Thyroid
chr3_52585530_T_A_b38	rs72947589	T	A	ENSG00000163938.16	GNL3	-95626	0.350	0.047	3.80E-13	Skin_Not_Sun_Exposed_Suprapubic
chr3_52585530_T_A_b38	rs72947589	T	A	ENSG00000163938.16	GNL3	-95626	0.270	0.049	5.95E-08	Skin_Sun_Exposed_Lower_leg
chr3_52585530_T_A_b38	rs72947589	T	A	ENSG00000168268.10	NT5DC2	50476	0.560	0.120	5.35E-06	Colon_Sigmoid
chr3_52585530_T_A_b38	rs72947589	T	A	ENSG00000168268.10	NT5DC2	50476	0.366	0.082	9.98E-06	Muscle_Skeletal
chr3_52585530_T_A_b38	rs72947589	T	A	ENSG00000168268.10	NT5DC2	50476	0.419	0.084	1.12E-06	Stomach

chr3_52585530_T_A_b38	rs72947589	T	A	ENSG00000168268.10	NT5DC2	50476	0.535	0.117	7.31E-06	Pancreas
chr3_52585530_T_A_b38	rs72947589	T	A	ENSG00000114841.17	DNAH1	269211	-0.568	0.125	1.31E-05	Brain_Cerebellar_Hemisphere
chr3_52585530_T_A_b38	rs72947589	T	A	ENSG00000163938.16	GNL3	-95626	0.328	0.061	1.36E-07	Esophagus_Mucosa
chr3_52585530_T_A_b38	rs72947589	T	A	ENSG00000010327.10	STAB1	90192	0.232	0.055	3.10E-05	Artery_Tibial
chr3_52590493_T_G_b38	rs77711437	T	G	ENSG00000168268.10	NT5DC2	55439	0.489	0.126	1.10E-04	Muscle_Skeletal
chr3_52590493_T_G_b38	rs77711437	T	G	ENSG00000163930.9	BAP1	180143	-0.255	0.072	4.62E-04	Artery_Tibial
chr3_52590493_T_G_b38	rs77711437	T	G	ENSG00000163938.16	GNL3	-90663	0.414	0.074	3.34E-08	Skin_Not_Sun_Exposed_Suprapubic
chr3_52590493_T_G_b38	rs77711437	T	G	ENSG00000163938.16	GNL3	-90663	0.394	0.095	4.27E-05	Esophagus_Mucosa
chr3_52590493_T_G_b38	rs77711437	T	G	ENSG00000114841.17	DNAH1	274174	-0.725	0.167	2.76E-05	Brain_Cerebellar_Hemisphere
chr3_52590493_T_G_b38	rs77711437	T	G	ENSG00000163938.16	GNL3	-90663	0.284	0.073	1.20E-04	Skin_Sun_Exposed_Lower_leg
chr3_52590493_T_G_b38	rs77711437	T	G	ENSG00000247596.8	TWF2	351236	-0.384	0.099	1.33E-04	Cells_Cultured_fibroblast
chr3_52598077_T_C_b38	rs12107484	T	C	ENSG00000114841.17	DNAH1	281758	-0.568	0.125	1.31E-05	Brain_Cerebellar_Hemisphere
chr3_52598077_T_C_b38	rs12107484	T	C	ENSG00000168268.10	NT5DC2	63023	0.535	0.117	7.31E-06	Pancreas
chr3_52598077_T_C_b38	rs12107484	T	C	ENSG00000168268.10	NT5DC2	63023	0.560	0.120	5.35E-06	Colon_Sigmoid
chr3_52598077_T_C_b38	rs12107484	T	C	ENSG00000168237.17	GLYCTK	310988	-0.313	0.084	2.23E-04	Thyroid
chr3_52598077_T_C_b38	rs12107484	T	C	ENSG00000168268.10	NT5DC2	63023	0.366	0.082	9.98E-06	Muscle_Skeletal
chr3_52598077_T_C_b38	rs12107484	T	C	ENSG00000163938.16	GNL3	-83079	0.270	0.049	5.95E-08	Skin_Sun_Exposed_Lower_leg
chr3_52598077_T_C_b38	rs12107484	T	C	ENSG00000168268.10	NT5DC2	63023	0.419	0.084	1.12E-06	Stomach
chr3_52598077_T_C_b38	rs12107484	T	C	ENSG00000163938.16	GNL3	-83079	0.350	0.047	3.80E-13	Skin_Not_Sun_Exposed_Suprapubic
chr3_52598077_T_C_b38	rs12107484	T	C	ENSG00000163938.16	GNL3	-83079	0.328	0.061	1.36E-07	Esophagus_Mucosa
chr3_52598077_T_C_b38	rs12107484	T	C	ENSG00000010327.10	STAB1	102739	0.232	0.055	3.10E-05	Artery_Tibial
chr3_52598077_T_C_b38	rs12107484	T	C	ENSG00000168268.10	NT5DC2	63023	0.341	0.060	2.10E-08	Whole_Blood
chr3_52601048_T_C_b38	rs72965177	T	C	ENSG00000010327.10	STAB1	105710	0.232	0.055	3.10E-05	Artery_Tibial
chr3_52601048_T_C_b38	rs72965177	T	C	ENSG00000168237.17	GLYCTK	313959	-0.313	0.084	2.23E-04	Thyroid
chr3_52601048_T_C_b38	rs72965177	T	C	ENSG00000168268.10	NT5DC2	65994	0.341	0.060	2.10E-08	Whole_Blood
chr3_52601048_T_C_b38	rs72965177	T	C	ENSG00000168268.10	NT5DC2	65994	0.560	0.120	5.35E-06	Colon_Sigmoid
chr3_52601048_T_C_b38	rs72965177	T	C	ENSG00000168268.10	NT5DC2	65994	0.366	0.082	9.98E-06	Muscle_Skeletal
chr3_52601048_T_C_b38	rs72965177	T	C	ENSG00000163938.16	GNL3	-80108	0.270	0.049	5.95E-08	Skin_Sun_Exposed_Lower_leg
chr3_52601048_T_C_b38	rs72965177	T	C	ENSG00000168268.10	NT5DC2	65994	0.419	0.084	1.12E-06	Stomach
chr3_52601048_T_C_b38	rs72965177	T	C	ENSG00000163938.16	GNL3	-80108	0.328	0.061	1.36E-07	Esophagus_Mucosa
chr3_52601048_T_C_b38	rs72965177	T	C	ENSG00000168268.10	NT5DC2	65994	0.535	0.117	7.31E-06	Pancreas
chr3_52601048_T_C_b38	rs72965177	T	C	ENSG00000163938.16	GNL3	-80108	0.350	0.047	3.80E-13	Skin_Not_Sun_Exposed_Suprapubic
chr3_52601048_T_C_b38	rs72965177	T	C	ENSG00000114841.17	DNAH1	284729	-0.568	0.125	1.31E-05	Brain_Cerebellar_Hemisphere
chr3_52606100_G_A_b38	rs78735727	G	A	ENSG00000168268.10	NT5DC2	71046	0.489	0.126	1.10E-04	Muscle_Skeletal
chr3_52606100_G_A_b38	rs78735727	G	A	ENSG00000163938.16	GNL3	-75056	0.414	0.074	3.34E-08	Skin_Not_Sun_Exposed_Suprapubic
chr3_52606100_G_A_b38	rs78735727	G	A	ENSG00000114841.17	DNAH1	289781	-0.725	0.167	2.76E-05	Brain_Cerebellar_Hemisphere
chr3_52606100_G_A_b38	rs78735727	G	A	ENSG00000163938.16	GNL3	-75056	0.284	0.073	1.20E-04	Skin_Sun_Exposed_Lower_leg
chr3_52606100_G_A_b38	rs78735727	G	A	ENSG00000163938.16	GNL3	-75056	0.394	0.095	4.27E-05	Esophagus_Mucosa
chr3_52606100_G_A_b38	rs78735727	G	A	ENSG00000247596.8	TWF2	366843	-0.384	0.099	1.33E-04	Cells_Cultured_fibroblast
chr3_52606100_G_A_b38	rs78735727	G	A	ENSG00000163930.9	BAP1	195750	-0.255	0.072	4.62E-04	Artery_Tibial
chr3_52641565_T_C_b38	rs60040519	T	C	ENSG00000163938.16	GNL3	-39591	0.414	0.074	3.34E-08	Skin_Not_Sun_Exposed_Suprapubic
chr3_52641565_T_C_b38	rs60040519	T	C	ENSG00000168268.10	NT5DC2	106511	0.489	0.126	1.10E-04	Muscle_Skeletal
chr3_52641565_T_C_b38	rs60040519	T	C	ENSG00000247596.8	TWF2	402308	-0.384	0.099	1.33E-04	Cells_Cultured_fibroblast
chr3_52641565_T_C_b38	rs60040519	T	C	ENSG00000163938.16	GNL3	-39591	0.394	0.095	4.27E-05	Esophagus_Mucosa
chr3_52641565_T_C_b38	rs60040519	T	C	ENSG00000163930.9	BAP1	231215	-0.255	0.072	4.62E-04	Artery_Tibial
chr3_52641565_T_C_b38	rs60040519	T	C	ENSG00000163938.16	GNL3	-39591	0.284	0.073	1.20E-04	Skin_Sun_Exposed_Lower_leg
chr3_52641565_T_C_b38	rs60040519	T	C	ENSG00000114841.17	DNAH1	325246	-0.725	0.167	2.76E-05	Brain_Cerebellar_Hemisphere
chr3_52659200_T_C_b38	rs78059582	T	C	ENSG00000163938.16	GNL3	-21956	0.449	0.109	4.40E-05	Esophagus_Mucosa
chr3_52659200_T_C_b38	rs78059582	T	C	ENSG00000168268.10	NT5DC2	124146	0.536	0.138	1.14E-04	Muscle_Skeletal
chr3_52659200_T_C_b38	rs78059582	T	C	ENSG00000163938.16	GNL3	-21956	0.425	0.083	4.41E-07	Skin_Not_Sun_Exposed_Suprapubic
chr3_52659200_T_C_b38	rs78059582	T	C	ENSG00000247596.8	TWF2	419943	-0.384	0.099	1.33E-04	Cells_Cultured_fibroblast
chr3_52659200_T_C_b38	rs78059582	T	C	ENSG00000114841.17	DNAH1	342881	-0.725	0.167	2.76E-05	Brain_Cerebellar_Hemisphere
chr3_52662336_T_C_b38	rs72950432	T	C	ENSG00000168268.10	NT5DC2	127282	0.560	0.120	5.35E-06	Colon_Sigmoid

chr3_52662336_T_C_b38	rs72950432	T	C	ENSG00000168268.10	NT5DC2	127282	0.341	0.060	2.10E-08	Whole_Blood
chr3_52662336_T_C_b38	rs72950432	T	C	ENSG00000168268.10	NT5DC2	127282	0.535	0.117	7.31E-06	Pancreas
chr3_52662336_T_C_b38	rs72950432	T	C	ENSG00000010327.10	STAB1	166998	0.232	0.055	3.10E-05	Artery_Tibial
chr3_52662336_T_C_b38	rs72950432	T	C	ENSG00000114841.17	DNAH1	346017	-0.568	0.125	1.31E-05	Brain_Cerebellar_Hemisphere
chr3_52662336_T_C_b38	rs72950432	T	C	ENSG00000163938.16	GNL3	-18820	0.270	0.049	5.95E-08	Skin_Sun_Exposed_Lower_leg
chr3_52662336_T_C_b38	rs72950432	T	C	ENSG00000168237.17	GLYCTK	375247	-0.313	0.084	2.23E-04	Thyroid
chr3_52662336_T_C_b38	rs72950432	T	C	ENSG00000163938.16	GNL3	-18820	0.350	0.047	3.80E-13	Skin_Not_Sun_Exposed_Suprapubic
chr3_52662336_T_C_b38	rs72950432	T	C	ENSG00000163938.16	GNL3	-18820	0.328	0.061	1.36E-07	Esophagus_Mucosa
chr3_52662336_T_C_b38	rs72950432	T	C	ENSG00000168268.10	NT5DC2	127282	0.366	0.082	9.98E-06	Muscle_Skeletal
chr3_52662336_T_C_b38	rs72950432	T	C	ENSG00000168268.10	NT5DC2	127282	0.419	0.084	1.12E-06	Stomach
chr3_52696683_T_G_b38	rs56156188	T	G	ENSG00000114841.17	DNAH1	380364	-0.568	0.125	1.31E-05	Brain_Cerebellar_Hemisphere
chr3_52696683_T_G_b38	rs56156188	T	G	ENSG00000168268.10	NT5DC2	161629	0.535	0.117	7.31E-06	Pancreas
chr3_52696683_T_G_b38	rs56156188	T	G	ENSG00000163938.16	GNL3	15527	0.328	0.061	1.36E-07	Esophagus_Mucosa
chr3_52696683_T_G_b38	rs56156188	T	G	ENSG00000168268.10	NT5DC2	161629	0.560	0.120	5.35E-06	Colon_Sigmoid
chr3_52696683_T_G_b38	rs56156188	T	G	ENSG00000168237.17	GLYCTK	409594	-0.313	0.084	2.23E-04	Thyroid
chr3_52696683_T_G_b38	rs56156188	T	G	ENSG00000010327.10	STAB1	201345	0.232	0.055	3.10E-05	Artery_Tibial
chr3_52696683_T_G_b38	rs56156188	T	G	ENSG00000168268.10	NT5DC2	161629	0.341	0.060	2.10E-08	Whole_Blood
chr3_52696683_T_G_b38	rs56156188	T	G	ENSG00000168268.10	NT5DC2	161629	0.419	0.084	1.12E-06	Stomach
chr3_52696683_T_G_b38	rs56156188	T	G	ENSG00000163938.16	GNL3	15527	0.270	0.049	5.95E-08	Skin_Sun_Exposed_Lower_leg
chr3_52696683_T_G_b38	rs56156188	T	G	ENSG00000168268.10	NT5DC2	161629	0.366	0.082	9.98E-06	Muscle_Skeletal
chr3_52696683_T_G_b38	rs56156188	T	G	ENSG00000163938.16	GNL3	15527	0.350	0.047	3.80E-13	Skin_Not_Sun_Exposed_Suprapubic
chr3_52702426_G_A_b38	rs79705974	G	A	ENSG00000247596.8	TWF2	463169	-0.384	0.099	1.33E-04	Cells_Cultured_fibroblast
chr3_52702426_G_A_b38	rs79705974	G	A	ENSG00000163930.9	BAP1	292076	-0.255	0.072	4.62E-04	Artery_Tibial
chr3_52702426_G_A_b38	rs79705974	G	A	ENSG00000163938.16	GNL3	21270	0.394	0.095	4.27E-05	Esophagus_Mucosa
chr3_52702426_G_A_b38	rs79705974	G	A	ENSG00000163938.16	GNL3	21270	0.414	0.074	3.34E-08	Skin_Not_Sun_Exposed_Suprapubic
chr3_52702426_G_A_b38	rs79705974	G	A	ENSG00000114841.17	DNAH1	386107	-0.725	0.167	2.76E-05	Brain_Cerebellar_Hemisphere
chr3_52702426_G_A_b38	rs79705974	G	A	ENSG00000163938.16	GNL3	21270	0.284	0.073	1.20E-04	Skin_Sun_Exposed_Lower_leg
chr3_52702426_G_A_b38	rs79705974	G	A	ENSG00000168268.10	NT5DC2	167372	0.489	0.126	1.10E-04	Muscle_Skeletal
chr3_52710420_T_G_b38	rs61220667	T	G	ENSG00000114841.17	DNAH1	394101	-0.568	0.125	1.31E-05	Brain_Cerebellar_Hemisphere
chr3_52710420_T_G_b38	rs61220667	T	G	ENSG00000168237.17	GLYCTK	423331	-0.313	0.084	2.23E-04	Thyroid
chr3_52710420_T_G_b38	rs61220667	T	G	ENSG00000163938.16	GNL3	29264	0.350	0.047	3.80E-13	Skin_Not_Sun_Exposed_Suprapubic
chr3_52710420_T_G_b38	rs61220667	T	G	ENSG00000168268.10	NT5DC2	175366	0.535	0.117	7.31E-06	Pancreas
chr3_52710420_T_G_b38	rs61220667	T	G	ENSG00000163938.16	GNL3	29264	0.328	0.061	1.36E-07	Esophagus_Mucosa
chr3_52710420_T_G_b38	rs61220667	T	G	ENSG00000010327.10	STAB1	215082	0.232	0.055	3.10E-05	Artery_Tibial
chr3_52710420_T_G_b38	rs61220667	T	G	ENSG00000168268.10	NT5DC2	175366	0.560	0.120	5.35E-06	Colon_Sigmoid
chr3_52710420_T_G_b38	rs61220667	T	G	ENSG00000168268.10	NT5DC2	175366	0.419	0.084	1.12E-06	Stomach
chr3_52710420_T_G_b38	rs61220667	T	G	ENSG00000168268.10	NT5DC2	175366	0.366	0.082	9.98E-06	Muscle_Skeletal
chr3_52710420_T_G_b38	rs61220667	T	G	ENSG00000168268.10	NT5DC2	175366	0.341	0.060	2.10E-08	Whole_Blood
chr3_52710420_T_G_b38	rs61220667	T	G	ENSG00000163938.16	GNL3	29264	0.270	0.049	5.95E-08	Skin_Sun_Exposed_Lower_leg
chr3_52716309_T_C_b38	rs116493126	T	C	ENSG00000163938.16	GNL3	35153	0.394	0.095	4.27E-05	Esophagus_Mucosa
chr3_52716309_T_C_b38	rs116493126	T	C	ENSG00000114841.17	DNAH1	399990	-0.725	0.167	2.76E-05	Brain_Cerebellar_Hemisphere
chr3_52716309_T_C_b38	rs116493126	T	C	ENSG00000163938.16	GNL3	35153	0.284	0.073	1.20E-04	Skin_Sun_Exposed_Lower_leg
chr3_52716309_T_C_b38	rs116493126	T	C	ENSG00000163938.16	GNL3	35153	0.414	0.074	3.34E-08	Skin_Not_Sun_Exposed_Suprapubic
chr3_52716309_T_C_b38	rs116493126	T	C	ENSG00000168268.10	NT5DC2	181255	0.489	0.126	1.10E-04	Muscle_Skeletal
chr3_52716309_T_C_b38	rs116493126	T	C	ENSG00000163930.9	BAP1	305959	-0.255	0.072	4.62E-04	Artery_Tibial
chr3_52716309_T_C_b38	rs116493126	T	C	ENSG00000247596.8	TWF2	477052	-0.384	0.099	1.33E-04	Cells_Cultured_fibroblast
chr3_52723637_G_A_b38	rs72960237	G	A	ENSG00000114841.17	DNAH1	407318	-0.463	0.115	9.25E-05	Brain_Cerebellar_Hemisphere
chr3_52723637_G_A_b38	rs72960237	G	A	ENSG00000163938.16	GNL3	42481	0.329	0.049	7.59E-11	Skin_Not_Sun_Exposed_Suprapubic
chr3_52723637_G_A_b38	rs72960237	G	A	ENSG00000168268.10	NT5DC2	188583	0.238	0.060	7.60E-05	Whole_Blood
chr3_52723637_G_A_b38	rs72960237	G	A	ENSG00000163938.16	GNL3	42481	0.273	0.049	4.48E-08	Skin_Sun_Exposed_Lower_leg
chr3_52723637_G_A_b38	rs72960237	G	A	ENSG00000247596.8	TWF2	484380	-0.224	0.052	2.29E-05	Cells_Cultured_fibroblast
chr3_52723637_G_A_b38	rs72960237	G	A	ENSG00000163938.16	GNL3	42481	0.279	0.062	8.12E-06	Esophagus_Mucosa
chr3_52723637_G_A_b38	rs72960237	G	A	ENSG00000242142.1	SERBP1P3	-341454	-0.539	0.147	2.78E-04	Nerve_Tibial

chr3_52727078_G_A_b38	rs113528713	G	A	ENSG00000168268.10	NT5DC2	192024	0.238	0.060	7.60E-05	Whole_Blood
chr3_52727078_G_A_b38	rs113528713	G	A	ENSG00000163938.16	GNL3	45922	0.329	0.049	7.59E-11	Skin_Not_Sun_Exposed_Suprapubic
chr3_52727078_G_A_b38	rs113528713	G	A	ENSG00000114841.17	DNAH1	410759	-0.463	0.115	9.25E-05	Brain_Cerebellar_Hemisphere
chr3_52727078_G_A_b38	rs113528713	G	A	ENSG00000163938.16	GNL3	45922	0.268	0.049	7.04E-08	Skin_Sun_Exposed_Lower_leg
chr3_52727078_G_A_b38	rs113528713	G	A	ENSG00000163938.16	GNL3	45922	0.279	0.062	8.12E-06	Esophagus_Mucosa
chr3_52727078_G_A_b38	rs113528713	G	A	ENSG00000247596.8	TWF2	487821	-0.224	0.052	2.29E-05	Cells_Cultured_fibroblast
chr3_52730334_T_G_b38	rs6769720	T	G	ENSG00000114841.17	DNAH1	414015	-0.568	0.125	1.31E-05	Brain_Cerebellar_Hemisphere
chr3_52730334_T_G_b38	rs6769720	T	G	ENSG00000168268.10	NT5DC2	195280	0.390	0.088	1.33E-05	Stomach
chr3_52730334_T_G_b38	rs6769720	T	G	ENSG00000114841.17	DNAH1	414015	-0.212	0.061	5.10E-04	Skin_Sun_Exposed_Lower_leg
chr3_52730334_T_G_b38	rs6769720	T	G	ENSG00000163938.16	GNL3	49178	0.250	0.052	2.03E-06	Skin_Sun_Exposed_Lower_leg
chr3_52730334_T_G_b38	rs6769720	T	G	ENSG00000168268.10	NT5DC2	195280	0.536	0.126	2.91E-05	Colon_Sigmoid
chr3_52730334_T_G_b38	rs6769720	T	G	ENSG00000168268.10	NT5DC2	195280	0.347	0.064	9.24E-08	Whole_Blood
chr3_52730334_T_G_b38	rs6769720	T	G	ENSG00000163938.16	GNL3	49178	0.328	0.050	1.39E-10	Skin_Not_Sun_Exposed_Suprapubic
chr3_52730334_T_G_b38	rs6769720	T	G	ENSG00000168268.10	NT5DC2	195280	0.548	0.129	3.27E-05	Pancreas
chr3_52730334_T_G_b38	rs6769720	T	G	ENSG00000163938.16	GNL3	49178	0.306	0.065	3.54E-06	Esophagus_Mucosa
chr3_52731933_G_A_b38	rs114687381	G	A	ENSG00000247596.8	TWF2	492676	-0.384	0.099	1.33E-04	Cells_Cultured_fibroblast
chr3_52731933_G_A_b38	rs114687381	G	A	ENSG00000163938.16	GNL3	50777	0.433	0.081	1.29E-07	Skin_Not_Sun_Exposed_Suprapubic
chr3_52731933_G_A_b38	rs114687381	G	A	ENSG00000114841.17	DNAH1	415614	-0.725	0.167	2.76E-05	Brain_Cerebellar_Hemisphere
chr3_52731933_G_A_b38	rs114687381	G	A	ENSG00000163938.16	GNL3	50777	0.446	0.105	2.63E-05	Esophagus_Mucosa
chr3_52731933_G_A_b38	rs114687381	G	A	ENSG00000168268.10	NT5DC2	196879	0.525	0.134	9.83E-05	Muscle_Skeletal
chr3_52732681_G_A_b38	rs764636	G	A	ENSG00000163938.16	GNL3	51525	0.316	0.050	4.96E-10	Skin_Not_Sun_Exposed_Suprapubic
chr3_52732681_G_A_b38	rs764636	G	A	ENSG00000247596.8	TWF2	493424	-0.224	0.052	2.29E-05	Cells_Cultured_fibroblast
chr3_52732681_G_A_b38	rs764636	G	A	ENSG00000163938.16	GNL3	51525	0.265	0.062	2.35E-05	Esophagus_Mucosa
chr3_52732681_G_A_b38	rs764636	G	A	ENSG00000163938.16	GNL3	51525	0.268	0.049	7.11E-08	Skin_Sun_Exposed_Lower_leg
chr3_52732681_G_A_b38	rs764636	G	A	ENSG00000168268.10	NT5DC2	197627	0.225	0.060	1.81E-04	Whole_Blood
chr3_52732681_G_A_b38	rs764636	G	A	ENSG00000114841.17	DNAH1	416362	-0.463	0.115	9.25E-05	Brain_Cerebellar_Hemisphere
chr3_52740997_G_A_b38	rs112824891	G	A	ENSG00000163938.16	GNL3	59841	0.279	0.062	8.12E-06	Esophagus_Mucosa
chr3_52740997_G_A_b38	rs112824891	G	A	ENSG00000114841.17	DNAH1	424678	-0.463	0.115	9.25E-05	Brain_Cerebellar_Hemisphere
chr3_52740997_G_A_b38	rs112824891	G	A	ENSG00000163938.16	GNL3	59841	0.329	0.049	7.59E-11	Skin_Not_Sun_Exposed_Suprapubic
chr3_52740997_G_A_b38	rs112824891	G	A	ENSG00000242142.1	SERBP1P3	-324094	-0.539	0.147	2.78E-04	Nerve_Tibial
chr3_52740997_G_A_b38	rs112824891	G	A	ENSG00000163938.16	GNL3	59841	0.273	0.049	4.48E-08	Skin_Sun_Exposed_Lower_leg
chr3_52740997_G_A_b38	rs112824891	G	A	ENSG00000247596.8	TWF2	501740	-0.224	0.052	2.29E-05	Cells_Cultured_fibroblast
chr3_52740997_G_A_b38	rs112824891	G	A	ENSG00000168268.10	NT5DC2	205943	0.238	0.060	7.60E-05	Whole_Blood
chr3_52741024_G_A_b38	rs151032151	G	A	ENSG00000163938.16	GNL3	59868	0.289	0.067	1.80E-05	Esophagus_Mucosa
chr3_52741024_G_A_b38	rs151032151	G	A	ENSG00000163938.16	GNL3	59868	0.263	0.053	9.79E-07	Skin_Sun_Exposed_Lower_leg
chr3_52741024_G_A_b38	rs151032151	G	A	ENSG00000163930.9	BAP1	330674	-0.220	0.052	2.97E-05	Artery_Tibial
chr3_52741024_G_A_b38	rs151032151	G	A	ENSG00000114841.17	DNAH1	424705	-0.575	0.127	1.34E-05	Brain_Cerebellar_Hemisphere
chr3_52741024_G_A_b38	rs151032151	G	A	ENSG00000114841.17	DNAH1	424705	-0.217	0.062	4.99E-04	Skin_Sun_Exposed_Lower_leg
chr3_52741024_G_A_b38	rs151032151	G	A	ENSG00000168268.10	NT5DC2	205970	0.538	0.135	9.18E-05	Pancreas
chr3_52741024_G_A_b38	rs151032151	G	A	ENSG00000168268.10	NT5DC2	205970	0.308	0.066	3.49E-06	Whole_Blood
chr3_52741024_G_A_b38	rs151032151	G	A	ENSG00000239732.3	TLR9	514861	0.261	0.074	4.13E-04	Thyroid
chr3_52741024_G_A_b38	rs151032151	G	A	ENSG00000163938.16	GNL3	59868	0.306	0.052	6.95E-09	Skin_Not_Sun_Exposed_Suprapubic
chr3_52742762_T_C_b38	rs78014137	T	C	ENSG00000168268.10	NT5DC2	207708	0.489	0.126	1.10E-04	Muscle_Skeletal
chr3_52742762_T_C_b38	rs78014137	T	C	ENSG00000114841.17	DNAH1	426443	-0.725	0.167	2.76E-05	Brain_Cerebellar_Hemisphere
chr3_52742762_T_C_b38	rs78014137	T	C	ENSG00000247596.8	TWF2	503505	-0.384	0.099	1.33E-04	Cells_Cultured_fibroblast
chr3_52742762_T_C_b38	rs78014137	T	C	ENSG00000163938.16	GNL3	61606	0.394	0.095	4.27E-05	Esophagus_Mucosa
chr3_52742762_T_C_b38	rs78014137	T	C	ENSG00000163938.16	GNL3	61606	0.284	0.073	1.20E-04	Skin_Sun_Exposed_Lower_leg
chr3_52742762_T_C_b38	rs78014137	T	C	ENSG00000163938.16	GNL3	61606	0.414	0.074	3.34E-08	Skin_Not_Sun_Exposed_Suprapubic
chr3_52742762_T_C_b38	rs78014137	T	C	ENSG00000163930.9	BAP1	332412	-0.255	0.072	4.62E-04	Artery_Tibial
chr3_52745959_T_C_b38	rs181612199	T	C	ENSG00000163930.9	BAP1	335609	-0.255	0.072	4.62E-04	Artery_Tibial
chr3_52745959_T_C_b38	rs181612199	T	C	ENSG00000163938.16	GNL3	64803	0.284	0.073	1.20E-04	Skin_Sun_Exposed_Lower_leg
chr3_52745959_T_C_b38	rs181612199	T	C	ENSG00000168268.10	NT5DC2	210905	0.489	0.126	1.10E-04	Muscle_Skeletal
chr3_52745959_T_C_b38	rs181612199	T	C	ENSG00000247596.8	TWF2	506702	-0.384	0.099	1.33E-04	Cells_Cultured_fibroblast

chr3_52745959_T_C_b38	rs181612199	T	C	ENSG00000163938.16	GNL3	64803	0.414	0.074	3.34E-08	Skin_Not_Sun_Exposed_Suprapubic
chr3_52745959_T_C_b38	rs181612199	T	C	ENSG00000114841.17	DNAH1	429640	-0.725	0.167	2.76E-05	Brain_Cerebellar_Hemisphere
chr3_52745959_T_C_b38	rs181612199	T	C	ENSG00000163938.16	GNL3	64803	0.394	0.095	4.27E-05	Esophagus_Mucosa
chr3_52749370_G_A_b38	rs190297652	G	A	ENSG00000163930.9	BAP1	339020	-0.255	0.072	4.62E-04	Artery_Tibial
chr3_52749370_G_A_b38	rs190297652	G	A	ENSG00000163938.16	GNL3	68214	0.284	0.073	1.20E-04	Skin_Sun_Exposed_Lower_leg
chr3_52749370_G_A_b38	rs190297652	G	A	ENSG00000163938.16	GNL3	68214	0.394	0.095	4.27E-05	Esophagus_Mucosa
chr3_52749370_G_A_b38	rs190297652	G	A	ENSG00000163938.16	GNL3	68214	0.414	0.074	3.34E-08	Skin_Not_Sun_Exposed_Suprapubic
chr3_52749370_G_A_b38	rs190297652	G	A	ENSG00000247596.8	TWF2	510113	-0.384	0.099	1.33E-04	Cells_Cultured_fibroblast
chr3_52749370_G_A_b38	rs190297652	G	A	ENSG00000168268.10	NT5DC2	214316	0.489	0.126	1.10E-04	Muscle_Skeletal
chr3_52749370_G_A_b38	rs190297652	G	A	ENSG00000114841.17	DNAH1	433051	-0.725	0.167	2.76E-05	Brain_Cerebellar_Hemisphere
chr3_52751167_G_A_b38	rs6445536	G	A	ENSG00000168268.10	NT5DC2	216113	0.347	0.064	9.24E-08	Whole_Blood
chr3_52751167_G_A_b38	rs6445536	G	A	ENSG00000163938.16	GNL3	70011	0.306	0.065	3.54E-06	Esophagus_Mucosa
chr3_52751167_G_A_b38	rs6445536	G	A	ENSG00000163938.16	GNL3	70011	0.250	0.052	2.03E-06	Skin_Sun_Exposed_Lower_leg
chr3_52751167_G_A_b38	rs6445536	G	A	ENSG00000163938.16	GNL3	70011	0.328	0.050	1.39E-10	Skin_Not_Sun_Exposed_Suprapubic
chr3_52751167_G_A_b38	rs6445536	G	A	ENSG00000168268.10	NT5DC2	216113	0.548	0.129	3.27E-05	Pancreas
chr3_52751167_G_A_b38	rs6445536	G	A	ENSG00000168268.10	NT5DC2	216113	0.390	0.088	1.33E-05	Stomach
chr3_52751167_G_A_b38	rs6445536	G	A	ENSG00000168268.10	NT5DC2	216113	0.536	0.126	2.91E-05	Colon_Sigmoid
chr3_52751167_G_A_b38	rs6445536	G	A	ENSG00000114841.17	DNAH1	434848	-0.568	0.125	1.31E-05	Brain_Cerebellar_Hemisphere
chr3_52751167_G_A_b38	rs6445536	G	A	ENSG00000114841.17	DNAH1	434848	-0.212	0.061	5.10E-04	Skin_Sun_Exposed_Lower_leg
chr3_52753086_G_A_b38	rs192710451	G	A	ENSG00000168268.10	NT5DC2	218032	0.238	0.060	7.60E-05	Whole_Blood
chr3_52753086_G_A_b38	rs192710451	G	A	ENSG00000163938.16	GNL3	71930	0.279	0.062	8.12E-06	Esophagus_Mucosa
chr3_52753086_G_A_b38	rs192710451	G	A	ENSG00000247596.8	TWF2	513829	-0.224	0.052	2.29E-05	Cells_Cultured_fibroblast
chr3_52753086_G_A_b38	rs192710451	G	A	ENSG00000163938.16	GNL3	71930	0.329	0.049	7.59E-11	Skin_Not_Sun_Exposed_Suprapubic
chr3_52753086_G_A_b38	rs192710451	G	A	ENSG00000242142.1	SERBP1P3	-312005	-0.539	0.147	2.78E-04	Nerve_Tibial
chr3_52753086_G_A_b38	rs192710451	G	A	ENSG00000114841.17	DNAH1	436767	-0.463	0.115	9.25E-05	Brain_Cerebellar_Hemisphere
chr3_52753086_G_A_b38	rs192710451	G	A	ENSG00000163938.16	GNL3	71930	0.273	0.049	4.48E-08	Skin_Sun_Exposed_Lower_leg
chr3_52754103_G_A_b38	rs7619643	G	A	ENSG00000163938.16	GNL3	72947	0.306	0.065	3.54E-06	Esophagus_Mucosa
chr3_52754103_G_A_b38	rs7619643	G	A	ENSG00000168268.10	NT5DC2	219049	0.548	0.129	3.27E-05	Pancreas
chr3_52754103_G_A_b38	rs7619643	G	A	ENSG00000114841.17	DNAH1	437784	-0.568	0.125	1.31E-05	Brain_Cerebellar_Hemisphere
chr3_52754103_G_A_b38	rs7619643	G	A	ENSG00000168268.10	NT5DC2	219049	0.347	0.064	9.24E-08	Whole_Blood
chr3_52754103_G_A_b38	rs7619643	G	A	ENSG00000163938.16	GNL3	72947	0.328	0.050	1.39E-10	Skin_Not_Sun_Exposed_Suprapubic
chr3_52754103_G_A_b38	rs7619643	G	A	ENSG00000168268.10	NT5DC2	219049	0.536	0.126	2.91E-05	Colon_Sigmoid
chr3_52754103_G_A_b38	rs7619643	G	A	ENSG00000114841.17	DNAH1	437784	-0.212	0.061	5.10E-04	Skin_Sun_Exposed_Lower_leg
chr3_52754103_G_A_b38	rs7619643	G	A	ENSG00000168268.10	NT5DC2	219049	0.390	0.088	1.33E-05	Stomach
chr3_52754103_G_A_b38	rs7619643	G	A	ENSG00000163938.16	GNL3	72947	0.250	0.052	2.03E-06	Skin_Sun_Exposed_Lower_leg
chr3_52762646_G_A_b38	rs115801090	G	A	ENSG00000168268.10	NT5DC2	227592	0.489	0.126	1.10E-04	Muscle_Skeletal
chr3_52762646_G_A_b38	rs115801090	G	A	ENSG00000163938.16	GNL3	81490	0.394	0.095	4.27E-05	Esophagus_Mucosa
chr3_52762646_G_A_b38	rs115801090	G	A	ENSG00000163930.9	BAP1	352296	-0.255	0.072	4.62E-04	Artery_Tibial
chr3_52762646_G_A_b38	rs115801090	G	A	ENSG00000114841.17	DNAH1	446327	-0.725	0.167	2.76E-05	Brain_Cerebellar_Hemisphere
chr3_52762646_G_A_b38	rs115801090	G	A	ENSG00000163938.16	GNL3	81490	0.284	0.073	1.20E-04	Skin_Sun_Exposed_Lower_leg
chr3_52762646_G_A_b38	rs115801090	G	A	ENSG00000247596.8	TWF2	523389	-0.384	0.099	1.33E-04	Cells_Cultured_fibroblast
chr3_52762646_G_A_b38	rs115801090	G	A	ENSG00000163938.16	GNL3	81490	0.414	0.074	3.34E-08	Skin_Not_Sun_Exposed_Suprapubic
chr3_52767623_C_A_b38	rs58564212	C	A	ENSG00000247596.8	TWF2	528366	-0.224	0.052	2.29E-05	Cells_Cultured_fibroblast
chr3_52767623_C_A_b38	rs58564212	C	A	ENSG00000114841.17	DNAH1	451304	-0.463	0.115	9.25E-05	Brain_Cerebellar_Hemisphere
chr3_52767623_C_A_b38	rs58564212	C	A	ENSG00000242142.1	SERBP1P3	-297468	-0.539	0.147	2.78E-04	Nerve_Tibial
chr3_52767623_C_A_b38	rs58564212	C	A	ENSG00000163938.16	GNL3	86467	0.329	0.049	7.59E-11	Skin_Not_Sun_Exposed_Suprapubic
chr3_52767623_C_A_b38	rs58564212	C	A	ENSG00000163938.16	GNL3	86467	0.279	0.062	8.12E-06	Esophagus_Mucosa
chr3_52767623_C_A_b38	rs58564212	C	A	ENSG00000163938.16	GNL3	86467	0.273	0.049	4.48E-08	Skin_Sun_Exposed_Lower_leg
chr3_52767623_C_A_b38	rs58564212	C	A	ENSG00000168268.10	NT5DC2	232569	0.238	0.060	7.60E-05	Whole_Blood
chr3_52773429_T_C_b38	rs185761183	T	C	ENSG00000163938.16	GNL3	92273	0.414	0.074	3.34E-08	Skin_Not_Sun_Exposed_Suprapubic
chr3_52773429_T_C_b38	rs185761183	T	C	ENSG00000247596.8	TWF2	534172	-0.384	0.099	1.33E-04	Cells_Cultured_fibroblast
chr3_52773429_T_C_b38	rs185761183	T	C	ENSG00000163938.16	GNL3	92273	0.394	0.095	4.27E-05	Esophagus_Mucosa
chr3_52773429_T_C_b38	rs185761183	T	C	ENSG00000168268.10	NT5DC2	238375	0.489	0.126	1.10E-04	Muscle_Skeletal

chr3_52773429_T_C_b38	rs185761183	T	C	ENSG00000163930.9	BAP1	363079	-0.255	0.072	4.62E-04	Artery_Tibial
chr3_52773429_T_C_b38	rs185761183	T	C	ENSG00000163938.16	GNL3	92273	0.284	0.073	1.20E-04	Skin_Sun_Exposed_Lower_leg
chr3_52773429_T_C_b38	rs185761183	T	C	ENSG00000114841.17	DNAH1	457110	-0.725	0.167	2.76E-05	Brain_Cerebellar_Hemisphere
chr3_52773800_T_G_b38	rs72960285	T	G	ENSG00000168268.10	NT5DC2	238746	0.360	0.087	4.46E-05	Whole_Blood
chr3_52773800_T_G_b38	rs72960285	T	G	ENSG00000163938.16	GNL3	92644	0.403	0.063	5.30E-10	Skin_Not_Sun_Exposed_Suprapubic
chr3_52773800_T_G_b38	rs72960285	T	G	ENSG00000114841.17	DNAH1	457481	-0.656	0.158	5.78E-05	Brain_Cerebellar_Hemisphere
chr3_52773800_T_G_b38	rs72960285	T	G	ENSG00000163938.16	GNL3	92644	0.316	0.081	1.10E-04	Esophagus_Mucosa
chr3_52773800_T_G_b38	rs72960285	T	G	ENSG00000163938.16	GNL3	92644	0.244	0.064	1.67E-04	Skin_Sun_Exposed_Lower_leg
chr3_52773800_T_G_b38	rs72960285	T	G	ENSG00000168268.10	NT5DC2	238746	0.403	0.109	2.41E-04	Muscle_Skeletal
chr3_52846426_G_A_b38	rs76252265	G	A	ENSG00000247596.8	TWF2	607169	-0.347	0.095	2.92E-04	Cells_Cultured_fibroblast
chr3_52846426_G_A_b38	rs76252265	G	A	ENSG00000163938.16	GNL3	165270	0.387	0.106	2.77E-04	Esophagus_Mucosa
chr3_52846426_G_A_b38	rs76252265	G	A	ENSG00000163938.16	GNL3	165270	0.387	0.080	1.97E-06	Skin_Not_Sun_Exposed_Suprapubic
chr3_52846426_G_A_b38	rs76252265	G	A	ENSG00000168268.10	NT5DC2	311372	0.482	0.135	3.76E-04	Muscle_Skeletal
chr3_52853366_G_C_b38	rs77782057	G	C	ENSG00000163938.16	GNL3	172210	0.388	0.102	1.63E-04	Esophagus_Mucosa
chr3_52853366_G_C_b38	rs77782057	G	C	ENSG00000247596.8	TWF2	614109	-0.347	0.095	2.92E-04	Cells_Cultured_fibroblast
chr3_52853366_G_C_b38	rs77782057	G	C	ENSG00000163938.16	GNL3	172210	0.397	0.078	6.10E-07	Skin_Not_Sun_Exposed_Suprapubic
chr3_52853366_G_C_b38	rs77782057	G	C	ENSG00000168268.10	NT5DC2	318312	0.475	0.131	3.13E-04	Muscle_Skeletal
chr5_107851606_C_A_b38	rs6867085	C	A	ENSG00000145743.15	FBXL17	-530492	-0.093	0.021	1.22E-05	Esophagus_Muscularis
chr5_107851606_C_A_b38	rs6867085	C	A	ENSG00000145743.15	FBXL17	-530492	-0.088	0.015	1.11E-08	Muscle_Skeletal
chr5_81295429_T_A_b38	rs190923216	T	A	ENSG00000248794.1	NA	52830	1.135	0.261	1.95E-05	Stomach
chr5_81295429_T_A_b38	rs190923216	T	A	ENSG00000247572.7	CKMT2-AS1	-6131	0.571	0.146	1.05E-04	Cells_Cultured_fibroblast
chr5_81295429_T_A_b38	rs190923216	T	A	ENSG00000248794.1	NA	52830	1.052	0.252	4.00E-05	Testis
chr5_81295429_T_A_b38	rs190923216	T	A	ENSG00000248794.1	NA	52830	1.008	0.213	3.19E-06	Adipose_Visceral_Omentum
chr5_81295429_T_A_b38	rs190923216	T	A	ENSG00000248794.1	NA	52830	1.121	0.219	4.59E-07	Esophagus_Mucosa
chr5_81295429_T_A_b38	rs190923216	T	A	ENSG00000247572.7	CKMT2-AS1	-6131	1.463	0.358	6.62E-05	Brain_Cerebellum
chr5_81295429_T_A_b38	rs190923216	T	A	ENSG00000247572.7	CKMT2-AS1	-6131	0.648	0.156	3.85E-05	Skin_Not_Sun_Exposed_Suprapubic
chr5_81295429_T_A_b38	rs190923216	T	A	ENSG00000248794.1	NA	52830	1.073	0.228	3.29E-06	Thyroid
chr5_81295429_T_A_b38	rs190923216	T	A	ENSG00000247572.7	CKMT2-AS1	-6131	1.671	0.360	7.14E-06	Brain_Nucleus_accumbens_basal_ganglia
chr5_81295429_T_A_b38	rs190923216	T	A	ENSG00000248794.1	NA	52830	0.954	0.210	7.36E-06	Skin_Not_Sun_Exposed_Suprapubic
chr5_81295429_T_A_b38	rs190923216	T	A	ENSG00000247572.7	CKMT2-AS1	-6131	1.496	0.299	1.21E-06	Pituitary
chr5_81295429_T_A_b38	rs190923216	T	A	ENSG00000247572.7	CKMT2-AS1	-6131	0.793	0.152	2.95E-07	Adipose_Visceral_Omentum
chr5_81295429_T_A_b38	rs190923216	T	A	ENSG00000248794.1	NA	52830	0.943	0.223	2.69E-05	Adipose_Subcutaneous
chr5_81295429_T_A_b38	rs190923216	T	A	ENSG00000248794.1	NA	52830	1.062	0.239	1.16E-05	Esophagus_Muscularis
chr5_81295429_T_A_b38	rs190923216	T	A	ENSG00000248794.1	NA	52830	1.053	0.222	2.72E-06	Lung
chr5_81295429_T_A_b38	rs190923216	T	A	ENSG00000131730.15	CKMT2	62144	-0.822	0.193	3.51E-05	Brain_Nucleus_accumbens_basal_ganglia
chr5_81295429_T_A_b38	rs190923216	T	A	ENSG00000248794.1	NA	52830	0.851	0.206	4.13E-05	Artery_Tibial
chr5_81295429_T_A_b38	rs190923216	T	A	ENSG00000247572.7	CKMT2-AS1	-6131	1.015	0.192	1.97E-07	Nerve_Tibial
chr5_81295429_T_A_b38	rs190923216	T	A	ENSG00000248794.1	NA	52830	1.112	0.276	7.25E-05	Colon_Transverse
chr5_81295429_T_A_b38	rs190923216	T	A	ENSG00000247572.7	CKMT2-AS1	-6131	0.798	0.169	3.01E-06	Lung
chr5_81295429_T_A_b38	rs190923216	T	A	ENSG00000248794.1	NA	52830	1.515	0.319	3.84E-06	Pituitary
chr5_81295429_T_A_b38	rs190923216	T	A	ENSG00000247572.7	CKMT2-AS1	-6131	0.603	0.151	7.42E-05	Esophagus_Mucosa
chr5_81295429_T_A_b38	rs190923216	T	A	ENSG00000247572.7	CKMT2-AS1	-6131	0.561	0.130	1.96E-05	Muscle_Skeletal
chr5_88143692_T_A_b38	rs143183500	T	A	ENSG00000271904.1	NA	-355005	-0.827	0.208	1.05E-04	Brain_Nucleus_accumbens_basal_ganglia
chr5_88143692_T_A_b38	rs143183500	T	A	ENSG00000250156.3	LINC02060	-295398	0.551	0.144	1.64E-04	Testis
chr5_88145768_T_G_b38	rs114221565	T	G	ENSG00000271904.1	NA	-352929	-0.827	0.208	1.05E-04	Brain_Nucleus_accumbens_basal_ganglia
chr5_88145768_T_G_b38	rs114221565	T	G	ENSG00000250156.3	LINC02060	-293322	0.551	0.144	1.64E-04	Testis
chr7_130527710_T_C_b38		T	C	ENSG00000158623.14	COPG2	-140937	1.136	0.090	2.24E-31	Cells_Cultured_fibroblast
chr7_130527710_T_C_b38		T	C	ENSG00000158623.14	COPG2	-140937	0.715	0.050	2.26E-39	Thyroid
chr7_130527710_T_C_b38		T	C	ENSG00000158623.14	COPG2	-140937	0.653	0.099	2.12E-10	Esophagus_Gastroesophageal_junction
chr7_130527710_T_C_b38		T	C	ENSG00000158623.14	COPG2	-140937	0.573	0.066	1.39E-16	Esophagus_Muscularis
chr7_130527710_T_C_b38		T	C	ENSG00000158623.14	COPG2	-140937	0.704	0.109	8.21E-10	Prostate
chr7_130527710_T_C_b38		T	C	ENSG00000158623.14	COPG2	-140937	0.476	0.095	1.31E-06	Liver
chr7_130527710_T_C_b38		T	C	ENSG00000158623.14	COPG2	-140937	0.747	0.071	4.26E-23	Lung

chr7_130527710_T_C_b38	T	C	ENSG00000158623.14	COPG2	-140937	0.869	0.075	4.63E-27	Skin_Not_Sun_Exposed_Suprapubic
chr7_130527710_T_C_b38	T	C	ENSG00000158623.14	COPG2	-140937	0.891	0.070	7.81E-32	Esophagus_Mucosa
chr7_130527710_T_C_b38	T	C	ENSG00000158623.14	COPG2	-140937	0.897	0.111	9.70E-14	Spleen
chr7_130527710_T_C_b38	T	C	ENSG00000158623.14	COPG2	-140937	0.593	0.092	6.40E-10	Colon_Sigmoid
chr7_130527710_T_C_b38	T	C	ENSG00000158623.14	COPG2	-140937	0.820	0.098	1.17E-14	Adrenal_Gland
chr7_130527710_T_C_b38	T	C	ENSG00000158623.14	COPG2	-140937	0.771	0.077	1.47E-21	Adipose_Subcutaneous
chr7_130527710_T_C_b38	T	C	ENSG00000158623.14	COPG2	-140937	0.516	0.052	2.46E-21	Artery_Tibial
chr7_130527710_T_C_b38	T	C	ENSG00000158623.14	COPG2	-140937	0.621	0.082	1.12E-12	Pituitary
chr7_130527710_T_C_b38	T	C	ENSG00000158623.14	COPG2	-140937	0.479	0.058	5.91E-15	Testis
chr7_130527710_T_C_b38	T	C	ENSG00000158623.14	COPG2	-140937	0.754	0.087	2.89E-16	Colon_Transverse
chr7_130527710_T_C_b38	T	C	ENSG00000158623.14	COPG2	-140937	0.348	0.063	7.08E-08	Heart_Atrial_Appendage
chr7_130527710_T_C_b38	T	C	ENSG00000272701.2	MESTIT1	36677	0.310	0.077	6.62E-05	Adipose_Subcutaneous
chr7_130527710_T_C_b38	T	C	ENSG00000158623.14	COPG2	-140937	0.490	0.083	8.46E-09	Artery_Aorta
chr7_130527710_T_C_b38	T	C	ENSG00000158623.14	COPG2	-140937	0.675	0.096	1.03E-11	Breast
chr7_130527710_T_C_b38	T	C	ENSG00000158623.14	COPG2	-140937	0.849	0.104	1.85E-14	Pancreas
chr7_130527710_T_C_b38	T	C	ENSG00000158623.14	COPG2	-140937	0.671	0.104	4.87E-10	Stomach
chr7_130527710_T_C_b38	T	C	ENSG00000158623.14	COPG2	-140937	0.589	0.060	3.06E-21	Whole_Blood
chr7_130527710_T_C_b38	T	C	ENSG00000106484.14	MEST	36381	0.381	0.095	7.65E-05	Esophagus_Mucosa
chr7_130527710_T_C_b38	T	C	ENSG00000158623.14	COPG2	-140937	0.655	0.076	1.59E-16	Adipose_Visceral_Omentum
chr7_130527710_T_C_b38	T	C	ENSG00000158623.14	COPG2	-140937	0.462	0.062	3.44E-13	Nerve_Tibial
chr7_130527710_T_C_b38	T	C	ENSG00000158623.14	COPG2	-140937	0.920	0.067	5.56E-37	Skin_Sun_Exposed_Lower_leg
chr7_130527710_T_C_b38	T	C	ENSG00000158623.14	COPG2	-140937	0.231	0.048	2.29E-06	Heart_Left_Ventricle
chr7_130527710_T_C_b38	T	C	ENSG00000158623.14	COPG2	-140937	0.781	0.122	2.15E-09	Small_Intestine_Terminal_Ileum
chr7_130527721_C_A_b38	C	A	ENSG00000158623.14	COPG2	-140926	0.573	0.066	1.39E-16	Esophagus_Muscularis
chr7_130527721_C_A_b38	C	A	ENSG00000158623.14	COPG2	-140926	1.136	0.090	2.24E-31	Cells_Cultured_fibroblast
chr7_130527721_C_A_b38	C	A	ENSG00000158623.14	COPG2	-140926	0.869	0.075	4.63E-27	Skin_Not_Sun_Exposed_Suprapubic
chr7_130527721_C_A_b38	C	A	ENSG00000158623.14	COPG2	-140926	0.593	0.092	6.40E-10	Colon_Sigmoid
chr7_130527721_C_A_b38	C	A	ENSG00000158623.14	COPG2	-140926	0.891	0.070	7.81E-32	Esophagus_Mucosa
chr7_130527721_C_A_b38	C	A	ENSG00000158623.14	COPG2	-140926	0.348	0.063	7.08E-08	Heart_Atrial_Appendage
chr7_130527721_C_A_b38	C	A	ENSG00000158623.14	COPG2	-140926	0.704	0.109	8.21E-10	Prostate
chr7_130527721_C_A_b38	C	A	ENSG00000158623.14	COPG2	-140926	0.516	0.052	2.46E-21	Artery_Tibial
chr7_130527721_C_A_b38	C	A	ENSG00000158623.14	COPG2	-140926	0.476	0.095	1.31E-06	Liver
chr7_130527721_C_A_b38	C	A	ENSG00000158623.14	COPG2	-140926	0.754	0.087	2.89E-16	Colon_Transverse
chr7_130527721_C_A_b38	C	A	ENSG00000158623.14	COPG2	-140926	0.897	0.111	9.70E-14	Spleen
chr7_130527721_C_A_b38	C	A	ENSG00000158623.14	COPG2	-140926	0.671	0.104	4.87E-10	Stomach
chr7_130527721_C_A_b38	C	A	ENSG00000158623.14	COPG2	-140926	0.655	0.076	1.59E-16	Adipose_Visceral_Omentum
chr7_130527721_C_A_b38	C	A	ENSG00000158623.14	COPG2	-140926	0.771	0.077	1.47E-21	Adipose_Subcutaneous
chr7_130527721_C_A_b38	C	A	ENSG00000158623.14	COPG2	-140926	0.820	0.098	1.17E-14	Adrenal_Gland
chr7_130527721_C_A_b38	C	A	ENSG00000158623.14	COPG2	-140926	0.849	0.104	1.85E-14	Pancreas
chr7_130527721_C_A_b38	C	A	ENSG00000106484.14	MEST	36392	0.381	0.095	7.65E-05	Esophagus_Mucosa
chr7_130527721_C_A_b38	C	A	ENSG00000158623.14	COPG2	-140926	0.589	0.060	3.06E-21	Whole_Blood
chr7_130527721_C_A_b38	C	A	ENSG00000158623.14	COPG2	-140926	0.621	0.082	1.12E-12	Pituitary
chr7_130527721_C_A_b38	C	A	ENSG00000158623.14	COPG2	-140926	0.675	0.096	1.03E-11	Breast
chr7_130527721_C_A_b38	C	A	ENSG00000158623.14	COPG2	-140926	0.747	0.071	4.26E-23	Lung
chr7_130527721_C_A_b38	C	A	ENSG00000158623.14	COPG2	-140926	0.231	0.048	2.29E-06	Heart_Left_Ventricle
chr7_130527721_C_A_b38	C	A	ENSG00000158623.14	COPG2	-140926	0.490	0.083	8.46E-09	Artery_Aorta
chr7_130527721_C_A_b38	C	A	ENSG00000158623.14	COPG2	-140926	0.653	0.099	2.12E-10	Esophagus_Gastroesophageal_junction
chr7_130527721_C_A_b38	C	A	ENSG00000158623.14	COPG2	-140926	0.479	0.058	5.91E-15	Testis
chr7_130527721_C_A_b38	C	A	ENSG00000272701.2	MESTIT1	36688	0.310	0.077	6.62E-05	Adipose_Subcutaneous
chr7_130527721_C_A_b38	C	A	ENSG00000158623.14	COPG2	-140926	0.715	0.050	2.26E-39	Thyroid
chr7_130527721_C_A_b38	C	A	ENSG00000158623.14	COPG2	-140926	0.920	0.067	5.56E-37	Skin_Sun_Exposed_Lower_leg
chr7_130527721_C_A_b38	C	A	ENSG00000158623.14	COPG2	-140926	0.781	0.122	2.15E-09	Small_Intestine_Terminal_Ileum
chr7_130527721_C_A_b38	C	A	ENSG00000158623.14	COPG2	-140926	0.462	0.062	3.44E-13	Nerve_Tibial

chr7_130574654_C_A_b38	rs36168950	C	A	ENSG00000158623.14	COPG2	-93993	0.603	0.094	1.21E-09	Adrenal_Gland
chr7_130574654_C_A_b38	rs36168950	C	A	ENSG00000158623.14	COPG2	-93993	0.419	0.058	1.90E-12	Esophagus_Muscularis
chr7_130574654_C_A_b38	rs36168950	C	A	ENSG00000158623.14	COPG2	-93993	0.493	0.092	1.94E-07	Stomach
chr7_130574654_C_A_b38	rs36168950	C	A	ENSG00000158623.14	COPG2	-93993	0.413	0.094	2.03E-05	Prostate
chr7_130574654_C_A_b38	rs36168950	C	A	ENSG00000158623.14	COPG2	-93993	0.427	0.079	1.63E-07	Colon_Sigmoid
chr7_130574654_C_A_b38	rs36168950	C	A	ENSG00000158623.14	COPG2	-93993	0.299	0.052	2.10E-08	Nerve_Tibial
chr7_130574654_C_A_b38	rs36168950	C	A	ENSG00000158623.14	COPG2	-93993	0.815	0.080	4.39E-22	Cells_Cultured_fibroblast
chr7_130574654_C_A_b38	rs36168950	C	A	ENSG00000158623.14	COPG2	-93993	0.394	0.046	1.15E-16	Artery_Tibial
chr7_130574654_C_A_b38	rs36168950	C	A	ENSG00000158623.14	COPG2	-93993	0.525	0.077	4.27E-11	Colon_Transverse
chr7_130574654_C_A_b38	rs36168950	C	A	ENSG00000272701.2	MESTIT1	83621	0.276	0.064	1.82E-05	Adipose_Subcutaneous
chr7_130574654_C_A_b38	rs36168950	C	A	ENSG00000158623.14	COPG2	-93993	0.618	0.066	2.77E-19	Esophagus_Mucosa
chr7_130574654_C_A_b38	rs36168950	C	A	ENSG00000158623.14	COPG2	-93993	0.638	0.105	5.94E-09	Spleen
chr7_130574654_C_A_b38	rs36168950	C	A	ENSG00000158623.14	COPG2	-93993	0.450	0.101	1.70E-05	Small_Intestine_Terminal_Ileum
chr7_130574654_C_A_b38	rs36168950	C	A	ENSG00000158623.14	COPG2	-93993	0.243	0.053	6.30E-06	Heart_Atrial_Appendage
chr7_130574654_C_A_b38	rs36168950	C	A	ENSG00000158623.14	COPG2	-93993	0.502	0.046	6.40E-25	Thyroid
chr7_130574654_C_A_b38	rs36168950	C	A	ENSG00000158623.14	COPG2	-93993	0.275	0.050	9.66E-08	Testis
chr7_130574654_C_A_b38	rs36168950	C	A	ENSG00000158623.14	COPG2	-93993	0.392	0.051	9.66E-14	Whole_Blood
chr7_130574654_C_A_b38	rs36168950	C	A	ENSG00000158623.14	COPG2	-93993	0.448	0.084	1.84E-07	Breast
chr7_130574654_C_A_b38	rs36168950	C	A	ENSG00000158623.14	COPG2	-93993	0.499	0.068	8.00E-13	Lung
chr7_130574654_C_A_b38	rs36168950	C	A	ENSG00000158623.14	COPG2	-93993	0.294	0.073	6.35E-05	Artery_Aorta
chr7_130574654_C_A_b38	rs36168950	C	A	ENSG00000158623.14	COPG2	-93993	0.520	0.066	2.18E-14	Adipose_Subcutaneous
chr7_130574654_C_A_b38	rs36168950	C	A	ENSG00000158623.14	COPG2	-93993	0.454	0.076	1.20E-08	Pituitary
chr7_130574654_C_A_b38	rs36168950	C	A	ENSG00000158623.14	COPG2	-93993	0.368	0.082	1.30E-05	Liver
chr7_130574654_C_A_b38	rs36168950	C	A	ENSG00000158623.14	COPG2	-93993	0.645	0.060	2.21E-24	Skin_Sun_Exposed_Lower_leg
chr7_130574654_C_A_b38	rs36168950	C	A	ENSG00000158623.14	COPG2	-93993	0.483	0.065	7.25E-13	Adipose_Visceral_Omentum
chr7_130574654_C_A_b38	rs36168950	C	A	ENSG00000158623.14	COPG2	-93993	0.559	0.067	8.38E-16	Skin_Not_Sun_Exposed_Suprapubic
chr7_130574654_C_A_b38	rs36168950	C	A	ENSG00000158623.14	COPG2	-93993	0.600	0.095	1.01E-09	Pancreas
chr7_130628875_T_C_b38	rs6467310	T	C	ENSG00000158623.14	COPG2	-39772	0.754	0.087	2.89E-16	Colon_Transverse
chr7_130628875_T_C_b38	rs6467310	T	C	ENSG00000158623.14	COPG2	-39772	0.492	0.056	2.91E-16	Testis
chr7_130628875_T_C_b38	rs6467310	T	C	ENSG00000158623.14	COPG2	-39772	0.674	0.095	7.55E-12	Breast
chr7_130628875_T_C_b38	rs6467310	T	C	ENSG00000158623.14	COPG2	-39772	0.593	0.060	1.00E-21	Whole_Blood
chr7_130628875_T_C_b38	rs6467310	T	C	ENSG00000158623.14	COPG2	-39772	0.458	0.061	3.64E-13	Nerve_Tibial
chr7_130628875_T_C_b38	rs6467310	T	C	ENSG00000272701.2	MESTIT1	137842	0.319	0.076	3.41E-05	Adipose_Subcutaneous
chr7_130628875_T_C_b38	rs6467310	T	C	ENSG00000158623.14	COPG2	-39772	0.842	0.103	1.51E-14	Pancreas
chr7_130628875_T_C_b38	rs6467310	T	C	ENSG00000158623.14	COPG2	-39772	0.747	0.071	4.26E-23	Lung
chr7_130628875_T_C_b38	rs6467310	T	C	ENSG00000158623.14	COPG2	-39772	0.925	0.066	4.92E-38	Skin_Sun_Exposed_Lower_leg
chr7_130628875_T_C_b38	rs6467310	T	C	ENSG00000158623.14	COPG2	-39772	0.653	0.099	2.12E-10	Esophagus_Gastroesophageal_junction
chr7_130628875_T_C_b38	rs6467310	T	C	ENSG00000158623.14	COPG2	-39772	0.905	0.068	8.89E-34	Esophagus_Mucosa
chr7_130628875_T_C_b38	rs6467310	T	C	ENSG00000158623.14	COPG2	-39772	0.897	0.111	9.70E-14	Spleen
chr7_130628875_T_C_b38	rs6467310	T	C	ENSG00000158623.14	COPG2	-39772	0.570	0.065	7.60E-17	Esophagus_Muscularis
chr7_130628875_T_C_b38	rs6467310	T	C	ENSG00000158623.14	COPG2	-39772	0.820	0.098	1.17E-14	Adrenal_Gland
chr7_130628875_T_C_b38	rs6467310	T	C	ENSG00000158623.14	COPG2	-39772	0.492	0.082	4.77E-09	Artery_Aorta
chr7_130628875_T_C_b38	rs6467310	T	C	ENSG00000158623.14	COPG2	-39772	0.718	0.049	2.86E-40	Thyroid
chr7_130628875_T_C_b38	rs6467310	T	C	ENSG00000158623.14	COPG2	-39772	0.617	0.081	8.31E-13	Pituitary
chr7_130628875_T_C_b38	rs6467310	T	C	ENSG00000106484.14	MEST	137546	0.293	0.076	1.34E-04	Thyroid
chr7_130628875_T_C_b38	rs6467310	T	C	ENSG00000158623.14	COPG2	-39772	0.662	0.075	3.52E-17	Adipose_Visceral_Omentum
chr7_130628875_T_C_b38	rs6467310	T	C	ENSG00000158623.14	COPG2	-39772	0.671	0.104	4.87E-10	Stomach
chr7_130628875_T_C_b38	rs6467310	T	C	ENSG00000158623.14	COPG2	-39772	0.763	0.077	1.73E-21	Adipose_Subcutaneous
chr7_130628875_T_C_b38	rs6467310	T	C	ENSG00000158623.14	COPG2	-39772	1.166	0.088	7.24E-34	Cells_Cultured_fibroblast
chr7_130628875_T_C_b38	rs6467310	T	C	ENSG00000158623.14	COPG2	-39772	0.869	0.075	4.63E-27	Skin_Not_Sun_Exposed_Suprapubic
chr7_130628875_T_C_b38	rs6467310	T	C	ENSG00000158623.14	COPG2	-39772	0.516	0.052	1.17E-21	Artery_Tibial
chr7_130628875_T_C_b38	rs6467310	T	C	ENSG00000158623.14	COPG2	-39772	0.781	0.122	2.15E-09	Small_Intestine_Terminal_Ileum
chr7_130628875_T_C_b38	rs6467310	T	C	ENSG00000158623.14	COPG2	-39772	0.589	0.090	3.78E-10	Colon_Sigmoid

chr7_130628875_T_C_b38	rs6467310	T	C	ENSG00000158623.14	COPG2	-39772	0.234	0.048	1.32E-06	Heart_Left_Ventricle
chr7_130628875_T_C_b38	rs6467310	T	C	ENSG00000106484.14	MEST	137546	0.431	0.094	5.85E-06	Esophagus_Mucosa
chr7_130628875_T_C_b38	rs6467310	T	C	ENSG00000158623.14	COPG2	-39772	0.476	0.095	1.31E-06	Liver
chr7_130628875_T_C_b38	rs6467310	T	C	ENSG00000158623.14	COPG2	-39772	0.336	0.062	1.24E-07	Heart_Atrial_Appendage
chr7_130628875_T_C_b38	rs6467310	T	C	ENSG00000158623.14	COPG2	-39772	0.704	0.106	3.11E-10	Prostate
chr7_130649969_T_G_b38	rs13237881	T	G	ENSG00000158623.14	COPG2	-18678	0.336	0.062	1.24E-07	Heart_Atrial_Appendage
chr7_130649969_T_G_b38	rs13237881	T	G	ENSG00000158623.14	COPG2	-18678	0.704	0.106	3.11E-10	Prostate
chr7_130649969_T_G_b38	rs13237881	T	G	ENSG00000158623.14	COPG2	-18678	0.905	0.068	8.89E-34	Esophagus_Mucosa
chr7_130649969_T_G_b38	rs13237881	T	G	ENSG00000158623.14	COPG2	-18678	0.476	0.095	1.31E-06	Liver
chr7_130649969_T_G_b38	rs13237881	T	G	ENSG00000158623.14	COPG2	-18678	0.662	0.075	3.52E-17	Adipose_Visceral_Omentum
chr7_130649969_T_G_b38	rs13237881	T	G	ENSG00000158623.14	COPG2	-18678	0.718	0.049	2.86E-40	Thyroid
chr7_130649969_T_G_b38	rs13237881	T	G	ENSG00000158623.14	COPG2	-18678	1.166	0.088	7.24E-34	Cells_Cultured_fibroblast
chr7_130649969_T_G_b38	rs13237881	T	G	ENSG00000158623.14	COPG2	-18678	0.516	0.052	1.17E-21	Artery_Tibial
chr7_130649969_T_G_b38	rs13237881	T	G	ENSG00000158623.14	COPG2	-18678	0.492	0.056	2.91E-16	Testis
chr7_130649969_T_G_b38	rs13237881	T	G	ENSG00000158623.14	COPG2	-18678	0.754	0.087	2.89E-16	Colon_Transverse
chr7_130649969_T_G_b38	rs13237881	T	G	ENSG00000272701.2	MESTIT1	158936	0.319	0.076	3.41E-05	Adipose_Subcutaneous
chr7_130649969_T_G_b38	rs13237881	T	G	ENSG00000158623.14	COPG2	-18678	0.674	0.095	7.55E-12	Breast
chr7_130649969_T_G_b38	rs13237881	T	G	ENSG00000158623.14	COPG2	-18678	0.234	0.048	1.32E-06	Heart_Left_Ventricle
chr7_130649969_T_G_b38	rs13237881	T	G	ENSG00000158623.14	COPG2	-18678	0.593	0.060	1.00E-21	Whole_Blood
chr7_130649969_T_G_b38	rs13237881	T	G	ENSG00000158623.14	COPG2	-18678	0.820	0.098	1.17E-14	Adrenal_Gland
chr7_130649969_T_G_b38	rs13237881	T	G	ENSG00000106484.14	MEST	158640	0.431	0.094	5.85E-06	Esophagus_Mucosa
chr7_130649969_T_G_b38	rs13237881	T	G	ENSG00000158623.14	COPG2	-18678	0.653	0.099	2.12E-10	Esophagus_Gastroesophageal_junction
chr7_130649969_T_G_b38	rs13237881	T	G	ENSG00000158623.14	COPG2	-18678	0.925	0.066	4.92E-38	Skin_Sun_Exposed_Lower_leg
chr7_130649969_T_G_b38	rs13237881	T	G	ENSG00000158623.14	COPG2	-18678	0.869	0.075	4.63E-27	Skin_Not_Sun_Exposed_Suprapubic
chr7_130649969_T_G_b38	rs13237881	T	G	ENSG00000158623.14	COPG2	-18678	0.842	0.103	1.51E-14	Pancreas
chr7_130649969_T_G_b38	rs13237881	T	G	ENSG00000158623.14	COPG2	-18678	0.570	0.065	7.60E-17	Esophagus_Muscularis
chr7_130649969_T_G_b38	rs13237881	T	G	ENSG00000158623.14	COPG2	-18678	0.671	0.104	4.87E-10	Stomach
chr7_130649969_T_G_b38	rs13237881	T	G	ENSG00000158623.14	COPG2	-18678	0.617	0.081	8.31E-13	Pituitary
chr7_130649969_T_G_b38	rs13237881	T	G	ENSG00000158623.14	COPG2	-18678	0.747	0.071	4.26E-23	Lung
chr7_130649969_T_G_b38	rs13237881	T	G	ENSG00000158623.14	COPG2	-18678	0.589	0.090	3.78E-10	Colon_Sigmoid
chr7_130649969_T_G_b38	rs13237881	T	G	ENSG00000158623.14	COPG2	-18678	0.781	0.122	2.15E-09	Small_Intestine_Terminal_Ileum
chr7_130649969_T_G_b38	rs13237881	T	G	ENSG00000158623.14	COPG2	-18678	0.763	0.077	1.73E-21	Adipose_Subcutaneous
chr7_130649969_T_G_b38	rs13237881	T	G	ENSG00000158623.14	COPG2	-18678	0.897	0.111	9.70E-14	Spleen
chr7_130649969_T_G_b38	rs13237881	T	G	ENSG00000158623.14	COPG2	-18678	0.458	0.061	3.64E-13	Nerve_Tibial
chr7_130649969_T_G_b38	rs13237881	T	G	ENSG00000158623.14	COPG2	-18678	0.492	0.082	4.77E-09	Artery_Aorta
chr7_130649969_T_G_b38	rs13237881	T	G	ENSG00000106484.14	MEST	158640	0.293	0.076	1.34E-04	Thyroid
chr7_130655148_C_A_b38	rs6467311	C	A	ENSG00000158623.14	COPG2	-13499	0.897	0.111	9.70E-14	Spleen
chr7_130655148_C_A_b38	rs6467311	C	A	ENSG00000158623.14	COPG2	-13499	0.516	0.052	1.17E-21	Artery_Tibial
chr7_130655148_C_A_b38	rs6467311	C	A	ENSG00000158623.14	COPG2	-13499	0.905	0.068	8.89E-34	Esophagus_Mucosa
chr7_130655148_C_A_b38	rs6467311	C	A	ENSG00000158623.14	COPG2	-13499	0.336	0.062	1.24E-07	Heart_Atrial_Appendage
chr7_130655148_C_A_b38	rs6467311	C	A	ENSG00000158623.14	COPG2	-13499	0.747	0.071	4.26E-23	Lung
chr7_130655148_C_A_b38	rs6467311	C	A	ENSG00000272701.2	MESTIT1	164115	0.319	0.076	3.41E-05	Adipose_Subcutaneous
chr7_130655148_C_A_b38	rs6467311	C	A	ENSG00000158623.14	COPG2	-13499	0.589	0.090	3.78E-10	Colon_Sigmoid
chr7_130655148_C_A_b38	rs6467311	C	A	ENSG00000158623.14	COPG2	-13499	0.754	0.087	2.89E-16	Colon_Transverse
chr7_130655148_C_A_b38	rs6467311	C	A	ENSG00000158623.14	COPG2	-13499	0.653	0.099	2.12E-10	Esophagus_Gastroesophageal_junction
chr7_130655148_C_A_b38	rs6467311	C	A	ENSG00000158623.14	COPG2	-13499	0.476	0.095	1.31E-06	Liver
chr7_130655148_C_A_b38	rs6467311	C	A	ENSG00000106484.14	MEST	163819	0.431	0.094	5.85E-06	Esophagus_Mucosa
chr7_130655148_C_A_b38	rs6467311	C	A	ENSG00000158623.14	COPG2	-13499	0.662	0.075	3.52E-17	Adipose_Visceral_Omentum
chr7_130655148_C_A_b38	rs6467311	C	A	ENSG00000158623.14	COPG2	-13499	0.704	0.106	3.11E-10	Prostate
chr7_130655148_C_A_b38	rs6467311	C	A	ENSG00000158623.14	COPG2	-13499	0.234	0.048	1.32E-06	Heart_Left_Ventricle
chr7_130655148_C_A_b38	rs6467311	C	A	ENSG00000158623.14	COPG2	-13499	1.166	0.088	7.24E-34	Cells_Cultured_fibroblast
chr7_130655148_C_A_b38	rs6467311	C	A	ENSG00000106484.14	MEST	163819	0.293	0.076	1.34E-04	Thyroid
chr7_130655148_C_A_b38	rs6467311	C	A	ENSG00000158623.14	COPG2	-13499	0.617	0.081	8.31E-13	Pituitary

chr7_130655148_C_A_b38	rs6467311	C	A	ENSG00000158623.14	COPG2	-13499	0.820	0.098	1.17E-14	Adrenal_Gland
chr7_130655148_C_A_b38	rs6467311	C	A	ENSG00000158623.14	COPG2	-13499	0.570	0.065	7.60E-17	Esophagus_Muscularis
chr7_130655148_C_A_b38	rs6467311	C	A	ENSG00000158623.14	COPG2	-13499	0.492	0.056	2.91E-16	Testis
chr7_130655148_C_A_b38	rs6467311	C	A	ENSG00000158623.14	COPG2	-13499	0.671	0.104	4.87E-10	Stomach
chr7_130655148_C_A_b38	rs6467311	C	A	ENSG00000158623.14	COPG2	-13499	0.925	0.066	4.92E-38	Skin_Sun_Exposed_Lower_leg
chr7_130655148_C_A_b38	rs6467311	C	A	ENSG00000158623.14	COPG2	-13499	0.781	0.122	2.15E-09	Small_Intestine_Terminal_Ileum
chr7_130655148_C_A_b38	rs6467311	C	A	ENSG00000158623.14	COPG2	-13499	0.842	0.103	1.51E-14	Pancreas
chr7_130655148_C_A_b38	rs6467311	C	A	ENSG00000158623.14	COPG2	-13499	0.718	0.049	2.86E-40	Thyroid
chr7_130655148_C_A_b38	rs6467311	C	A	ENSG00000158623.14	COPG2	-13499	0.458	0.061	3.64E-13	Nerve_Tibial
chr7_130655148_C_A_b38	rs6467311	C	A	ENSG00000158623.14	COPG2	-13499	0.492	0.082	4.77E-09	Artery_Aorta
chr7_130655148_C_A_b38	rs6467311	C	A	ENSG00000158623.14	COPG2	-13499	0.869	0.075	4.63E-27	Skin_Not_Sun_Exposed_Suprapubic
chr7_130655148_C_A_b38	rs6467311	C	A	ENSG00000158623.14	COPG2	-13499	0.763	0.077	1.73E-21	Adipose_Subcutaneous
chr7_130655148_C_A_b38	rs6467311	C	A	ENSG00000158623.14	COPG2	-13499	0.674	0.095	7.55E-12	Breast
chr7_130655148_C_A_b38	rs6467311	C	A	ENSG00000158623.14	COPG2	-13499	0.593	0.060	1.00E-21	Whole_Blood
chr8_125509988_G_A_b38	rs72657504	G	A	ENSG00000104549.11	SQLE	511491	0.512	0.128	7.82E-05	Esophagus_Muscularis

*NA=no gene symbols available, since they are novel transcripts or pseudogenes
