# Supplementary material for: Germline Polymorphisms Associated with Overall Survival in Lung Adenocarcinoma: Genome-Wide Analysis
Source: Cancers (Basel). 2024 Sep 25;16(19):3264. doi: 10.3390/cancers16193264 (PMC11475969; doi:10.3390/cancers16193264)
Supplement: Supplementary file 1 [file cancers-16-03264-s001.zip › SupplementaryTable S3.pdf]

Supplementary Table S3. eQTLs, in eQTLGen database, among the 224 SNPs associated with patient survival at P-value < 1.0 x 10^{-5}

SNP	variant_id	*P* -value	Assessed Allele	Other Allele	Zscore	Gene	Gene Symbol	distance SNP-gene	FDR
rs10164686	chr2_70940001_A_G_b38	2.26E-08	A	G	5.5909	ENSG00000124357	NAGK	-132073	1.09E-04
rs10164686	chr2_70940001_A_G_b38	7.42E-08	A	G	5.3807	ENSG00000231386	AC007395.4	128863	2.71E-04
rs10164686	chr2_70940001_A_G_b38	2.00E-40	A	G	13.3112	ENSG00000152672	CLEC4F	125378	0.00
rs10164686	chr2_70940001_A_G_b38	5.64E-06	A	G	4.5395	ENSG00000124370	MCEE	-179960	1.50E-02
rs10510759	chr3_52573272_G_A_b38	2.88E-11	G	A	-6.6527	ENSG00000163931	TKT	-667107	0.00
rs10510759	chr3_52573272_G_A_b38	1.58E-14	G	A	-7.6807	ENSG00000168273	SMIM4	16647	0.00
rs10510759	chr3_52573272_G_A_b38	1.89E-07	G	A	-5.2101	ENSG00000242142	SERBP1P3	-491415	5.88E-04
rs10510759	chr3_52573272_G_A_b38	5.16E-06	G	A	4.5582	ENSG00000163933	RFT1	-536200	1.39E-02
rs10510759	chr3_52573272_G_A_b38	4.82E-83	G	A	19.3056	ENSG00000168268	NT5DC2	43560	0.00
rs10753880	chr1_200731246_T_A_b38	2.04E-14	A	T	-7.6483	ENSG00000170128	GPR25	-142320	0.00
rs10753880	chr1_200731246_T_A_b38	3.51E-17	A	T	-8.4281	ENSG00000229191	RP11-168O16.1	-295124	0.00
rs10753880	chr1_200731246_T_A_b38	1.44E-05	A	T	-4.3374	ENSG00000163362	C1orf106	-172145	3.69E-02
rs10753880	chr1_200731246_T_A_b38	9.84E-52	A	T	-15.1328	ENSG00000118197	DDX59	84314	0.00
rs10753880	chr1_200731246_T_A_b38	6.93E-121	A	T	23.3794	ENSG00000118200	CAMSAP2	-68885	0.00
rs10762653	chr10_75078296_G_A_b38	7.16E-22	G	A	9.6115	ENSG00000185009	AP3M1	941882	0.00
rs10762653	chr10_75078296_G_A_b38	4.57E-08	G	A	-5.4671	ENSG00000165637	VDAC2	-142505	1.52E-04
rs10762653	chr10_75078296_G_A_b38	3.41E-115	G	A	22.8131	ENSG00000156110	ADK	648044	0.00
rs10762653	chr10_75078296_G_A_b38	1.69E-07	G	A	-5.2304	ENSG00000165655	ZNF503	-321572	5.26E-04
rs10762653	chr10_75078296_G_A_b38	3.53E-07	G	A	-5.0926	ENSG00000148655	C10orf11	-917514	1.07E-03
rs10954274	chr7_130649535_C_A_b38	3.27E-310	A	C	-42.1154	ENSG00000158623	COPG2	84534	0.00
rs10954274	chr7_130649535_C_A_b38	8.60E-14	A	C	-7.4607	ENSG00000106484	MEST	198288	0.00
rs112824891	chr3_52740997_G_A_b38	1.39E-07	A	G	-5.2659	ENSG00000242142	SERBP1P3	-323690	4.27E-04
rs112824891	chr3_52740997_G_A_b38	1.68E-09	A	G	-6.0263	ENSG00000163931	TKT	-499382	1.31E-05
rs112824891	chr3_52740997_G_A_b38	2.69E-65	A	G	17.0652	ENSG00000168268	NT5DC2	211285	0.00
rs112824891	chr3_52740997_G_A_b38	2.24E-13	A	G	-7.3337	ENSG00000168273	SMIM4	184372	0.00
rs113071416	chr3_52764132_G_A_b38	1.10E-07	G	A	-5.3101	ENSG00000163931	TKT	-476247	3.46E-04
rs113071416	chr3_52764132_G_A_b38	1.16E-09	G	A	-6.0857	ENSG00000055955	ITIH4	-58095	1.31E-05
rs113071416	chr3_52764132_G_A_b38	5.10E-25	G	A	10.3312	ENSG00000168268	NT5DC2	234420	0.00

rs113071416	chr3_52764132_G_A_b38	2.20E-11	G	A	-6.6916	ENSG00000168273	SMIM4	207507	0.00
rs113071416	chr3_52764132_G_A_b38	1.65E-06	G	A	4.7925	ENSG00000163933	RFT1	-345340	4.52E-03
rs113071416	chr3_52764132_G_A_b38	1.30E-07	G	A	-5.2788	ENSG00000242142	SERBP1P3	-300555	3.96E-04
rs113528713	chr3_52727078_G_A_b38	1.42E-07	A	G	-5.262	ENSG00000242142	SERBP1P3	-337609	4.33E-04
rs113528713	chr3_52727078_G_A_b38	2.63E-65	A	G	17.0667	ENSG00000168268	NT5DC2	197366	0.00
rs113528713	chr3_52727078_G_A_b38	2.35E-13	A	G	-7.3273	ENSG00000168273	SMIM4	170453	0.00
rs113528713	chr3_52727078_G_A_b38	1.32E-09	A	G	-6.065	ENSG00000163931	TKT	-513301	1.31E-05
rs114577020	chr3_52572154_T_C_b38	1.79E-69	C	T	17.6181	ENSG00000168268	NT5DC2	42442	0.00
rs114577020	chr3_52572154_T_C_b38	4.93E-06	C	T	4.5679	ENSG00000163933	RFT1	-537318	1.33E-02
rs114577020	chr3_52572154_T_C_b38	5.72E-11	C	T	-6.5508	ENSG00000163931	TKT	-668225	0.00
rs114577020	chr3_52572154_T_C_b38	1.09E-07	C	T	-5.3113	ENSG00000242142	SERBP1P3	-492533	3.40E-04
rs114577020	chr3_52572154_T_C_b38	5.51E-11	C	T	-6.5564	ENSG00000168273	SMIM4	15529	0.00
rs114687381	chr3_52731933_G_A_b38	1.06E-07	A	G	-5.3152	ENSG00000242142	SERBP1P3	-332754	3.33E-04
rs114687381	chr3_52731933_G_A_b38	1.26E-10	A	G	-6.4318	ENSG00000163931	TKT	-508446	0.00
rs114687381	chr3_52731933_G_A_b38	2.57E-12	A	G	-6.9995	ENSG00000168273	SMIM4	175308	0.00
rs114687381	chr3_52731933_G_A_b38	4.78E-69	A	G	17.5625	ENSG00000168268	NT5DC2	202221	0.00
rs114687381	chr3_52731933_G_A_b38	9.16E-06	A	G	4.436	ENSG00000163933	RFT1	-377539	2.39E-02
rs114707129	chr3_52750191_G_A_b38	2.85E-11	G	A	-6.654	ENSG00000163931	TKT	-490188	0.00
rs114707129	chr3_52750191_G_A_b38	1.17E-68	G	A	17.5117	ENSG00000168268	NT5DC2	220479	0.00
rs114707129	chr3_52750191_G_A_b38	6.25E-08	G	A	-5.4115	ENSG00000242142	SERBP1P3	-314496	2.47E-04
rs114707129	chr3_52750191_G_A_b38	8.47E-06	G	A	4.4531	ENSG00000163933	RFT1	-359281	2.20E-02
rs114707129	chr3_52750191_G_A_b38	3.75E-11	G	A	-6.6135	ENSG00000168273	SMIM4	193566	0.00
rs115801090	chr3_52762646_G_A_b38	6.11E-08	A	G	-5.4154	ENSG00000242142	SERBP1P3	-302041	2.47E-04
rs115801090	chr3_52762646_G_A_b38	3.70E-11	A	G	-6.6157	ENSG00000168273	SMIM4	206021	0.00
rs115801090	chr3_52762646_G_A_b38	2.57E-68	A	G	17.4668	ENSG00000168268	NT5DC2	232934	0.00
rs115801090	chr3_52762646_G_A_b38	8.10E-06	A	G	4.4626	ENSG00000163933	RFT1	-346826	2.12E-02
rs115801090	chr3_52762646_G_A_b38	9.55E-12	A	G	-6.8132	ENSG00000163931	TKT	-477733	0.00
rs116493126	chr3_52716309_T_C_b38	7.04E-69	C	T	17.5404	ENSG00000168268	NT5DC2	186597	0.00
rs116493126	chr3_52716309_T_C_b38	3.04E-11	C	T	-6.6446	ENSG00000168273	SMIM4	159684	0.00
rs116493126	chr3_52716309_T_C_b38	7.77E-06	C	T	4.4714	ENSG00000163933	RFT1	-393163	2.04E-02
rs116493126	chr3_52716309_T_C_b38	3.55E-11	C	T	-6.6218	ENSG00000163931	TKT	-524070	0.00
rs116493126	chr3_52716309_T_C_b38	7.33E-08	C	T	-5.3828	ENSG00000242142	SERBP1P3	-348378	2.65E-04

rs116578433	chr3_13566151_G_A_b38	1.65E-06	G	A	-4.7917	ENSG00000124523	SIRT5	-27292	4.53E-03
rs117225132	chr10_26244381_G_A_b38	7.90E-06	A	G	4.4679	ENSG00000077420	APBB1IP	-258622	2.07E-02
rs117482124	chr15_57093760_T_C_b38	3.13E-07	T	C	5.1155	ENSG00000247982	RP11-358M11.2	-210303	9.63E-04
rs12107484	chr3_52598077_T_C_b38	1.61E-09	C	T	-6.0326	ENSG00000163931	TKT	-642302	1.31E-05
rs12107484	chr3_52598077_T_C_b38	9.62E-75	C	T	18.2919	ENSG00000168268	NT5DC2	68365	0.00
rs12107484	chr3_52598077_T_C_b38	1.89E-07	C	T	-5.2101	ENSG00000242142	SERBP1P3	-466610	5.88E-04
rs12107484	chr3_52598077_T_C_b38	1.53E-14	C	T	-7.6846	ENSG00000168273	SMIM4	41452	0.00
rs12107484	chr3_52598077_T_C_b38	1.42E-05	C	T	4.3412	ENSG00000163933	RFT1	-511395	3.64E-02
rs12240362	chr10_89827140_T_C_b38	3.27E-310	T	C	40.3553	ENSG00000232229	RP11-248C1.2	-8045	0.00
rs12240362	chr10_89827140_T_C_b38	1.23E-08	T	C	-5.6955	ENSG00000152778	IFIT5	409347	6.43E-05
rs12240362	chr10_89827140_T_C_b38	1.65E-43	T	C	13.8314	ENSG00000152782	PANK1	212917	0.00
rs12240362	chr10_89827140_T_C_b38	3.17E-14	T	C	7.5912	ENSG00000107798	LIPA	513077	0.00
rs12240362	chr10_89827140_T_C_b38	1.11E-59	T	C	16.2928	ENSG00000138182	KIF20B	88864	0.00
rs12265484	chr10_89824818_G_A_b38	8.80E-60	A	G	16.3071	ENSG00000138182	KIF20B	86542	0.00
rs12265484	chr10_89824818_G_A_b38	3.27E-310	A	G	40.8426	ENSG00000232229	RP11-248C1.2	-10367	0.00
rs12265484	chr10_89824818_G_A_b38	1.86E-13	A	G	7.3584	ENSG00000107798	LIPA	510755	0.00
rs12265484	chr10_89824818_G_A_b38	8.24E-47	A	G	14.3679	ENSG00000152782	PANK1	210595	0.00
rs12265484	chr10_89824818_G_A_b38	8.96E-10	A	G	-6.1269	ENSG00000152778	IFIT5	407025	1.32E-05
rs13000315	chr2_70945905_A_T_b38	1.50E-05	A	T	4.3287	ENSG00000116031	CD207	112886	3.82E-02
rs13000315	chr2_70945905_A_T_b38	4.30E-10	A	T	6.2428	ENSG00000124357	NAGK	-126169	0.00
rs13000315	chr2_70945905_A_T_b38	5.08E-53	A	T	15.3267	ENSG00000152672	CLEC4F	131282	0.00
rs13000315	chr2_70945905_A_T_b38	3.74E-06	A	T	4.6255	ENSG00000124370	MCEE	-174056	1.00E-02
rs13000315	chr2_70945905_A_T_b38	2.60E-09	A	T	5.9549	ENSG00000231386	AC007395.4	134767	2.61E-05
rs13022751	chr2_70938233_T_C_b38	1.85E-06	T	C	4.7697	ENSG00000124370	MCEE	-181728	5.02E-03
rs13022751	chr2_70938233_T_C_b38	8.67E-50	T	C	14.8353	ENSG00000152672	CLEC4F	123610	0.00
rs13022751	chr2_70938233_T_C_b38	5.46E-08	T	C	5.436	ENSG00000231386	AC007395.4	127095	2.03E-04
rs13022751	chr3_70938233_T_C_b38	2.62E-08	T	C	5.5656	ENSG00000124357	NAGK	-133841	1.15E-04
rs13237881	chr7_130649969_T_G_b38	3.27E-310	T	G	-40.4162	ENSG00000158623	COPG2	84968	0.00
rs13237881	chr7_130649969_T_G_b38	2.24E-12	T	G	-7.0182	ENSG00000106484	MEST	198722	0.00
rs138689441	chr2_70932225_A_G_b38	4.68E-09	A	G	5.8582	ENSG00000231386	AC007395.4	121087	3.24E-05
rs138689441	chr2_70932225_A_G_b38	5.05E-72	A	G	17.9473	ENSG00000152672	CLEC4F	117602	0.00
rs138689441	chr2_70932225_A_G_b38	5.29E-08	A	G	5.4415	ENSG00000124357	NAGK	-139849	1.90E-04

rs138689441	chr2_70932225_A_G_b38	8.74E-07	A	G	4.9181	ENSG00000116031	CD207	99206	2.56E-03
rs140728965	chr3_52709131_T_C_b38	7.59E-06	T	C	4.4765	ENSG00000163933	RFT1	-400341	2.00E-02
rs140728965	chr3_52709131_T_C_b38	3.86E-69	T	C	17.5747	ENSG00000168268	NT5DC2	179419	0.00
rs140728965	chr3_52709131_T_C_b38	7.77E-08	T	C	-5.3723	ENSG00000242142	SERBP1P3	-355556	2.78E-04
rs140728965	chr3_52709131_T_C_b38	2.38E-11	T	C	-6.6804	ENSG00000168273	SMIM4	152506	0.00
rs140728965	chr3_52709131_T_C_b38	3.84E-11	T	C	-6.6101	ENSG00000163931	TKT	-531248	0.00
rs140892559	chr3_105896971_T_A_b38	2.40E-16	T	A	-8.2002	ENSG00000114423	CBLB	134468	0.00
rs140892559	chr3_105896971_T_A_b38	1.36E-13	T	A	7.4002	ENSG00000170017	ALCAM	425070	0.00
rs148578765	chr15_57254048_G_A_b38	4.99E-07	G	A	5.0267	ENSG00000247982	RP11-358M11.2	-50015	1.54E-03
rs151032151	chr3_52741024_G_A_b38	7.06E-13	A	G	-7.1781	ENSG00000168273	SMIM4	184399	0.00
rs151032151	chr3_52741024_G_A_b38	1.22E-05	A	G	4.3734	ENSG00000163933	RFT1	-368448	3.17E-02
rs151032151	chr3_52741024_G_A_b38	2.03E-74	A	G	18.251	ENSG00000168268	NT5DC2	211312	0.00
rs151032151	chr3_52741024_G_A_b38	3.27E-10	A	G	-6.2854	ENSG00000163931	TKT	-499355	0.00
rs151032151	chr3_52741024_G_A_b38	9.69E-08	A	G	-5.3324	ENSG00000242142	SERBP1P3	-323663	3.15E-04
rs151212827	chr3_52957703_A_G_b38	1.08E-11	A	G	-6.7955	ENSG00000163931	TKT	-282676	0.00
rs151212827	chr3_52957703_A_G_b38	1.57E-47	A	G	14.4822	ENSG00000168268	NT5DC2	427991	0.00
rs151212827	chr3_52957703_A_G_b38	2.76E-08	A	G	-5.5562	ENSG00000168273	SMIM4	401078	1.21E-04
rs17127746	chr10_89823936_T_C_b38	3.27E-310	T	C	40.808	ENSG00000232229	RP11-248C1.2	-11249	0.00
rs17127746	chr10_89823936_T_C_b38	8.05E-10	T	C	-6.144	ENSG00000152778	IFIT5	406143	1.32E-05
rs17127746	chr10_89823936_T_C_b38	2.71E-60	T	C	16.379	ENSG00000138182	KIF20B	85660	0.00
rs17127746	chr10_89823936_T_C_b38	1.45E-13	T	C	7.392	ENSG00000107798	LIPA	509873	0.00
rs17127746	chr10_89823936_T_C_b38	9.38E-47	T	C	14.359	ENSG00000152782	PANK1	209713	0.00
rs181612199	chr3_52745959_T_C_b38	6.25E-08	C	T	-5.4115	ENSG00000242142	SERBP1P3	-318728	2.47E-04
rs181612199	chr3_52745959_T_C_b38	3.67E-11	C	T	-6.6169	ENSG00000168273	SMIM4	189334	0.00
rs181612199	chr3_52745959_T_C_b38	3.48E-11	C	T	-6.6244	ENSG00000163931	TKT	-494420	0.00
rs181612199	chr3_52745959_T_C_b38	8.59E-06	C	T	4.4501	ENSG00000163933	RFT1	-363513	2.23E-02
rs181612199	chr3_52745959_T_C_b38	7.72E-69	C	T	17.5353	ENSG00000168268	NT5DC2	216247	0.00
rs185761183	chr3_52773429_T_C_b38	2.78E-11	C	T	-6.6578	ENSG00000163931	TKT	-466950	0.00
rs185761183	chr3_52773429_T_C_b38	2.54E-08	C	T	-5.5701	ENSG00000242142	SERBP1P3	-291258	1.15E-04
rs185761183	chr3_52773429_T_C_b38	1.31E-12	C	T	-7.0927	ENSG00000168273	SMIM4	216804	0.00
rs185761183	chr3_52773429_T_C_b38	8.75E-06	C	T	4.446	ENSG00000163933	RFT1	-336043	2.28E-02
rs185761183	chr3_52773429_T_C_b38	2.38E-70	C	T	17.7319	ENSG00000168268	NT5DC2	243717	0.00

rs186146073	chr3_52569345_G_C_b38	7.87E-11	C	G	-6.5029	ENSG00000168273	SMIM4	12720	
rs186146073	chr3_52569345_G_C_b38	9.31E-70	C	G	17.6552	ENSG00000168268	NT5DC2	39633	0.00
rs186146073	chr3_52569345_G_C_b38	4.76E-06	C	G	4.575	ENSG00000163933	RFT1	-540127	1.28E-02
rs186146073	chr3_52569345_G_C_b38	3.67E-08	C	G	-5.5059	ENSG00000242142	SERBP1P3	-495342	1.40E-04
rs186146073	chr3_52569345_G_C_b38	1.28E-10	C	G	-6.429	ENSG00000163931	TKT	-671034	0.00
rs186956810	chr3_53033317_T_C_b38	1.81E-44	T	C	13.9892	ENSG00000168268	NT5DC2	503605	0.00
rs186956810	chr3_53033317_T_C_b38	7.97E-13	T	C	-7.1614	ENSG00000163931	TKT	-207062	0.00
rs186956810	chr3_53033317_T_C_b38	3.93E-07	T	C	-5.0721	ENSG00000168273	SMIM4	476692	1.15E-03
rs188074306	chr3_52677576_T_C_b38	6.58E-06	T	C	4.5071	ENSG00000163933	RFT1	-431896	1.74E-02
rs188074306	chr3_52677576_T_C_b38	3.36E-69	T	C	17.5825	ENSG00000168268	NT5DC2	147864	0.00
rs188074306	chr3_52677576_T_C_b38	9.88E-08	T	C	-5.3289	ENSG00000242142	SERBP1P3	-387111	3.21E-04
rs188074306	chr3_52677576_T_C_b38	3.07E-11	T	C	-6.6433	ENSG00000163931	TKT	-562803	0.00
rs188074306	chr3_52677576_T_C_b38	1.70E-11	T	C	-6.73	ENSG00000168273	SMIM4	120951	0.00
rs190297652	chr3_52749370_G_A_b38	2.50E-08	A	G	-5.5735	ENSG00000242142	SERBP1P3	-315317	1.15E-04
rs190297652	chr3_52749370_G_A_b38	3.14E-43	A	G	13.7851	ENSG00000168268	NT5DC2	219658	0.00
rs190297652	chr3_52749370_G_A_b38	1.00E-08	A	G	-5.7299	ENSG00000163931	TKT	-491009	5.80E-05
rs190297652	chr3_52749370_G_A_b38	1.62E-10	A	G	-6.3938	ENSG00000168273	SMIM4	192745	0.00
rs191101411	chr3_52768817_G_C_b38	1.89E-70	G	C	17.7449	ENSG00000168268	NT5DC2	239105	0.00
rs191101411	chr3_52768817_G_C_b38	2.46E-08	G	C	-5.5759	ENSG00000242142	SERBP1P3	-295870	1.15E-04
rs191101411	chr3_52768817_G_C_b38	8.86E-06	G	C	4.4433	ENSG00000163933	RFT1	-340655	2.32E-02
rs191101411	chr3_52768817_G_C_b38	2.73E-11	G	C	-6.6605	ENSG00000163931	TKT	-471562	0.00
rs191101411	chr3_52768817_G_C_b38	1.57E-12	G	C	-7.0681	ENSG00000168273	SMIM4	212192	0.00
rs192710451	chr3_52753086_G_A_b38	6.43E-13	A	G	-7.1909	ENSG00000168273	SMIM4	196461	0.00
rs192710451	chr3_52753086_G_A_b38	1.78E-06	A	G	-4.7764	ENSG00000163931	TKT	-487293	4.93E-03
rs192710451	chr3_52753086_G_A_b38	6.45E-49	A	G	14.7001	ENSG00000168268	NT5DC2	223374	0.00
rs1987234	chr3_52564343_T_C_b38	1.88E-14	C	T	-7.6583	ENSG00000168273	SMIM4	7718	0.00
rs1987234	chr3_52564343_T_C_b38	1.61E-09	C	T	-6.0326	ENSG00000163931	TKT	-676036	1.31E-05
rs1987234	chr3_52564343_T_C_b38	1.43E-05	C	T	4.3387	ENSG00000163933	RFT1	-545129	3.67E-02
rs1987234	chr3_52564343_T_C_b38	1.89E-07	C	T	-5.2101	ENSG00000242142	SERBP1P3	-500344	5.88E-04
rs1987234	chr3_52564343_T_C_b38	3.52E-74	C	T	18.221	ENSG00000168268	NT5DC2	34631	0.00
rs34195894	chr2_70936224_C_G_b38	8.28E-09	C	G	5.7627	ENSG00000124357	NAGK	-135850	5.81E-05
rs34195894	chr2_70936224_C_G_b38	1.59E-06	C	G	4.7999	ENSG00000116031	CD207	103205	4.39E-03

rs34195894	chr2_70936224_C_G_b38	7.63E-10	C	G	6.1525	ENSG00000231386	AC007395.4	125086	1.32E-05
rs34195894	chr2_70936224_C_G_b38	5.12E-72	C	G	17.9465	ENSG00000152672	CLEC4F	121601	0.00
rs34315744	chr2_70930495_A_C_b38	9.19E-10	A	C	6.1229	ENSG00000231386	AC007395.4	119357	1.32E-05
rs34315744	chr2_70930495_A_C_b38	5.03E-07	A	C	5.0251	ENSG00000116031	CD207	97476	1.54E-03
rs34315744	chr2_70930495_A_C_b38	5.08E-08	A	C	5.4485	ENSG00000124357	NAGK	-141579	1.77E-04
rs34315744	chr2_70930495_A_C_b38	1.32E-72	A	C	18.0216	ENSG00000152672	CLEC4F	115872	0.00
rs34456496	chr2_70921463_T_C_b38	2.75E-75	T	C	18.3602	ENSG00000152672	CLEC4F	106840	0.00
rs34456496	chr2_70921463_T_C_b38	6.89E-08	T	C	5.3941	ENSG00000116031	CD207	88444	2.53E-04
rs34456496	chr2_70921463_T_C_b38	1.20E-07	T	C	5.294	ENSG00000124357	NAGK	-150611	3.71E-04
rs34456496	chr2_70921463_T_C_b38	3.68E-09	T	C	5.8981	ENSG00000231386	AC007395.4	110325	2.60E-05
rs34566971	chr2_70918144_C_T_b38	4.13E-09	C	T	5.879	ENSG00000231386	AC007395.4	107006	3.24E-05
rs34566971	chr2_70918144_C_T_b38	1.75E-07	C	T	5.2245	ENSG00000124357	NAGK	-153930	5.45E-04
rs34566971	chr2_70918144_C_T_b38	6.98E-08	C	T	5.3919	ENSG00000116031	CD207	85125	2.59E-04
rs34566971	chr2_70918144_C_T_b38	6.14E-76	C	T	18.4414	ENSG00000152672	CLEC4F	103521	0.00
rs34710884	chr17_45186544_G_A_b38	5.02E-12	A	G	-6.9048	ENSG00000108883	EFTUD2	311741	0.00
rs34888497	chr2_70940022_A_G_b38	1.99E-45	A	G	14.1455	ENSG00000152672	CLEC4F	125399	0.00
rs34888497	chr2_70940022_A_G_b38	1.18E-06	A	G	4.8593	ENSG00000124370	MCEE	-179939	3.31E-03
rs34888497	chr2_70940022_A_G_b38	7.71E-06	A	G	4.4732	ENSG00000116031	CD207	107003	2.02E-02
rs34888497	chr2_70940022_A_G_b38	1.53E-07	A	G	5.2495	ENSG00000231386	AC007395.4	128884	4.83E-04
rs34888497	chr2_70940022_A_G_b38	1.47E-08	A	G	5.6649	ENSG00000124357	NAGK	-132052	7.06E-05
rs34965400	chr2_70936147_A_G_b38	5.72E-72	A	G	17.9403	ENSG00000152672	CLEC4F	121524	0.00
rs34965400	chr2_70936147_A_G_b38	1.22E-08	A	G	5.6976	ENSG00000124357	NAGK	-135927	6.43E-05
rs34965400	chr2_70936147_A_G_b38	1.25E-06	A	G	4.8484	ENSG00000116031	CD207	103128	3.55E-03
rs34965400	chr2_70936147_A_G_b38	8.42E-10	A	G	6.1369	ENSG00000231386	AC007395.4	125009	1.32E-05
rs35096861	chr2_70935278_T_C_b38	8.51E-07	T	C	4.9233	ENSG00000116031	CD207	102259	2.50E-03
rs35096861	chr2_70935278_T_C_b38	1.91E-08	T	C	5.6202	ENSG00000124357	NAGK	-136796	1.02E-04
rs35096861	chr2_70935278_T_C_b38	3.57E-72	T	C	17.9666	ENSG00000152672	CLEC4F	120655	0.00
rs35096861	chr2_70935278_T_C_b38	1.18E-09	T	C	6.0827	ENSG00000231386	AC007395.4	124140	1.31E-05
rs35494197	chr10_89823973_T_C_b38	1.23E-13	T	C	7.4142	ENSG00000107798	LIPA	509910	0.00
rs35494197	chr10_89823973_T_C_b38	8.01E-10	T	C	-6.1446	ENSG00000152778	IFIT5	406180	1.32E-05
rs35494197	chr10_89823973_T_C_b38	7.62E-47	T	C	14.3733	ENSG00000152782	PANK1	209750	0.00
rs35494197	chr10_89823973_T_C_b38	2.49E-60	T	C	16.3841	ENSG00000138182	KIF20B	85697	0.00

rs35494197	chr10_89823973_T_C_b38	3.27E-310	T	C	40.8428	ENSG00000232229	RP11-248C1.2	-11212	0.00
rs35768563	chr2_70936090_T_C_b38	1.19E-09	T	C	6.0816	ENSG00000231386	AC007395.4	124952	1.31E-05
rs35768563	chr2_70936090_T_C_b38	8.56E-72	T	C	17.9179	ENSG00000152672	CLEC4F	121467	0.00
rs35768563	chr2_70936090_T_C_b38	1.04E-06	T	C	4.8847	ENSG00000116031	CD207	103071	2.95E-03
rs35768563	chr2_70936090_T_C_b38	1.05E-08	T	C	5.7224	ENSG00000124357	NAGK	-135984	5.79E-05
rs35791459	chr2_70930960_C_T_b38	9.96E-10	C	T	6.1101	ENSG00000231386	AC007395.4	119822	1.32E-05
rs35791459	chr2_70930960_C_T_b38	6.88E-07	C	T	4.9649	ENSG00000116031	CD207	97941	1.99E-03
rs35791459	chr2_70930960_C_T_b38	1.07E-73	C	T	18.1604	ENSG00000152672	CLEC4F	116337	0.00
rs35791459	chr2_70930960_C_T_b38	5.00E-08	C	T	5.4516	ENSG00000124357	NAGK	-141114	1.77E-04
rs35921308	chr2_70930710_T_A_b38	4.82E-08	T	A	5.4581	ENSG00000124357	NAGK	-141364	1.65E-04
rs35921308	chr2_70930710_T_A_b38	9.62E-10	T	A	6.1156	ENSG00000231386	AC007395.4	119572	1.32E-05
rs35921308	chr2_70930710_T_A_b38	3.01E-72	T	A	17.9759	ENSG00000152672	CLEC4F	116087	0.00
rs35921308	chr2_70930710_T_A_b38	5.94E-07	T	A	4.9932	ENSG00000116031	CD207	97691	1.79E-03
rs36094120	chr15_91470160_T_C_b38	1.26E-12	T	C	-7.0989	ENSG00000176463	SLCO3A1	-542905	0.00
rs36094120	chr15_91470160_T_C_b38	8.04E-13	T	C	-7.1603	ENSG00000258551	RP11-661P17.1	-36799	0.00
rs4589733	chr2_38763253_G_A_b38	2.20E-16	G	A	-8.2104	ENSG00000143891	GALM	59680	0.00
rs4589733	chr2_38763253_G_A_b38	7.91E-21	G	A	-9.3608	ENSG00000163214	DHX57	-73578	0.00
rs4776284	chr15_66156559_C_A_b38	5.19E-20	A	C	9.1602	ENSG00000166938	DIS3L	-156998	0.00
rs4933160	chr10_89824354_T_C_b38	3.27E-310	C	T	40.816	ENSG00000232229	RP11-248C1.2	-10831	0.00
rs4933160	chr10_89824354_T_C_b38	9.66E-47	C	T	14.3569	ENSG00000152782	PANK1	210131	0.00
rs4933160	chr10_89824354_T_C_b38	4.91E-10	C	T	-6.2219	ENSG00000152778	IFIT5	406561	0.00
rs4933160	chr10_89824354_T_C_b38	1.15E-13	C	T	7.4228	ENSG00000107798	LIPA	510291	0.00
rs4933160	chr10_89824354_T_C_b38	1.21E-59	C	T	16.2877	ENSG00000138182	KIF20B	86078	0.00
rs4933161	chr10_89824422_T_G_b38	8.41E-47	G	T	14.3665	ENSG00000152782	PANK1	210199	0.00
rs4933161	chr10_89824422_T_G_b38	3.27E-310	G	T	40.8279	ENSG00000232229	RP11-248C1.2	-10763	0.00
rs4933161	chr10_89824422_T_G_b38	1.87E-13	G	T	7.3581	ENSG00000107798	LIPA	510359	0.00
rs4933161	chr10_89824422_T_G_b38	1.80E-60	G	T	16.4036	ENSG00000138182	KIF20B	86146	0.00
rs4933161	chr10_89824422_T_G_b38	9.78E-10	G	T	-6.113	ENSG00000152778	IFIT5	406629	1.32E-05
rs4933514	chr10_89824476_T_C_b38	1.24E-13	C	T	7.4126	ENSG00000107798	LIPA	510413	0.00
rs4933514	chr10_89824476_T_C_b38	3.04E-47	C	T	14.437	ENSG00000152782	PANK1	210253	0.00
rs4933514	chr10_89824476_T_C_b38	8.57E-60	C	T	16.3087	ENSG00000138182	KIF20B	86200	0.00
rs4933514	chr10_89824476_T_C_b38	8.55E-10	C	T	-6.1343	ENSG00000152778	IFIT5	406683	1.32E-05

rs4933514	chr10_89824476_T_C_b38	3.27E-310	C	T	40.8355	ENSG00000232229	RP11-248C1.2	-10709	0.00
rs56156188	chr3_52696683_T_G_b38	8.00E-74	G	T	18.1762	ENSG00000168268	NT5DC2	166971	0.00
rs56156188	chr3_52696683_T_G_b38	1.54E-09	G	T	-6.0406	ENSG00000163931	TKT	-543696	1.31E-05
rs56156188	chr3_52696683_T_G_b38	1.73E-05	G	T	4.2969	ENSG00000163933	RFT1	-412789	4.37E-02
rs56156188	chr3_52696683_T_G_b38	1.87E-07	G	T	-5.2117	ENSG00000242142	SERBP1P3	-368004	5.88E-04
rs56156188	chr3_52696683_T_G_b38	2.98E-14	G	T	-7.5991	ENSG00000168273	SMIM4	140058	0.00
rs56259931	chr3_52540349_T_C_b38	1.65E-07	C	T	-5.2353	ENSG00000242142	SERBP1P3	-524338	5.14E-04
rs56259931	chr3_52540349_T_C_b38	1.01E-05	C	T	4.4149	ENSG00000163933	RFT1	-569123	2.62E-02
rs56259931	chr3_52540349_T_C_b38	1.54E-74	C	T	18.2663	ENSG00000168268	NT5DC2	10637	0.00
rs56259931	chr3_52540349_T_C_b38	5.70E-09	C	T	-5.8251	ENSG00000163931	TKT	-700030	3.88E-05
rs56259931	chr3_52540349_T_C_b38	2.77E-14	C	T	-7.6088	ENSG00000168273	SMIM4	-16276	0.00
rs56276589	chr3_52738182_T_C_b38	2.14E-13	T	C	-7.3395	ENSG00000168273	SMIM4	181557	0.00
rs56276589	chr3_52738182_T_C_b38	1.46E-07	T	C	-5.2577	ENSG00000242142	SERBP1P3	-326505	4.51E-04
rs56276589	chr3_52738182_T_C_b38	1.62E-09	T	C	-6.0324	ENSG00000163931	TKT	-502197	1.31E-05
rs56276589	chr3_52738182_T_C_b38	2.15E-65	T	C	17.0786	ENSG00000168268	NT5DC2	208470	0.00
rs56354394	chr2_70933937_A_G_b38	5.24E-06	A	G	4.5552	ENSG00000124370	MCEE	-186024	1.41E-02
rs56354394	chr2_70933937_A_G_b38	5.91E-229	A	G	32.3053	ENSG00000152672	CLEC4F	119314	0.00
rs56354394	chr2_70933937_A_G_b38	6.92E-07	A	G	4.9637	ENSG00000116031	CD207	100918	2.01E-03
rs56354394	chr2_70933937_A_G_b38	9.07E-15	A	G	7.7516	ENSG00000124357	NAGK	-138137	0.00
rs56354394	chr2_70933937_A_G_b38	4.96E-28	A	G	10.9764	ENSG00000231386	AC007395.4	122799	0.00
rs56837675	chr3_52716277_T_C_b38	1.66E-05	T	C	4.3067	ENSG00000163933	RFT1	-393195	4.19E-02
rs56837675	chr3_52716277_T_C_b38	1.32E-07	T	C	-5.2754	ENSG00000242142	SERBP1P3	-348410	4.08E-04
rs56837675	chr3_52716277_T_C_b38	7.83E-72	T	C	17.9229	ENSG00000168268	NT5DC2	186565	0.00
rs56837675	chr3_52716277_T_C_b38	1.43E-14	T	C	-7.6935	ENSG00000168273	SMIM4	159652	0.00
rs56837675	chr3_52716277_T_C_b38	7.85E-10	T	C	-6.148	ENSG00000163931	TKT	-524102	1.32E-05
rs57008939	chr3_52677354_T_C_b38	3.63E-14	T	C	-7.5735	ENSG00000168273	SMIM4	120729	0.00
rs57008939	chr3_52677354_T_C_b38	4.81E-74	T	C	18.2039	ENSG00000168268	NT5DC2	147642	0.00
rs57008939	chr3_52677354_T_C_b38	2.04E-07	T	C	-5.1952	ENSG00000242142	SERBP1P3	-387333	6.25E-04
rs57008939	chr3_52677354_T_C_b38	1.61E-09	T	C	-6.0326	ENSG00000163931	TKT	-563025	1.31E-05
rs57319306	chr3_52541235_G_C_b38	1.65E-07	C	G	-5.2353	ENSG00000242142	SERBP1P3	-523452	5.14E-04
rs57319306	chr3_52541235_G_C_b38	1.01E-05	C	G	4.4149	ENSG00000163933	RFT1	-568237	2.62E-02
rs57319306	chr3_52541235_G_C_b38	2.54E-14	C	G	-7.6197	ENSG00000168273	SMIM4	-15390	0.00

rs57319306	chr3_52541235_G_C_b38	2.48E-74	C	G	18.2402	ENSG00000168268	NT5DC2	11523	0.00
rs57319306	chr3_52541235_G_C_b38	5.48E-09	C	G	-5.8317	ENSG00000163931	TKT	-699144	3.88E-05
rs57560655	chr3_52543318_G_A_b38	8.82E-06	A	G	4.4443	ENSG00000163933	RFT1	-566154	2.30E-02
rs57560655	chr3_52543318_G_A_b38	2.22E-14	A	G	-7.6371	ENSG00000168273	SMIM4	-13307	0.00
rs57560655	chr3_52543318_G_A_b38	5.48E-09	A	G	-5.8317	ENSG00000163931	TKT	-697061	3.88E-05
rs57560655	chr3_52543318_G_A_b38	1.76E-07	A	G	-5.2227	ENSG00000242142	SERBP1P3	-521369	5.51E-04
rs57560655	chr3_52543318_G_A_b38	2.89E-74	A	G	18.2319	ENSG00000168268	NT5DC2	13606	0.00
rs57757646	chr3_52602792_T_C_b38	1.89E-07	T	C	-5.2101	ENSG00000242142	SERBP1P3	-461895	5.88E-04
rs57757646	chr3_52602792_T_C_b38	1.13E-74	T	C	18.2833	ENSG00000168268	NT5DC2	73080	0.00
rs57757646	chr3_52602792_T_C_b38	1.42E-05	T	C	4.3412	ENSG00000163933	RFT1	-506680	3.64E-02
rs57757646	chr3_52602792_T_C_b38	1.41E-14	T	C	-7.6956	ENSG00000168273	SMIM4	46167	0.00
rs57757646	chr3_52602792_T_C_b38	1.61E-09	T	C	-6.0326	ENSG00000163931	TKT	-637587	1.31E-05
rs57946083	chr3_52721570_G_A_b38	2.65E-65	G	A	17.0662	ENSG00000168268	NT5DC2	191858	0.00
rs57946083	chr3_52721570_G_A_b38	1.54E-13	G	A	-7.3833	ENSG00000168273	SMIM4	164945	0.00
rs57946083	chr3_52721570_G_A_b38	1.41E-07	G	A	-5.2635	ENSG00000242142	SERBP1P3	-343117	4.33E-04
rs57946083	chr3_52721570_G_A_b38	1.54E-09	G	A	-6.04	ENSG00000163931	TKT	-518809	1.31E-05
rs58055098	chr10_89829386_G_C_b38	6.72E-94	C	G	20.5566	ENSG00000232229	RP11-248C1.2	-5799	0.00
rs58055098	chr10_89829386_G_C_b38	1.37E-34	C	G	12.2667	ENSG00000152782	PANK1	215163	0.00
rs58055098	chr10_89829386_G_C_b38	7.24E-09	C	G	-5.785	ENSG00000152778	IFIT5	411593	4.52E-05
rs58055098	chr10_89829386_G_C_b38	1.00E-09	C	G	6.1093	ENSG00000107798	LIPA	515323	1.32E-05
rs58055098	chr10_89829386_G_C_b38	8.06E-61	C	G	16.4525	ENSG00000138182	KIF20B	91110	0.00
rs58100002	chr3_52555824_T_C_b38	1.88E-14	C	T	-7.6583	ENSG00000168273	SMIM4	-801	0.00
rs58100002	chr3_52555824_T_C_b38	1.64E-09	C	T	-6.0295	ENSG00000163931	TKT	-684555	1.31E-05
rs58100002	chr3_52555824_T_C_b38	3.23E-74	C	T	18.2256	ENSG00000168268	NT5DC2	26112	0.00
rs58100002	chr3_52555824_T_C_b38	1.89E-07	C	T	-5.2101	ENSG00000242142	SERBP1P3	-508863	5.88E-04
rs58100002	chr3_52555824_T_C_b38	1.27E-05	C	T	4.3649	ENSG00000163933	RFT1	-553648	3.28E-02
rs58564212	chr3_52767623_C_A_b38	4.04E-10	A	C	-6.2522	ENSG00000163931	TKT	-472756	0.00
rs58564212	chr3_52767623_C_A_b38	3.83E-65	A	C	17.0447	ENSG00000168268	NT5DC2	237911	0.00
rs58564212	chr3_52767623_C_A_b38	3.21E-13	A	C	-7.2851	ENSG00000168273	SMIM4	210998	0.00
rs58564212	chr3_52767623_C_A_b38	1.25E-07	A	C	-5.2864	ENSG00000242142	SERBP1P3	-297064	3.89E-04
rs58725214	chr3_52559198_T_C_b38	1.88E-14	C	T	-7.6583	ENSG00000168273	SMIM4	2573	0.00
rs58725214	chr3_52559198_T_C_b38	1.34E-05	C	T	4.353	ENSG00000163933	RFT1	-550274	3.45E-02

rs58725214	chr3_52559198_T_C_b38	3.23E-74	C	T	18.2256	ENSG00000168268	NT5DC2	29486	0.00
rs58725214	chr3_52559198_T_C_b38	1.89E-07	C	T	-5.2101	ENSG00000242142	SERBP1P3	-505489	5.88E-04
rs58725214	chr3_52559198_T_C_b38	1.61E-09	C	T	-6.0326	ENSG00000163931	TKT	-681181	1.31E-05
rs59638016	chr3_52737856_T_C_b38	2.15E-65	T	C	17.0786	ENSG00000168268	NT5DC2	208144	0.00
rs59638016	chr3_52737856_T_C_b38	1.46E-07	T	C	-5.2577	ENSG00000242142	SERBP1P3	-326831	4.51E-04
rs59638016	chr3_52737856_T_C_b38	2.14E-13	T	C	-7.3395	ENSG00000168273	SMIM4	181231	0.00
rs59638016	chr3_52737856_T_C_b38	1.62E-09	T	C	-6.0324	ENSG00000163931	TKT	-502523	1.31E-05
rs60040519	chr3_52641565_T_C_b38	6.38E-11	C	T	-6.5345	ENSG00000163931	TKT	-598814	0.00
rs60040519	chr3_52641565_T_C_b38	4.39E-70	C	T	17.6975	ENSG00000168268	NT5DC2	111853	0.00
rs60040519	chr3_52641565_T_C_b38	3.14E-08	C	T	-5.5334	ENSG00000242142	SERBP1P3	-423122	1.34E-04
rs60040519	chr3_52641565_T_C_b38	2.51E-11	C	T	-6.6726	ENSG00000168273	SMIM4	84940	0.00
rs60040519	chr3_52641565_T_C_b38	4.31E-06	C	T	4.5961	ENSG00000163933	RFT1	-467907	1.15E-02
rs60415551	chr3_52538632_T_A_b38	2.83E-14	A	T	-7.6057	ENSG00000168273	SMIM4	-17993	0.00
rs60415551	chr3_52538632_T_A_b38	1.21E-74	A	T	18.2794	ENSG00000168268	NT5DC2	8920	0.00
rs60415551	chr3_52538632_T_A_b38	9.99E-06	A	T	4.4175	ENSG00000163933	RFT1	-570840	2.59E-02
rs60415551	chr3_52538632_T_A_b38	1.65E-07	A	T	-5.2353	ENSG00000242142	SERBP1P3	-526055	5.14E-04
rs60415551	chr3_52538632_T_A_b38	6.40E-09	A	T	-5.8059	ENSG00000163931	TKT	-701747	4.52E-05
rs60823713	chr3_52539085_G_A_b38	1.65E-07	A	G	-5.2353	ENSG00000242142	SERBP1P3	-525602	5.14E-04
rs60823713	chr3_52539085_G_A_b38	9.99E-06	A	G	4.4175	ENSG00000163933	RFT1	-570387	2.59E-02
rs60823713	chr3_52539085_G_A_b38	6.40E-09	A	G	-5.8059	ENSG00000163931	TKT	-701294	4.52E-05
rs60823713	chr3_52539085_G_A_b38	1.31E-74	A	G	18.275	ENSG00000168268	NT5DC2	9373	0.00
rs60823713	chr3_52539085_G_A_b38	2.77E-14	A	G	-7.6088	ENSG00000168273	SMIM4	-17540	0.00
rs60830326	chr3_52639775_T_C_b38	4.39E-70	T	C	17.6975	ENSG00000168268	NT5DC2	110063	0.00
rs60830326	chr3_52639775_T_C_b38	2.51E-11	T	C	-6.6726	ENSG00000168273	SMIM4	83150	0.00
rs60830326	chr3_52639775_T_C_b38	6.38E-11	T	C	-6.5345	ENSG00000163931	TKT	-600604	0.00
rs60830326	chr3_52639775_T_C_b38	4.31E-06	T	C	4.5961	ENSG00000163933	RFT1	-469697	1.15E-02
rs60830326	chr3_52639775_T_C_b38	3.14E-08	T	C	-5.5334	ENSG00000242142	SERBP1P3	-424912	1.34E-04
rs61220667	chr3_52710420_T_G_b38	1.67E-05	G	T	4.3055	ENSG00000163933	RFT1	-399052	4.21E-02
rs61220667	chr3_52710420_T_G_b38	1.01E-73	G	T	18.1632	ENSG00000168268	NT5DC2	180708	0.00
rs61220667	chr3_52710420_T_G_b38	3.14E-14	G	T	-7.5924	ENSG00000168273	SMIM4	153795	0.00
rs61220667	chr3_52710420_T_G_b38	1.52E-09	G	T	-6.0425	ENSG00000163931	TKT	-529959	1.31E-05
rs61220667	chr3_52710420_T_G_b38	1.71E-07	G	T	-5.2282	ENSG00000242142	SERBP1P3	-354267	5.26E-04

rs61827378	chr1_175046714_T_C_b38	2.05E-14	C	T	7.6477	ENSG00000235750	KIAA0040	-128279	0.00
rs6445536	chr3_52751167_G_A_b38	1.13E-07	A	G	-5.3041	ENSG00000242142	SERBP1P3	-313520	3.65E-04
rs6445536	chr3_52751167_G_A_b38	2.82E-10	A	G	-6.3081	ENSG00000163931	TKT	-489212	0.00
rs6445536	chr3_52751167_G_A_b38	5.44E-14	A	G	-7.5208	ENSG00000168273	SMIM4	194542	0.00
rs6445536	chr3_52751167_G_A_b38	1.48E-72	A	G	18.0153	ENSG00000168268	NT5DC2	221455	0.00
rs6467310	chr7_130628875_T_C_b38	3.18E-13	T	C	-7.2863	ENSG00000106484	MEST	177655	0.00
rs6467310	chr7_130628875_T_C_b38	3.27E-310	T	C	-42.0493	ENSG00000158623	COPG2	63901	0.00
rs6467311	chr7_130655148_C_A_b38	3.27E-310	C	A	-42.0719	ENSG00000158623	COPG2	90145	0.00
rs6467311	chr7_130655148_C_A_b38	8.30E-14	C	A	-7.4653	ENSG00000106484	MEST	203899	0.00
rs6583662	chr10_89826359_G_A_b38	3.27E-310	A	G	40.3335	ENSG00000232229	RP11-248C1.2	-8826	0.00
rs6583662	chr10_89826359_G_A_b38	2.99E-60	A	G	16.3729	ENSG00000138182	KIF20B	88083	0.00
rs6583662	chr10_89826359_G_A_b38	1.14E-08	A	G	-5.7078	ENSG00000152778	IFIT5	408566	6.43E-05
rs6583662	chr10_89826359_G_A_b38	3.98E-14	A	G	7.5618	ENSG00000107798	LIPA	512296	0.00
rs6583662	chr10_89826359_G_A_b38	2.87E-43	A	G	13.7916	ENSG00000152782	PANK1	212136	0.00
rs6769720	chr3_52730334_T_G_b38	3.69E-10	G	T	-6.2665	ENSG00000163931	TKT	-510045	0.00
rs6769720	chr3_52730334_T_G_b38	1.40E-72	G	T	18.0183	ENSG00000168268	NT5DC2	200622	0.00
rs6769720	chr3_52730334_T_G_b38	1.21E-07	G	T	-5.2919	ENSG00000242142	SERBP1P3	-334353	3.71E-04
rs6769720	chr3_52730334_T_G_b38	3.09E-14	G	T	-7.5945	ENSG00000168273	SMIM4	173709	0.00
rs6780005	chr3_52602599_G_C_b38	8.56E-12	G	C	-6.8287	ENSG00000163931	TKT	-637780	0.00
rs6780005	chr3_52602599_G_C_b38	1.89E-07	G	C	-5.2101	ENSG00000242142	SERBP1P3	-462088	5.88E-04
rs6780005	chr3_52602599_G_C_b38	8.18E-06	G	C	4.4605	ENSG00000163933	RFT1	-506873	2.13E-02
rs6780005	chr3_52602599_G_C_b38	4.56E-82	G	C	19.1892	ENSG00000168268	NT5DC2	72887	0.00
rs6780005	chr3_52602599_G_C_b38	2.36E-14	G	C	-7.6291	ENSG00000168273	SMIM4	45974	0.00
rs6867085	chr5_107851606_C_A_b38	4.31E-35	C	A	12.3599	ENSG00000145743	FBXL17	-268960	0.00
rs7083051	chr10_89826284_T_C_b38	8.17E-10	C	T	-6.1415	ENSG00000152778	IFIT5	408491	1.32E-05
rs7083051	chr10_89826284_T_C_b38	2.61E-47	C	T	14.4473	ENSG00000152782	PANK1	212061	0.00
rs7083051	chr10_89826284_T_C_b38	3.27E-310	C	T	40.8367	ENSG00000232229	RP11-248C1.2	-8901	0.00
rs7083051	chr10_89826284_T_C_b38	1.40E-59	C	T	16.2789	ENSG00000138182	KIF20B	88008	0.00
rs7083051	chr10_89826284_T_C_b38	1.09E-13	C	T	7.4294	ENSG00000107798	LIPA	512221	0.00
rs71414848	chr2_70946048_T_C_b38	3.01E-09	C	T	5.9312	ENSG00000231386	AC007395.4	134910	2.60E-05
rs71414848	chr2_70946048_T_C_b38	1.88E-05	C	T	4.2786	ENSG00000116031	CD207	113029	4.70E-02
rs71414848	chr2_70946048_T_C_b38	4.81E-06	C	T	4.5728	ENSG00000124370	MCEE	-173913	1.30E-02

rs71414848	chr2_70946048_T_C_b38	3.74E-10	C	T	6.2648	ENSG00000124357	NAGK	-126026	0.00
rs71414848	chr2_70946048_T_C_b38	3.16E-53	C	T	15.3575	ENSG00000152672	CLEC4F	131425	0.00
rs72947580	chr3_52581467_G_A_b38	9.97E-89	A	G	19.9706	ENSG00000168268	NT5DC2	51755	0.00
rs72947580	chr3_52581467_G_A_b38	1.82E-06	A	G	4.7732	ENSG00000163933	RFT1	-528005	4.98E-03
rs72947580	chr3_52581467_G_A_b38	1.89E-07	A	G	-5.2101	ENSG00000242142	SERBP1P3	-483220	5.88E-04
rs72947580	chr3_52581467_G_A_b38	3.71E-15	A	G	-7.8641	ENSG00000168273	SMIM4	24842	0.00
rs72947580	chr3_52581467_G_A_b38	1.39E-10	A	G	-6.4171	ENSG00000163931	TKT	-658912	0.00
rs72947589	chr3_52585530_T_A_b38	1.14E-74	A	T	18.2825	ENSG00000168268	NT5DC2	55818	0.00
rs72947589	chr3_52585530_T_A_b38	1.61E-09	A	T	-6.0326	ENSG00000163931	TKT	-654849	1.31E-05
rs72947589	chr3_52585530_T_A_b38	1.89E-07	A	T	-5.2101	ENSG00000242142	SERBP1P3	-479157	5.88E-04
rs72947589	chr3_52585530_T_A_b38	1.58E-14	A	T	-7.6807	ENSG00000168273	SMIM4	28905	0.00
rs72947589	chr3_52585530_T_A_b38	1.60E-05	A	T	4.315	ENSG00000163933	RFT1	-523942	4.05E-02
rs72950432	chr3_52662336_T_C_b38	2.40E-74	C	T	18.242	ENSG00000168268	NT5DC2	132624	0.00
rs72950432	chr3_52662336_T_C_b38	2.04E-07	C	T	-5.1952	ENSG00000242142	SERBP1P3	-402351	6.25E-04
rs72950432	chr3_52662336_T_C_b38	3.01E-14	C	T	-7.5977	ENSG00000168273	SMIM4	105711	0.00
rs72950432	chr3_52662336_T_C_b38	1.43E-09	C	T	-6.0523	ENSG00000163931	TKT	-578043	1.31E-05
rs72960237	chr3_52723637_G_A_b38	1.52E-09	A	G	-6.0419	ENSG00000163931	TKT	-516742	1.31E-05
rs72960237	chr3_52723637_G_A_b38	1.49E-07	A	G	-5.2537	ENSG00000242142	SERBP1P3	-341050	4.70E-04
rs72960237	chr3_52723637_G_A_b38	1.85E-13	A	G	-7.3591	ENSG00000168273	SMIM4	167012	0.00
rs72960237	chr3_52723637_G_A_b38	2.13E-65	A	G	17.0788	ENSG00000168268	NT5DC2	193925	0.00
rs72960240	chr3_52725117_C_A_b38	2.10E-72	C	A	17.996	ENSG00000168268	NT5DC2	195405	0.00
rs72960240	chr3_52725117_C_A_b38	3.46E-14	C	A	-7.5796	ENSG00000168273	SMIM4	168492	0.00
rs72960240	chr3_52725117_C_A_b38	3.64E-10	C	A	-6.2685	ENSG00000163931	TKT	-515262	0.00
rs72960240	chr3_52725117_C_A_b38	1.27E-07	C	A	-5.2836	ENSG00000242142	SERBP1P3	-339570	3.96E-04
rs72960274	chr3_52755927_T_C_b38	6.85E-10	T	C	-6.1694	ENSG00000163931	TKT	-484452	0.00
rs72960274	chr3_52755927_T_C_b38	1.27E-07	T	C	-5.2824	ENSG00000242142	SERBP1P3	-308760	3.96E-04
rs72960274	chr3_52755927_T_C_b38	3.02E-13	T	C	-7.2935	ENSG00000168273	SMIM4	199302	0.00
rs72960274	chr3_52755927_T_C_b38	3.99E-65	T	C	17.0423	ENSG00000168268	NT5DC2	226215	0.00
rs72960281	chr3_52768855_T_C_b38	8.25E-11	T	C	-6.496	ENSG00000163931	TKT	-471524	0.00
rs72960281	chr3_52768855_T_C_b38	1.11E-07	T	C	-5.308	ENSG00000242142	SERBP1P3	-295832	3.58E-04
rs72960281	chr3_52768855_T_C_b38	4.33E-14	T	C	-7.5505	ENSG00000168273	SMIM4	212230	0.00
rs72960281	chr3_52768855_T_C_b38	6.98E-72	T	C	17.9294	ENSG00000168268	NT5DC2	239143	0.00

rs72960285	chr3_52773800_T_G_b38	1.55E-13	G	T	-7.3828	ENSG00000168273	SMIM4	217175	0.00
rs72960285	chr3_52773800_T_G_b38	2.88E-08	G	T	-5.5485	ENSG00000242142	SERBP1P3	-290887	1.21E-04
rs72960285	chr3_52773800_T_G_b38	2.07E-11	G	T	-6.7009	ENSG00000163931	TKT	-466579	0.00
rs72960285	chr3_52773800_T_G_b38	1.45E-70	G	T	17.7598	ENSG00000168268	NT5DC2	244088	0.00
rs72965177	chr3_52601048_T_C_b38	1.61E-09	C	T	-6.0326	ENSG00000163931	TKT	-639331	1.31E-05
rs72965177	chr3_52601048_T_C_b38	1.13E-74	C	T	18.2833	ENSG00000168268	NT5DC2	71336	0.00
rs72965177	chr3_52601048_T_C_b38	1.42E-05	C	T	4.3412	ENSG00000163933	RFT1	-508424	3.64E-02
rs72965177	chr3_52601048_T_C_b38	1.89E-07	C	T	-5.2101	ENSG00000242142	SERBP1P3	-463639	5.88E-04
rs72965177	chr3_52601048_T_C_b38	1.41E-14	C	T	-7.6956	ENSG00000168273	SMIM4	44423	0.00
rs73837804	chr3_52539996_G_C_b38	2.97E-10	C	G	-6.3004	ENSG00000163931	TKT	-700383	0.00
rs73837804	chr3_52539996_G_C_b38	2.82E-11	C	G	-6.6557	ENSG00000168273	SMIM4	-16629	0.00
rs73837804	chr3_52539996_G_C_b38	3.85E-06	C	G	4.6193	ENSG00000163933	RFT1	-569476	1.03E-02
rs73837804	chr3_52539996_G_C_b38	2.92E-08	C	G	-5.5461	ENSG00000242142	SERBP1P3	-524691	1.21E-04
rs73837804	chr3_52539996_G_C_b38	1.72E-70	C	G	17.7503	ENSG00000168268	NT5DC2	10284	0.00
rs73839126	chr3_52672109_T_C_b38	1.26E-12	T	C	-7.0988	ENSG00000168273	SMIM4	115484	0.00
rs73839126	chr3_52672109_T_C_b38	3.14E-08	T	C	-5.5334	ENSG00000242142	SERBP1P3	-392578	1.34E-04
rs73839126	chr3_52672109_T_C_b38	1.59E-71	T	C	17.8835	ENSG00000168268	NT5DC2	142397	0.00
rs73839126	chr3_52672109_T_C_b38	8.26E-11	T	C	-6.4957	ENSG00000163931	TKT	-568270	0.00
rs73839126	chr3_52672109_T_C_b38	6.18E-06	T	C	4.5201	ENSG00000163933	RFT1	-437363	1.65E-02
rs74429279	chr3_52732241_G_C_b38	2.84E-13	G	C	-7.3016	ENSG00000168273	SMIM4	175616	0.00
rs74429279	chr3_52732241_G_C_b38	3.24E-10	G	C	-6.2869	ENSG00000163931	TKT	-508138	0.00
rs74429279	chr3_52732241_G_C_b38	2.34E-74	G	C	18.2433	ENSG00000168268	NT5DC2	202529	0.00
rs74429279	chr3_52732241_G_C_b38	9.46E-08	G	C	-5.3367	ENSG00000242142	SERBP1P3	-332446	3.09E-04
rs74429279	chr3_52732241_G_C_b38	1.12E-05	G	C	4.3928	ENSG00000163933	RFT1	-377231	2.90E-02
rs74464684	chr3_53081611_T_C_b38	8.97E-16	T	C	-8.0402	ENSG00000163931	TKT	-158768	0.00
rs74464684	chr3_53081611_T_C_b38	1.42E-05	T	C	-4.3403	ENSG00000242142	SERBP1P3	16924	3.65E-02
rs74464684	chr3_53081611_T_C_b38	6.54E-49	T	C	14.6991	ENSG00000168268	NT5DC2	551899	0.00
rs74464684	chr3_53081611_T_C_b38	4.80E-07	T	C	-5.0338	ENSG00000168273	SMIM4	524986	1.47E-03
rs74582920	chr3_105935398_T_C_b38	4.95E-14	T	C	-7.5332	ENSG00000114423	CBLB	172895	0.00
rs74582920	chr3_105935398_T_C_b38	8.67E-15	T	C	7.7575	ENSG00000170017	ALCAM	463497	0.00
rs74686130	chr3_52566122_T_C_b38	6.14E-06	T	C	4.5218	ENSG00000163933	RFT1	-543350	1.63E-02
rs74686130	chr3_52566122_T_C_b38	1.33E-07	T	C	-5.274	ENSG00000242142	SERBP1P3	-498565	4.21E-04

rs74686130	chr3_52566122_T_C_b38	6.84E-11	T	C	-6.5242	ENSG00000163931	TKT	-674257	0.00
rs74686130	chr3_52566122_T_C_b38	5.24E-11	T	C	-6.5639	ENSG00000168273	SMIM4	9497	0.00
rs74686130	chr3_52566122_T_C_b38	3.07E-68	T	C	17.4566	ENSG00000168268	NT5DC2	36410	0.00
rs7516036	chr1_200727825_G_A_b38	4.14E-17	A	G	-8.4086	ENSG00000229191	RP11-168O16.1	-298545	0.00
rs7516036	chr1_200727825_G_A_b38	1.46E-05	A	G	-4.3341	ENSG00000163362	C1orf106	-175566	3.73E-02
rs7516036	chr1_200727825_G_A_b38	3.10E-122	A	G	23.5117	ENSG00000118200	CAMSAP2	-72306	0.00
rs7516036	chr1_200727825_G_A_b38	3.28E-14	A	G	-7.5865	ENSG00000170128	GPR25	-145741	0.00
rs7516036	chr1_200727825_G_A_b38	2.49E-52	A	G	-15.2228	ENSG00000118197	DDX59	80893	0.00
rs7519783	chr1_200715929_G_A_b38	1.86E-52	G	A	-15.242	ENSG00000118197	DDX59	68997	0.00
rs7519783	chr1_200715929_G_A_b38	1.61E-126	G	A	23.9269	ENSG00000118200	CAMSAP2	-84202	0.00
rs7519783	chr1_200715929_G_A_b38	1.42E-13	G	A	-7.3945	ENSG00000170128	GPR25	-157637	0.00
rs7519783	chr1_200715929_G_A_b38	4.60E-18	G	A	-8.6628	ENSG00000229191	RP11-168O16.1	-310441	0.00
rs75211389	chr15_66164538_G_A_b38	4.90E-19	G	A	8.9147	ENSG00000166938	DIS3L	-149019	0.00
rs75321479	chr3_52744194_G_A_b38	2.77E-08	G	A	-5.5555	ENSG00000242142	SERBP1P3	-320493	1.21E-04
rs75321479	chr3_52744194_G_A_b38	1.75E-12	G	A	-7.0534	ENSG00000168273	SMIM4	187569	0.00
rs75321479	chr3_52744194_G_A_b38	3.66E-71	G	A	17.8368	ENSG00000168268	NT5DC2	214482	0.00
rs75321479	chr3_52744194_G_A_b38	8.45E-11	G	A	-6.4924	ENSG00000163931	TKT	-496185	0.00
rs75321479	chr3_52744194_G_A_b38	6.51E-06	G	A	4.5092	ENSG00000163933	RFT1	-365278	1.72E-02
rs75713246	chr3_52571964_G_A_b38	1.45E-14	A	G	-7.6916	ENSG00000168273	SMIM4	15339	0.00
rs75713246	chr3_52571964_G_A_b38	1.61E-09	A	G	-6.0326	ENSG00000163931	TKT	-668415	1.31E-05
rs75713246	chr3_52571964_G_A_b38	1.89E-07	A	G	-5.2101	ENSG00000242142	SERBP1P3	-492723	5.88E-04
rs75713246	chr3_52571964_G_A_b38	1.54E-05	A	G	4.3231	ENSG00000163933	RFT1	-537508	3.91E-02
rs75713246	chr3_52571964_G_A_b38	1.34E-74	A	G	18.2738	ENSG00000168268	NT5DC2	42252	0.00
rs7619643	chr3_52754103_G_A_b38	2.51E-12	A	G	-7.0025	ENSG00000163931	TKT	-486276	0.00
rs7619643	chr3_52754103_G_A_b38	5.51E-09	A	G	-5.8309	ENSG00000055955	ITIH4	-68124	3.88E-05
rs7619643	chr3_52754103_G_A_b38	1.08E-07	A	G	-5.3123	ENSG00000242142	SERBP1P3	-310584	3.33E-04
rs7619643	chr3_52754103_G_A_b38	2.71E-06	A	G	4.6921	ENSG00000163933	RFT1	-355369	7.16E-03
rs7619643	chr3_52754103_G_A_b38	3.54E-12	A	G	-6.9546	ENSG00000168273	SMIM4	197478	0.00
rs7619643	chr3_52754103_G_A_b38	1.03E-62	A	G	16.7143	ENSG00000168268	NT5DC2	224391	0.00
rs76252265	chr3_52846426_G_A_b38	1.65E-09	A	G	-6.0293	ENSG00000168273	SMIM4	289801	1.31E-05
rs76252265	chr3_52846426_G_A_b38	3.96E-06	A	G	4.6138	ENSG00000163933	RFT1	-263046	1.06E-02
rs76252265	chr3_52846426_G_A_b38	1.49E-09	A	G	-6.0449	ENSG00000242142	SERBP1P3	-218261	1.31E-05

rs76252265	chr3_52846426_G_A_b38	7.50E-14	A	G	-7.4788	ENSG00000163931	TKT	-393953	0.00
rs76252265	chr3_52846426_G_A_b38	6.21E-64	A	G	16.8811	ENSG00000168268	NT5DC2	316714	0.00
rs764636	chr3_52732681_G_A_b38	1.45E-65	A	G	17.1013	ENSG00000168268	NT5DC2	202969	0.00
rs764636	chr3_52732681_G_A_b38	1.90E-13	A	G	-7.3559	ENSG00000168273	SMIM4	176056	0.00
rs764636	chr3_52732681_G_A_b38	1.60E-09	A	G	-6.0344	ENSG00000163931	TKT	-507698	1.31E-05
rs764636	chr3_52732681_G_A_b38	1.36E-07	A	G	-5.2703	ENSG00000242142	SERBP1P3	-332006	4.20E-04
rs7647992	chr3_52597328_T_G_b38	1.48E-05	T	G	4.3316	ENSG00000163933	RFT1	-512144	3.77E-02
rs7647992	chr3_52597328_T_G_b38	1.58E-14	T	G	-7.6807	ENSG00000168273	SMIM4	40703	0.00
rs7647992	chr3_52597328_T_G_b38	1.89E-07	T	G	-5.2101	ENSG00000242142	SERBP1P3	-467359	5.88E-04
rs7647992	chr3_52597328_T_G_b38	1.34E-74	T	G	18.2738	ENSG00000168268	NT5DC2	67616	0.00
rs7647992	chr3_52597328_T_G_b38	1.61E-09	T	G	-6.0326	ENSG00000163931	TKT	-643051	1.31E-05
rs7648016	chr3_52580394_T_C_b38	3.65E-75	C	T	18.3447	ENSG00000168268	NT5DC2	50682	0.00
rs7648016	chr3_52580394_T_C_b38	1.21E-05	C	T	4.3767	ENSG00000163933	RFT1	-529078	3.12E-02
rs7648016	chr3_52580394_T_C_b38	1.99E-14	C	T	-7.6512	ENSG00000168273	SMIM4	23769	0.00
rs7648016	chr3_52580394_T_C_b38	1.63E-09	C	T	-6.0312	ENSG00000163931	TKT	-659985	1.31E-05
rs7648016	chr3_52580394_T_C_b38	1.89E-07	C	T	-5.2101	ENSG00000242142	SERBP1P3	-484293	5.88E-04
rs76553845	chr3_53024096_G_A_b38	4.12E-07	G	A	-5.0631	ENSG00000168273	SMIM4	467471	1.22E-03
rs76553845	chr3_53024096_G_A_b38	4.40E-45	G	A	14.0897	ENSG00000168268	NT5DC2	494384	0.00
rs76553845	chr3_53024096_G_A_b38	2.18E-12	G	A	-7.0223	ENSG00000163931	TKT	-216283	0.00
rs76759198	chr3_52563010_T_C_b38	6.56E-11	T	C	-6.5304	ENSG00000168273	SMIM4	6385	0.00
rs76759198	chr3_52563010_T_C_b38	1.33E-07	T	C	-5.274	ENSG00000242142	SERBP1P3	-501677	4.21E-04
rs76759198	chr3_52563010_T_C_b38	1.43E-10	T	C	-6.4131	ENSG00000163931	TKT	-677369	0.00
rs76759198	chr3_52563010_T_C_b38	6.89E-06	T	C	4.4972	ENSG00000163933	RFT1	-546462	1.82E-02
rs76759198	chr3_52563010_T_C_b38	7.21E-67	T	C	17.2755	ENSG00000168268	NT5DC2	33298	0.00
rs76974457	chr3_52571795_G_A_b38	5.31E-74	A	G	18.1985	ENSG00000168268	NT5DC2	42083	0.00
rs76974457	chr3_52571795_G_A_b38	2.05E-14	A	G	-7.6474	ENSG00000168273	SMIM4	15170	0.00
rs76974457	chr3_52571795_G_A_b38	1.89E-07	A	G	-5.2101	ENSG00000242142	SERBP1P3	-492892	5.88E-04
rs76974457	chr3_52571795_G_A_b38	1.33E-05	A	G	4.3545	ENSG00000163933	RFT1	-537677	3.43E-02
rs76974457	chr3_52571795_G_A_b38	1.55E-09	A	G	-6.0391	ENSG00000163931	TKT	-668584	1.31E-05
rs77711437	chr3_52590493_T_G_b38	6.33E-70	G	T	17.677	ENSG00000168268	NT5DC2	60781	0.00
rs77711437	chr3_52590493_T_G_b38	4.93E-06	G	T	4.5679	ENSG00000163933	RFT1	-518979	1.33E-02
rs77711437	chr3_52590493_T_G_b38	3.75E-11	G	T	-6.6138	ENSG00000168273	SMIM4	33868	0.00

rs77711437	chr3_52590493_T_G_b38	3.67E-08	G	T	-5.5059	ENSG00000242142	SERBP1P3	-474194	1.40E-04
rs77711437	chr3_52590493_T_G_b38	9.15E-11	G	T	-6.4802	ENSG00000163931	TKT	-649886	0.00
rs78014137	chr3_52742762_T_C_b38	2.04E-12	C	T	-7.0319	ENSG00000168273	SMIM4	186137	0.00
rs78014137	chr3_52742762_T_C_b38	6.23E-06	C	T	4.5188	ENSG00000163933	RFT1	-366710	1.65E-02
rs78014137	chr3_52742762_T_C_b38	3.14E-71	C	T	17.8455	ENSG00000168268	NT5DC2	213050	0.00
rs78014137	chr3_52742762_T_C_b38	2.77E-08	C	T	-5.5555	ENSG00000242142	SERBP1P3	-321925	1.21E-04
rs78014137	chr3_52742762_T_C_b38	8.45E-11	C	T	-6.4924	ENSG00000163931	TKT	-497617	0.00
rs78059582	chr3_52659200_T_C_b38	6.29E-06	C	T	4.5165	ENSG00000163933	RFT1	-450272	1.67E-02
rs78059582	chr3_52659200_T_C_b38	4.25E-11	C	T	-6.5951	ENSG00000168273	SMIM4	102575	0.00
rs78059582	chr3_52659200_T_C_b38	6.19E-11	C	T	-6.539	ENSG00000163931	TKT	-581179	0.00
rs78059582	chr3_52659200_T_C_b38	1.81E-67	C	T	17.3549	ENSG00000168268	NT5DC2	129488	0.00
rs78059582	chr3_52659200_T_C_b38	2.36E-07	C	T	-5.1688	ENSG00000242142	SERBP1P3	-405487	7.30E-04
rs78735727	chr3_52606100_G_A_b38	2.08E-11	A	G	-6.7001	ENSG00000168273	SMIM4	49475	0.00
rs78735727	chr3_52606100_G_A_b38	3.37E-08	A	G	-5.5208	ENSG00000242142	SERBP1P3	-458587	1.34E-04
rs78735727	chr3_52606100_G_A_b38	3.39E-13	A	G	-7.278	ENSG00000163931	TKT	-634279	0.00
rs78735727	chr3_52606100_G_A_b38	2.84E-06	A	G	4.6826	ENSG00000163933	RFT1	-503372	7.64E-03
rs78735727	chr3_52606100_G_A_b38	1.50E-78	A	G	18.7635	ENSG00000168268	NT5DC2	76388	0.00
rs7895530	chr10_89827592_T_C_b38	3.08E-17	T	C	8.4437	ENSG00000107798	LIPA	513529	0.00
rs7895530	chr10_89827592_T_C_b38	1.08E-06	T	C	-4.8757	ENSG00000152778	IFIT5	409799	3.08E-03
rs7895530	chr10_89827592_T_C_b38	1.29E-270	T	C	35.1472	ENSG00000232229	RP11-248C1.2	-7593	0.00
rs7895530	chr10_89827592_T_C_b38	2.15E-59	T	C	16.2524	ENSG00000152782	PANK1	213369	0.00
rs7895530	chr10_89827592_T_C_b38	1.18E-42	T	C	13.689	ENSG00000138182	KIF20B	89316	0.00
rs7895851	chr10_89827644_G_A_b38	2.03E-09	A	G	-5.995	ENSG00000152778	IFIT5	409851	1.96E-05
rs7895851	chr10_89827644_G_A_b38	1.32E-59	A	G	16.2823	ENSG00000138182	KIF20B	89368	0.00
rs7895851	chr10_89827644_G_A_b38	3.55E-46	A	G	14.2662	ENSG00000152782	PANK1	213421	0.00
rs7895851	chr10_89827644_G_A_b38	3.27E-310	A	G	40.8789	ENSG00000232229	RP11-248C1.2	-7541	0.00
rs7895851	chr10_89827644_G_A_b38	4.49E-14	A	G	7.546	ENSG00000107798	LIPA	513581	0.00
rs7895863	chr10_89827672_G_A_b38	1.52E-59	A	G	16.2735	ENSG00000138182	KIF20B	89396	0.00
rs7895863	chr10_89827672_G_A_b38	9.64E-10	A	G	-6.115	ENSG00000152778	IFIT5	409879	1.32E-05
rs7895863	chr10_89827672_G_A_b38	9.12E-14	A	G	7.4531	ENSG00000107798	LIPA	513609	0.00
rs7895863	chr10_89827672_G_A_b38	8.58E-47	A	G	14.3651	ENSG00000152782	PANK1	213449	0.00
rs7895863	chr10_89827672_G_A_b38	3.27E-310	A	G	40.8527	ENSG00000232229	RP11-248C1.2	-7513	0.00

rs7899499	chr10_89828244_T_C_b38	1.18E-09	T	C	-6.0831	ENSG00000152778	IFIT5	410451	1.31E-05
rs7899499	chr10_89828244_T_C_b38	7.42E-60	T	C	16.3175	ENSG00000138182	KIF20B	89968	0.00
rs7899499	chr10_89828244_T_C_b38	7.19E-14	T	C	7.4845	ENSG00000107798	LIPA	514181	0.00
rs7899499	chr10_89828244_T_C_b38	2.00E-47	T	C	14.4656	ENSG00000152782	PANK1	214021	0.00
rs7899499	chr10_89828244_T_C_b38	3.27E-310	T	C	40.5974	ENSG00000232229	RP11-248C1.2	-6941	0.00
rs7918018	chr10_89825521_G_C_b38	1.02E-59	G	C	16.298	ENSG00000138182	KIF20B	87245	0.00
rs7918018	chr10_89825521_G_C_b38	8.43E-14	G	C	7.4636	ENSG00000107798	LIPA	511458	0.00
rs7918018	chr10_89825521_G_C_b38	2.56E-47	G	C	14.4487	ENSG00000152782	PANK1	211298	0.00
rs7918018	chr10_89825521_G_C_b38	3.27E-310	G	C	40.8324	ENSG00000232229	RP11-248C1.2	-9664	0.00
rs7918018	chr10_89825521_G_C_b38	8.82E-10	G	C	-6.1292	ENSG00000152778	IFIT5	407728	1.32E-05
rs79658570	chr3_52574513_G_A_b38	1.46E-66	G	A	17.2349	ENSG00000168268	NT5DC2	44801	0.00
rs79658570	chr3_52574513_G_A_b38	7.06E-06	G	A	4.492	ENSG00000163933	RFT1	-534959	1.86E-02
rs79658570	chr3_52574513_G_A_b38	5.59E-11	G	A	-6.5543	ENSG00000168273	SMIM4	17888	0.00
rs79658570	chr3_52574513_G_A_b38	6.87E-11	G	A	-6.5234	ENSG00000163931	TKT	-665866	0.00
rs79658570	chr3_52574513_G_A_b38	3.08E-07	G	A	-5.1183	ENSG00000242142	SERBP1P3	-490174	9.44E-04
rs79705974	chr3_52702426_G_A_b38	1.68E-71	A	G	17.8805	ENSG00000168268	NT5DC2	172714	0.00
rs79705974	chr3_52702426_G_A_b38	3.26E-08	A	G	-5.5268	ENSG00000242142	SERBP1P3	-362261	1.34E-04
rs79705974	chr3_52702426_G_A_b38	9.16E-11	A	G	-6.4801	ENSG00000163931	TKT	-537953	0.00
rs79705974	chr3_52702426_G_A_b38	1.41E-12	A	G	-7.0833	ENSG00000168273	SMIM4	145801	0.00
rs79705974	chr3_52702426_G_A_b38	5.73E-06	A	G	4.5362	ENSG00000163933	RFT1	-407046	1.52E-02
rs79957220	chr3_52637839_T_C_b38	2.23E-71	T	C	17.8645	ENSG00000168268	NT5DC2	108127	0.00
rs79957220	chr3_52637839_T_C_b38	3.32E-08	T	C	-5.5237	ENSG00000242142	SERBP1P3	-426848	1.34E-04
rs79957220	chr3_52637839_T_C_b38	7.01E-11	T	C	-6.5204	ENSG00000163931	TKT	-602540	0.00
rs79957220	chr3_52637839_T_C_b38	8.63E-06	T	C	4.449	ENSG00000163933	RFT1	-471633	2.25E-02
rs79957220	chr3_52637839_T_C_b38	1.22E-12	T	C	-7.1026	ENSG00000168273	SMIM4	81214	0.00
rs80214269	chr3_52654730_T_C_b38	2.68E-12	T	C	-6.9937	ENSG00000168273	SMIM4	98105	0.00
rs80214269	chr3_52654730_T_C_b38	2.24E-08	T	C	-5.5919	ENSG00000242142	SERBP1P3	-409957	1.09E-04
rs80214269	chr3_52654730_T_C_b38	1.66E-10	T	C	-6.3895	ENSG00000163931	TKT	-585649	0.00
rs80214269	chr3_52654730_T_C_b38	3.27E-06	T	C	4.653	ENSG00000163933	RFT1	-454742	8.71E-03
rs80214269	chr3_52654730_T_C_b38	4.89E-71	T	C	17.8207	ENSG00000168268	NT5DC2	125018	0.00
rs80332599	chr3_52556552_T_C_b38	5.42E-11	C	T	-6.5587	ENSG00000168273	SMIM4	-73	0.00
rs80332599	chr3_52556552_T_C_b38	2.83E-68	C	T	17.4613	ENSG00000168268	NT5DC2	26840	0.00

rs80332599	chr3_52556552_T_C_b38	8.11E-11	C	T	-6.4986	ENSG00000163931	TKT	-683827	0.00
rs80332599	chr3_52556552_T_C_b38	6.01E-06	C	T	4.5263	ENSG00000163933	RFT1	-552920	1.59E-02
rs80332599	chr3_52556552_T_C_b38	1.33E-07	C	T	-5.274	ENSG00000242142	SERBP1P3	-508135	4.21E-04
